# Cell-Based Progression of Spiroindoline Phenotypic Hits Leads to the Identification of Compounds with Diverging Parasitological Profiles against the Human Malaria Parasite Plasmodium falciparum

**DOI:** 10.1021/acs.jmedchem.5c00302

**Published:** 2025-05-12

**Authors:** Jean Dam, Grant A. Boyle, André Horatscheck, John G. Woodland, Claire Le Manach, Gurminder Kaur, Dale Taylor, Liezl Krugmann, Mathew Njoroge, Nina Lawrence, Christel Brunschwig, Victor Zdorichenko, Brian Cox, Sergio Wittlin, Thomas W. von Geldern, Dennis Smith, James Duffy, Gregory S. Basarab, Kelly Chibale

**Affiliations:** † Holistic Drug Discovery and Development (H3D) Centre, 37716 University of Cape Town, Rondebosch 7701, South Africa; # South African Medical Research Council Drug Discovery and Development Research Unit, Institute of Infectious Disease and Molecular Medicine, University of Cape Town, Cape Town 7925, South Africa; Δ Institute of Infectious Disease and Molecular Medicine, 37716 University of Cape Town, Rondebosch 7701, South Africa; ‴ Department of Chemistry, Arundel Building 305. School of Life Sciences, 1948 University of Sussex, Falmer, Brighton, Sussex BN1 9RH, United Kingdom; § 30247 Swiss Tropical and Public Health Institute, Kreuzstrasse 2, Allschwil 4106, Switzerland; Ψ University of Basel, Basel 4002, Switzerland; + Medicines for Malaria Venture, ICC, Route de Pré-Bois 20, PO Box 1826, Geneva 1215, Switzerland

## Abstract

In the search for novel chemotypes with high sp^3^ character and activity against the human malaria parasite Plasmodium falciparum, a spiroindoline series was identified from a phenotypic high-throughput screening campaign. The spiroindoline hit **2** displayed good activity against both drug-sensitive and multidrug-resistant *Plasmodium* strains, making it an attractive starting point for hit-to-lead progression. Structure–activity relationship studies led to the identification of a novel pyridylspiroindoline frontrunner (**50**) with improved antiplasmodial activity, aqueous solubility, and microsomal metabolic stability. Data from additional parasitological profiling suggested that **50** likely has a mode of action differing from that of the original spiroindoline hit. Compound **50** showed excellent *in vivo* pharmacokinetics with efficacy being achieved in a humanized immunodeficient NSG mouse P. falciparum infection model. This provided a pharmacological proof-of-concept for this series, making it a valuable starting point for further optimization in the quest for novel antimalarial therapeutics.

## Graphical abstract

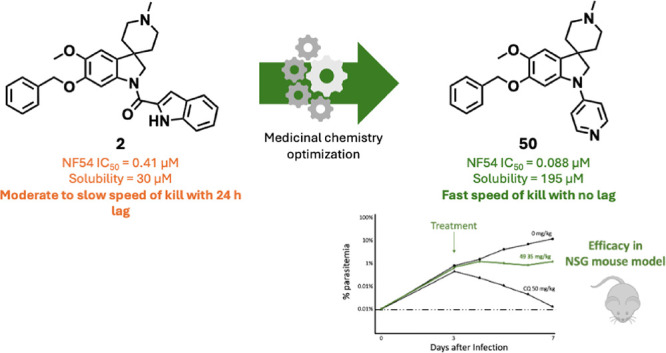

## Introduction

Malaria remains a significant threat in sub-Saharan Africa, particularly for children under the age of 5 years and pregnant women. The 2024 World Malaria Report released by the World Health Organization (WHO) highlighted that 263 million cases of malaria were reported in 2023 from 85 malaria-endemic countries, the vast majority of which were from the African continent. An estimated 597,000 people succumbed to malaria in 2023, and the mortality rate in children under the age of 5 years remained devastatingly high at 74%.^1^

The WHO has implemented a variety of interventions to prevent the spread of malaria such as seasonal malaria chemoprevention (SMC) that lowers the incidence of malaria by 75% in children up to the age of 5 years. Sulfadoxine–pyrimethamine with amodiaquine has been successfully used for SMC for more than a decade.^2^ The number of treated children per cycle has increased dramatically from about 170,000 children in 2012 to 53 million children in 2023.^1^ A curative dose of these antimalarials is administered to asymptomatic children during the malaria season (children are not tested for malaria before SMC administration), ideally every 28 days. In addition, the regular distribution of new classes of long-lasting insecticide-treated bed-nets and indoor residual spraying containing next-generation insecticides was approved by the WHO in 2023.^2^ These interventions have significantly reduced cases globally, but these alone will not eliminate malaria. The first approved vaccine against P. falciparum was the preerythrocytic vaccine RTS,S/AS01 developed by GlaxoSmithKline. Since October 2021, the WHO has recommended the RTS,S/AS01 malaria vaccine for children living in areas with moderate to high malaria transmission.^1,3^ However, this recombinant vaccine is only 36% effective after four treatments in children 5–17 months of age.^4^ A more recent vaccine, R21/Matrix-M, developed by Oxford University, is the first vaccine to exceed the WHO-specified target of ≥75% efficacy.^5^ In October 2023, the WHO recommended this vaccine to be used alongside the RTS,S/AS01 vaccine for the foreseeable future for the prevention of clinical malaria in children living in high-risk areas.

Despite these advances in preventative measures, widespread resistance to currently available antimalarial drugs is well documented and is further contributing to the disease burden by limiting available treatments.^6,7^ Particularly troublesome is the recent emergence of partial resistance to artesunate (part of the current frontline treatments for uncomplicated malaria) on the African continent in northern Uganda.^6^ Thus, there is an urgent need for new antimalarial drugs and a well-stocked antimalarial drug discovery pipeline.

Our team previously described the analysis of a high-throughput screen of a Charles River Laboratories proprietary library that identified several new potential chemotypes with activity against Plasmodium falciparum (abbreviated herein as *Pf*).^8^ During the evaluation of active chemotypes, priority was given to cores containing a higher sp^3^ character that would likely have improved physicochemical, pharmacokinetic, and/or toxicological profiles.^9,10^

From this screen, we identified a spiroindoline series (generic structure **1**, Figure 1) that showed good activity against asexual blood stage (ABS) parasites (representative hit compound **2** *Pf* Dd2 IC_50_ = 0.18 μM, Figure 1). The sp^3^ character of the series, combined with the favorable physical chemical properties (clogD_7.4_ = 2.3 and tPSA = 58 Å^2^) and several sites accessible for diversification (Figure 1) added to its appeal. While spirofused compounds exemplified by the ATP4 inhibitor cipargamin^11,12^ have previously been explored, the spiroindoline core, while identified in a previous screen,^13^ has (to our knowledge) not been further evaluated in a drug discovery campaign. Herein, we report the profiling of promising spiroindoline hit **2** along with the hit-to-lead efforts that followed.

**Figure 1:**
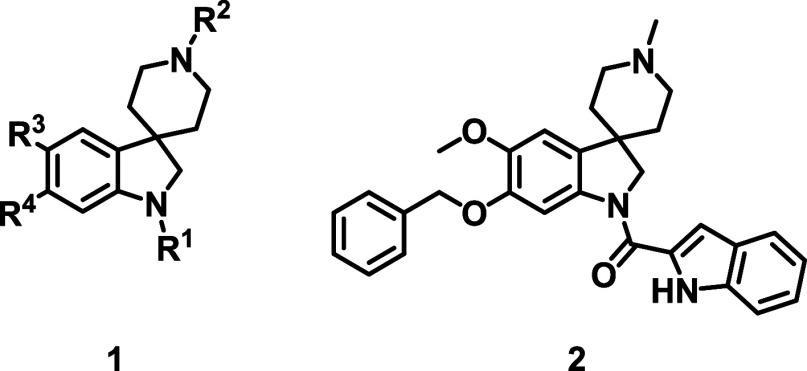
Generic structure of spiroindoline series **1** and hit **2**.

## Results and Discussion

### Chemistry

The general synthetic route to spiroindoline compounds (variable R^1^ substituents) is outlined in Scheme 1, beginning with the benzylation of commercially available 4-fluoro-2-methoxyanisole **3** to afford **4**. Subsequent treatment of **4** with nitric acid afforded the desired nitro compound **5**. Following α-deprotonation of ethyl *N*-Boc-piperidine-4-carboxylate using LDA, a nucleophilic displacement of the fluorine of **5** afforded **6**. The nitro group was reduced with zinc in one pot, with a subsequent cyclization affording lactam **7**. Both the lactam and the carbamate were reduced with LiAlH_4_ to afford the diamine that was then converted to the dihydrochloride salt to afford **8** as the bulk intermediate for most of the compounds described herein. The desired R^1^ substituents could then be installed through amide coupling reactions or through a Buchwald–Hartwig arylation in the case of **50** (Table 7). For the synthesis of compound **11** wherein the piperidine nitrogen is unsubstituted, selective lactam reduction of **7** was achieved by using BH_3_·SMe_2_ to afford intermediate **9**. The indolecarboxamide was then installed using DMC and DIPEA (general amide coupling conditions) to afford **10**, which was *N*-Boc-deprotected in acidic conditions to afford **11**. This methodology was also used for the synthesis of **26** starting with 4-fluoro-1,2-dimethoxybenzene.

**Scheme 1:**
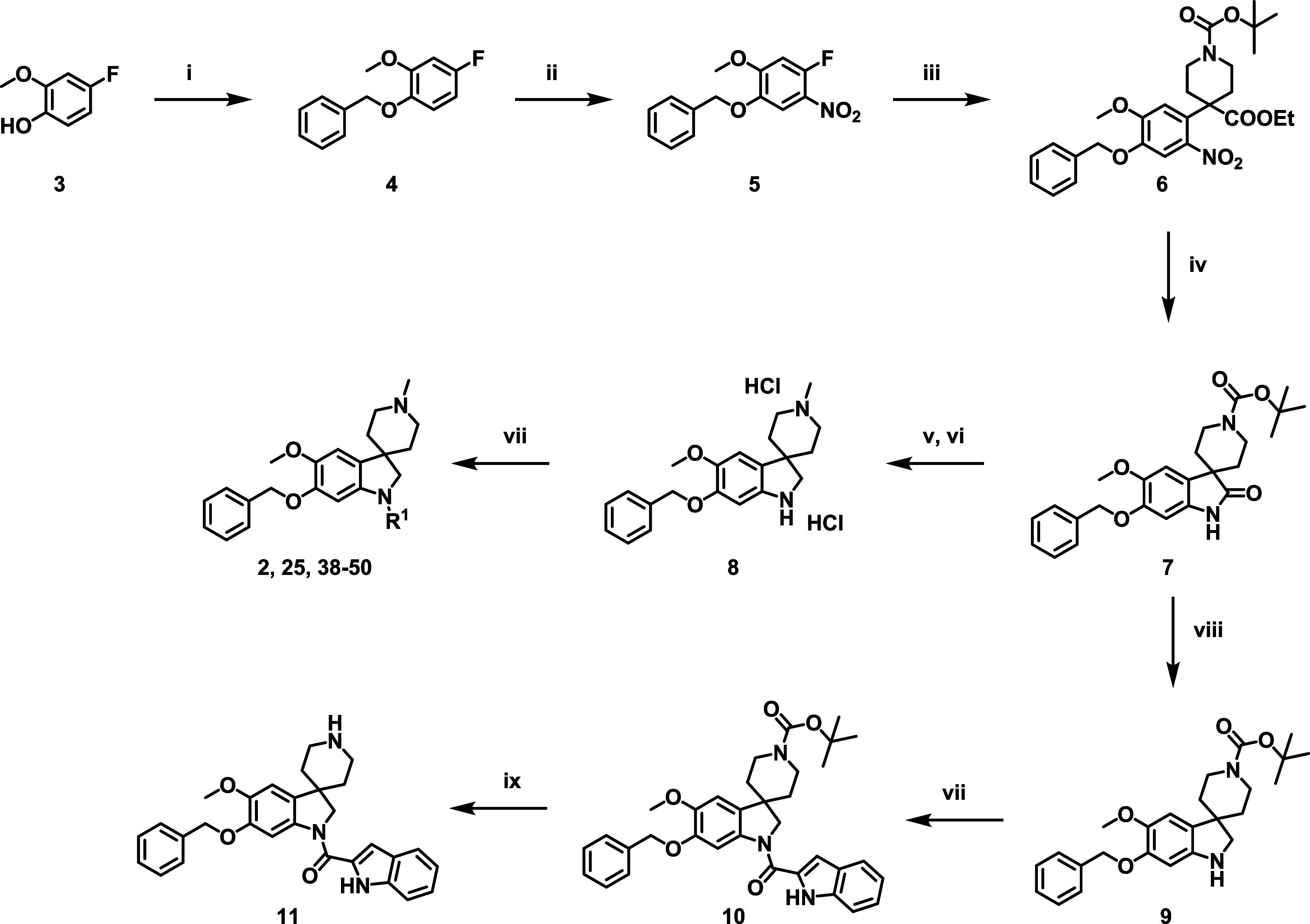
General Route for the Synthesis of Spiroindolines Using LDA^a^

a Conditions: (i) K_2_CO_3_, BnBr, DMF, 16 h, 23 °C, 78%; (ii) HNO_3_, AcOH, 30 min, 0–23 °C, 83%; (iii) ethyl *N*-Boc-piperidine-4-carboxylate, LDA, THF, −78–23 °C, 57%; (iv) Zn, AcOH, 1 h, 60 °C, 36%; (v) LiAlH_4_, THF, 3 h, 70 °C, 74% followed by (vi) HCl (4 M dioxane), Et_2_O, 30 min, 23 °C, 97%; (vii) R^1^ = amides: R^1^CO_2_H, DMC, COMU and DIPEA or EDC and DMAP, DCM, 16 h, 50 °C, 8–79% or R^1^ = 4-pyridyl: 4-chloropyridine, RuPhos, Pd_2_(dba)_3_, K_2_CO_3_, *n*-BuOH, 18 h, 100 °C, 24%; (viii) BH_3_·SMe_2_, THF, 40 h, 70 °C, 45%; and (ix) HCl/dioxane, 16 h, 23 °C, 86%.

The synthesis of **14** was performed as depicted in Scheme 2, involving a Fischer indole reaction between phenylhydrazine and protected piperidine carbaldehyde to afford indoline **12**. The indolecarboxamide R^1^ substituent was installed by using an amide coupling reaction, resulting in compound **13**. Deprotection of the Cbz group with 10% palladium-on-carbon (Pd/C) preceded a reductive amination with formaldehyde to afford *N*-methylpiperidine **14**.

**Scheme 2:**
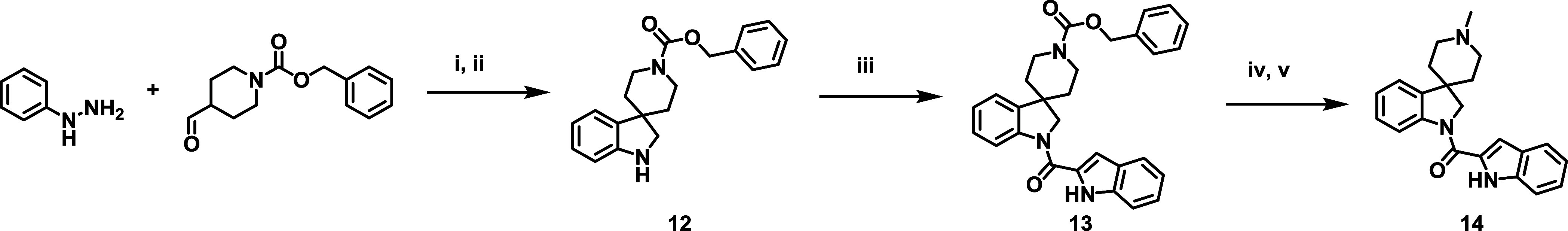
Synthetic Route for the Synthesis of Spiroindoline **14** Using Phenylhydrazine^a^

a Conditions: (i) TFA, toluene/MeCN, 16 h, 35 °C, then (ii) NaBH_4_, 1 h, 0–23 °C, 43%; (iii) indolecarboxylic acid, HATU, DIPEA, 2 h, 60 °C, 62%; (iv) 10% Pd/C, EtOH, 18 h, 23 °C, 86%; and (v) formaldehyde, STAB, DCM, 4 h, 40 °C, 8%.

To explore R^4^ substituents (Figure 1), intermediate **16** was prepared from indolecarboxamide **2** (Scheme 3). Following *N*-Boc protection of the indole **2** to afford **15**, the benzyl group was removed using hydrogen gas and 10% palladium on carbon to afford phenol **16** that was subsequently alkylated or arylated using either standard nucleophilic substitution or Chan–Lam conditions, respectively. Depending on the nature of the R^4^ substituent, the *N*-Boc group was then removed either thermally or through acidic conditions to furnish the desired compounds.

**Scheme 3:**
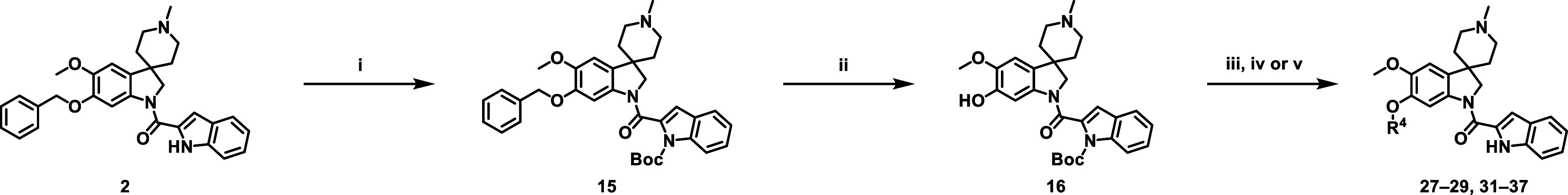
General Synthetic Route for the R^4^ Substituted Analogues^a^

a Conditions: (i) Boc_2_O, DMAP, DCM, 1 h, 23 °C, 83%; (ii) H_2_, 10% Pd/C, THF, 18 h, 23 °C, 94%; (iii) R^4^ = alkyl: RCl or RBr, Cs_2_CO_3_, DMF, 6 h, 60 °C followed by 18 h, 140 °C, 12–68%; (iv) for R^4^ = alkyl: R^4^-Cl or R^4^-Br, Cs_2_CO_3_, DMF, 6 h, 60 °C followed by TFA, DCM, 18 h, 0–23 °C, 52%; and (v) for R^4^ = aryl: R^4^-B­(OH)_2_, pyridine, Cu­(OAc)_2_, DCM, 18 h, 23 °C followed by TFA, DCM, 18 h, 0–23 °C, 51%.

Compound **30** was synthesized from intermediate **7** (Scheme 4) which was debenzylated using standard reductive procedures to afford intermediate **17**, which was converted to triflate **18** using 1,1,1-trifluoro-*N*-phenyl-*N*-((trifluoromethyl)­sulfonyl)­methanesulfonamide. A Suzuki–Miyaura cross-coupling reaction with (*E*)-styrylboronic acid catalyzed with Pd­(dppf)­Cl_2_ afforded styrene **19**, which was subsequently reduced with hydrogen catalyzed by 10% Pd/C to afford lactam **20**. Selective reduction of the lactam was achieved using the 10 M borane-dimethylsulfide complex to afford **21**. The indolecarboxamide R^1^ substituent was installed using standard reductive amination conditions (**22**), and subsequent deprotection with 4 N HCl in EtOAc afforded compound **30** as an HCl salt.

**Scheme 4:**
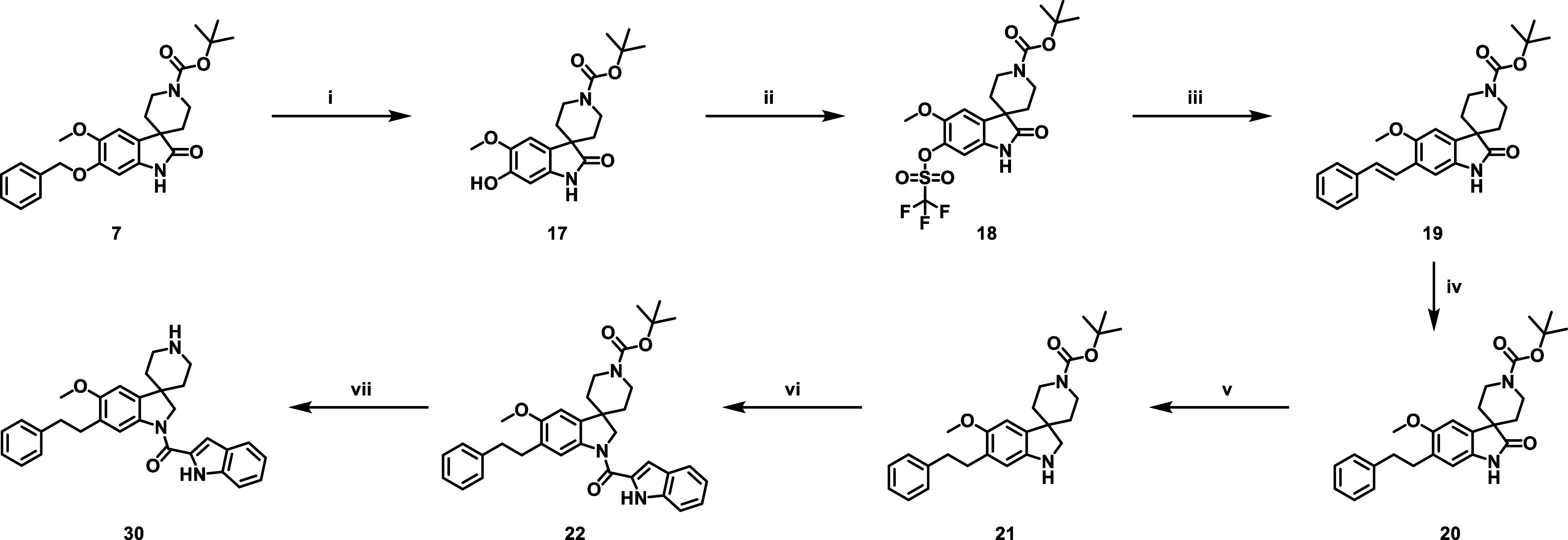
Synthetic Scheme for the Synthesis of **30** ^a^

a Conditions: (i) H_2_, 10% Pd/C, THF, 1 h, 23 °C, 70%; (ii) 1,1,1-trifluoro-*N*-phenyl-*N*-((trifluoromethyl)­sulfonyl)­methanesulfonamide, NEt_3_, DCM, 12 h, 0–23 °C, 97%; (iii) (*E*)-styrylboronic acid, Pd­(dppf)­Cl_2_ and K_2_CO_3_, DMF/water (360/1), 11 h, 90 °C, 43%; (iv) H_2_, 10% Pd/C, THF/MeOH (2.5/1), 12 h, 25 °C, 15 psi, 98%; (v) BH_3_·SMe_2_ (10 M), THF, 18 h, 70 °C, 59%; (vi) 1*H*-indole-2-carboxylic acid, HATU, DMF, DIPEA, 15 h, 25 °C; and (vii) HCl in EtOAc (4 N), EtOAc, 5 h, 25 °C, 70%.

Representative experimental details are provided in the Experimental Section, and synthetic details and compound characterization for all compounds discussed herein are available in the Supporting Information.

### Hit Validation and Profiling

Life cycle profiling of **2** confirmed ABS activity in both P. falciparum chloroquine-sensitive NF54 (IC_50_ = 0.24 μM) and chloroquine-resistant K1 strains (IC_50_ = 0.088 μM) but showed weak activities against the liver stage of rodent malaria parasite Plasmodium berghei (*Pb* sporozoite IC_50_ = 6.8 μM) and mature gametocytes of P. falciparum (*Pf* mature gametocyte IC_50_ = 2.1 μM). Its profile is summarized in Table 1. Compound **2** showed moderate aqueous solubility (30 μM), low cytotoxicity (CHO IC_50_ = 32 μM), and concerning hERG activity (hERG IC_50_ = 2.0 μM). Moderate microsomal stability was observed in rats and mice, but the compound was stable in human liver microsomes (CL_int,app_ *h*/*r*/*m* = < 10.4/55/109 mL/min/kg). From this profile, it became clear that ABS activity, aqueous solubility, and microsomal stability required improvement and that hERG activity needed to be mitigated.

**Table 1:**
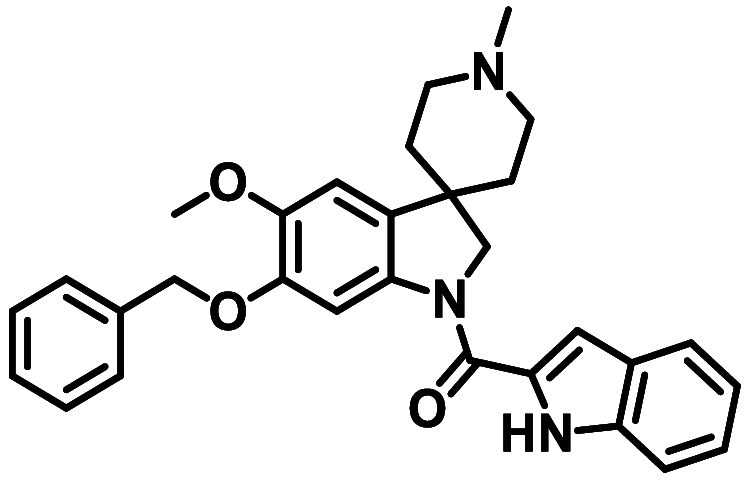
Properties of the Spiroindoline Series Hit **2**

*Pf* NF54 IC_50_ (μM)^a^	0.24
*Pf* K1 IC_50_ (μM)^a^	0.088
*Pb* sporozoite IC_50_ (μM)	6.8
*Pf* mature gametocyte IC_50_ (μM)	2.1
logPRR, lag time (h), PCT_99.9_ (h)	3.8, 24, 58
aqueous solubility PBS pH 6.5 (μM)	30
LM^b^ CL_int,app_ *h*/*r*/*m* (mL/min/kg)	<10.4/55/109
CHO cytotoxicity IC_50_ (μM)	32
hERG IC_50_ (μM)	2.0

a Mean value of two independent experiments with multidrug-sensitive (NF54) strain of P. falciparum using the [^3^H]-hypoxanthine incorporation assay.

b LM refers to liver microsomes; CL_int,app_ is not corrected for microsomal binding.

*In vitro* killing kinetics of compound **2**, investigated through the parasite reduction ratio (PRR) assay at 10 × IC_50_, was moderate with a logPRR of 3.8 and a 24 h lag time, similar to that of pyrimethamine (Figure 2).^14^ No cross-resistance was observed with compound **2** against a panel of field isolates with known antimalarial resistance profiles (Table 2) and resistant mutants generated against antimalarial drug candidates in the lab (Table 3). Testing against lab-generated mutants of drug candidates can help to identify overlaps in mechanisms of action or resistance, e.g., with a high resistance risk, such as DSM265 that targets *Pf*DHODH. It can also help to avoid developing compounds with the same mode of resistance as one that is already in clinical development, such as GNF156 (now known as ganaplacide, currently in phase III). Therefore, this might point to a potentially novel mode of action that makes the series a useful addition to the current portfolio of blood-stage active antiplasmodials.^14^

**Figure 2:**
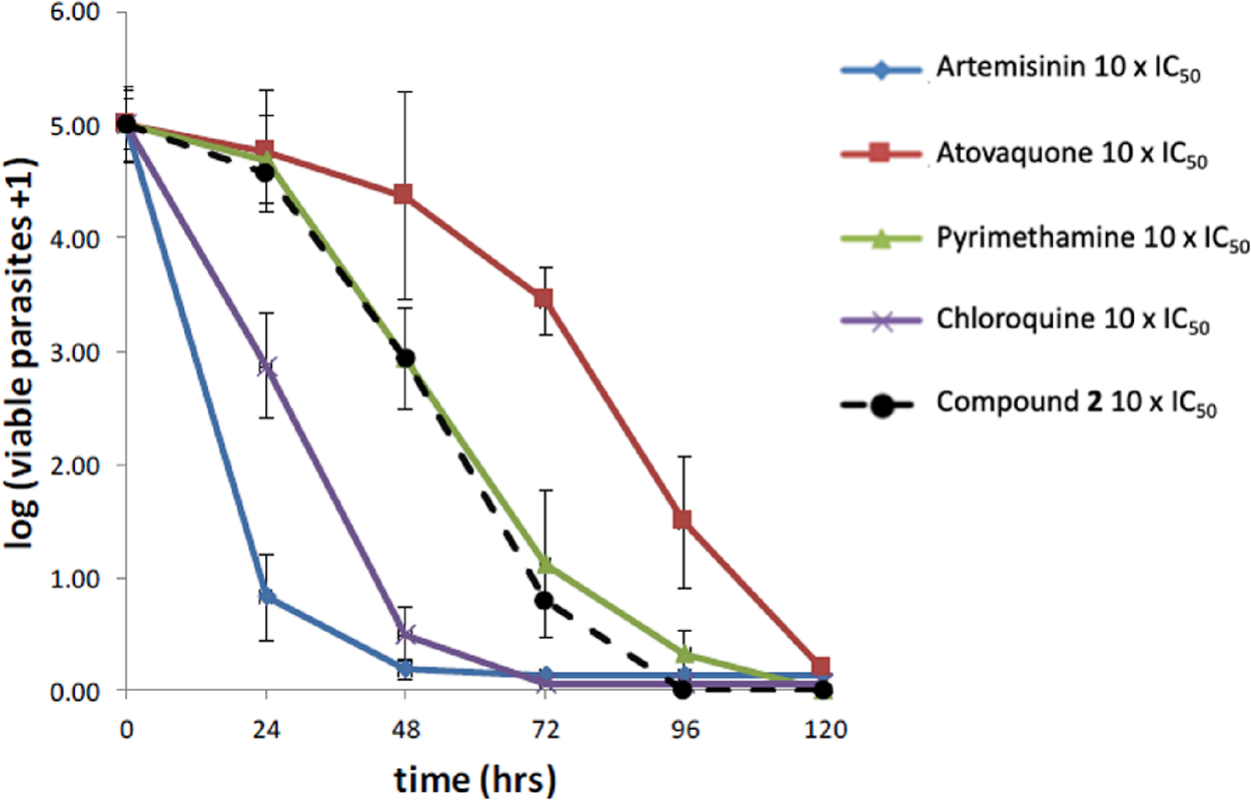
Full parasite reduction ratio (PRR) assay was used to determine the killing kinetics after treatment with compound **2** or standard antimalarial.

**Table 2:**
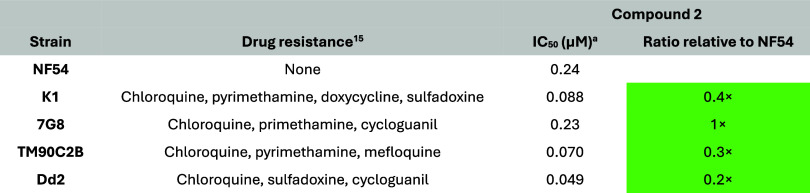
Activity (Mean Values, from At Least Two Independent Biological Replicates) against Field Isolates Using the [^3^H]-Hypoxanthine Incorporation Assay^15^

a The majority of the individual values varied less than 2× (maximum 3×). The parasite strains were obtained from the Biodefense and Emerging Infections Research Resources Repository.

**Table 3:**
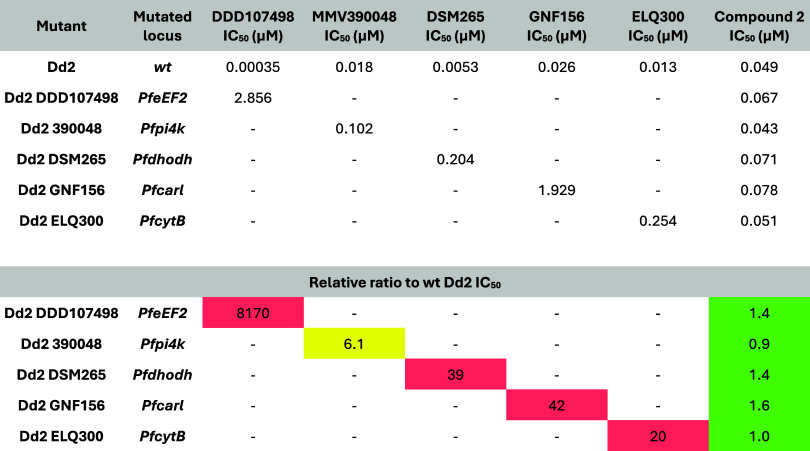
Activity of Compound **2** (Mean Values, from At Least Two Independent Biological Replicates) against Resistant Mutants Generated against Different Antimalarial Drug Candidates in the Lab

The majority of the individual values varied less than 2× (maximum 3×). The [^3^H]-hypoxanthine incorporation assay was used. Criteria for the classification of cross-resistance and hypersensitivity are shown below.

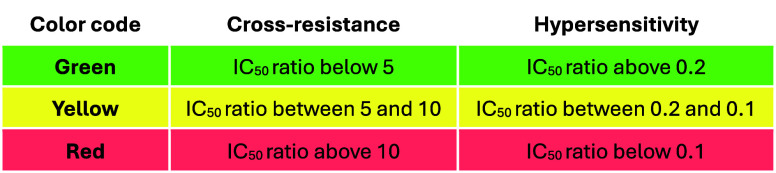

### Minimum Pharmacophore Determination

To determine the features of the hit deemed to be essential for antiplasmodial activity, the initial exploration aimed to determine the minimum pharmacophore. A summary of these efforts is given in Tables 4 and 5. Exploration around R^1^ indicated that the indolecarboxamide functionality was necessary for the activity. Both the reductions of the amide linker to the methylene (**24**) and the pyrrolecarboxamide (**25**) led to a 10–20-fold loss in antiplasmodial activity (*Pf* NF54 IC_50_ = 4.4 and 3.2 μM, respectively), pointing toward the carbonyl and the phenyl being potentially involved in direct interactions with the target(s). Therefore, it was unsurprising that the removal of the R^1^ substituent resulted in a substantial loss of activity (**8**, *Pf* NF54 IC_50_ > 6.0 μM).

**Table 4:**
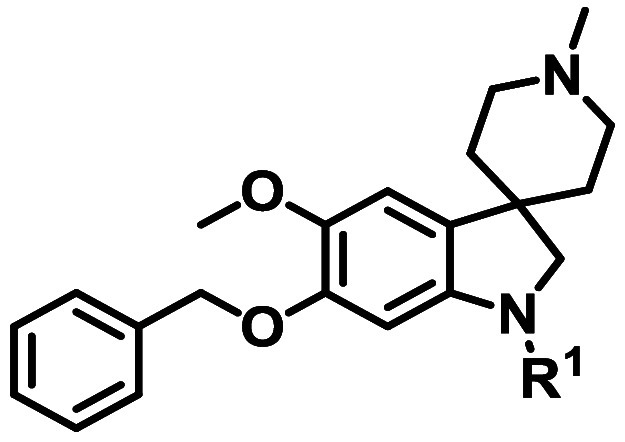

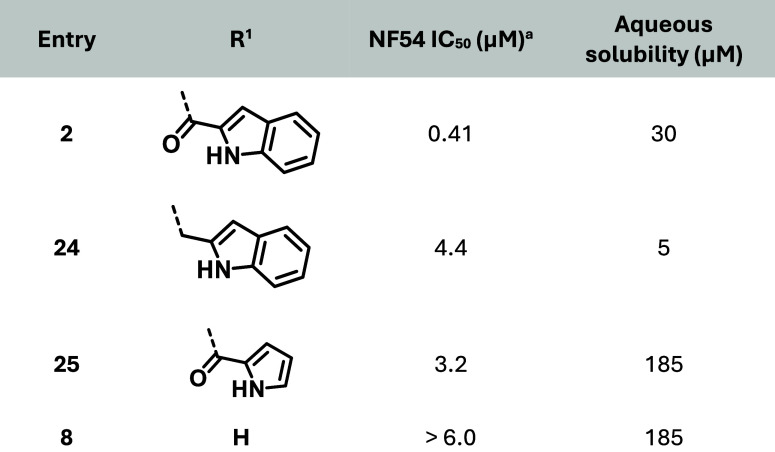
Minimum Pharmacophore Exploration around R^1^

a Mean from three technical replicates of at least two independent experiments with the multidrug-sensitive (NF54) strain of P. falciparum using the parasite lactate dehydrogenase (pLDH) assay. The majority of the individual values varied less than 2× (maximum 3×).

**Table 5:**
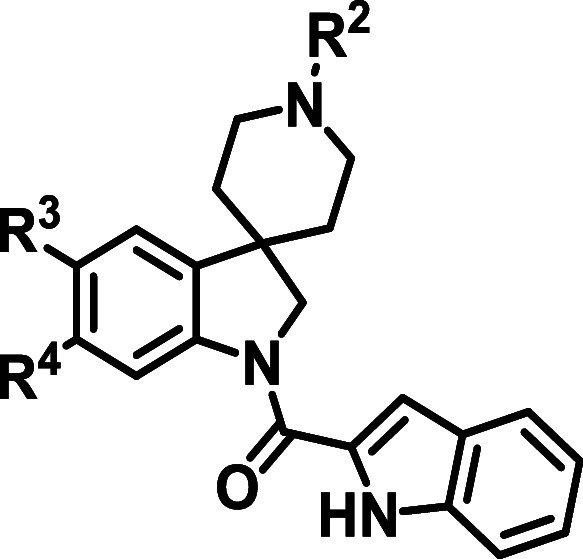
Minimum Pharmacophore Exploration around R^2^, R^3^, and R^4^

**Entry**	**R** ^**2**^	**R** ^**3**^	**R** ^**4**^	**NF54 IC**_**50**_ **(μM)** ^a^	**Aqueous solubility (μM)**	**Cytotoxicity IC**_**50**_ **CHO (μM)** ^b^
**11**	H	OMe	OBn	0.26	90	44
**14**	Me	H	H	5.9	180	
**26**	Me	OMe	OMe	2.5	200	

a Mean from three technical replicates of at least two independent experiments with the multidrug-sensitive (NF54) strain of P. falciparum using the parasite lactate dehydrogenase (pLDH) assay. The majority of the individual values varied less than 2× (maximum 3×).

b Mean of three values performed on one occasion. Individual replicates varied less than 2×.

The unsubstituted piperidine **11** was equipotent (*Pf* NF54 IC_50_ = 0.26 μM) to the *N*-methyl analogue **2** (Table 5). The main metabolic liabilities of **2** in mice were the oxidation of R^1^ and demethylation of R^2^ or R^3^ (Supporting Information). Compound **11** as a putative active *N*-demethylated metabolite of **2** had a higher aqueous solubility of 90 μM and improved microsomal stability (CL_int,app_ *h*/*r*/*m* = <10.4/<20.9/<45.7 mL/min/kg). Its equipotency to compound **2** suggests that it would not be a major concern for efficacy if *N*-demethylation were the main route of metabolism.

Removal of both R^3^ and R^4^ substituents (**14**) led to a significant loss in NF54 activity (IC_50_ = 5.9 μM). Dimethoxy compound **26** showed a 6-fold loss of antiplasmodial activity (*Pf* NF54 IC_50_ = 2.5 μM), suggesting that the phenyl group at R^4^ provided lipophilic contributions to antiplasmodial activity. Therefore, both the R^4^ and R^1^ substituents provided the most substantial contributions to the activity and required optimization to improve antiplasmodial activity.

### 4 SAR Exploration

#### R^4^ SAR Exploration

To improve antiplasmodial activity, a variety of R^4^ substituents were explored (Table 6). Early studies indicated that aromaticity at R^4^ was preferred for activity as compounds with cyclopropyl methyl and propargyl ethers (**27** and **28**) and other alkyl substituents (such as **29**) lost antiplasmodial activity. The linker oxygen could be replaced with carbon (as exemplified by **30** *Pf* NF54 IC_50_ = 0.29 μM), and aryl ethers such as phenyl **31** and phenyl sulfonamide **32** were tolerated (*Pf* NF54 IC_50_ = 0.34 μM for the latter). The presence of the sulfonamide of compound **32** showed that some polarity could be tolerated in this region. However, since these types of substitutions did not improve activity and carried significant metabolic liabilities with high microsomal clearances (e.g., **32** LM CL_int,app_ *h*/*r*/*m* = 155/345/710 mL/min/kg), they were not further pursued. Heterocycles (exemplified by **33**) in place of phenyl improved aqueous solubility but were poorly tolerated (**33** *Pf* NF54 IC_50_ = 2.2 μM). Small substitutions on the benzyl ring were tolerated (such as *p*-methoxy **34** or *p*-fluoro **35**), and substitutions in the *meta* and *ortho* substitutions improved aqueous solubility (as with fluoro-substituted **36** and **37** in which aqueous solubility increased to 135 and 125 μM, respectively).

**Table 6:**
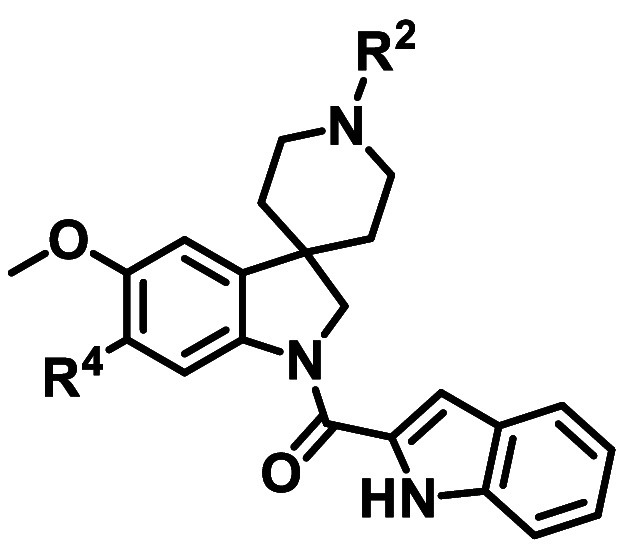

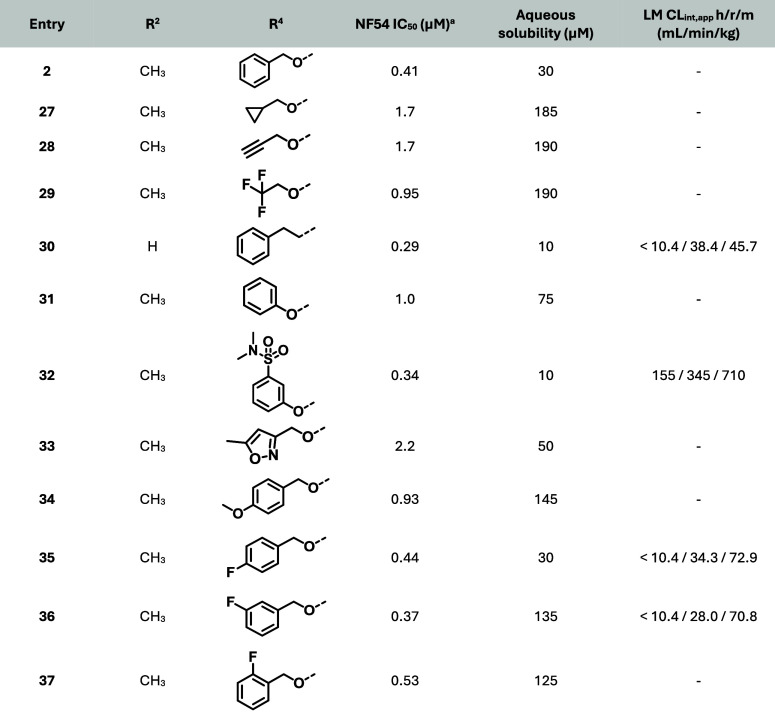
R^4^ SAR and SPR Exploration

a Mean from three technical replicates of two independent experiments with the multidrug-sensitive (NF54) strain of P. falciparum using the lactate dehydrogenase (pLDH) assay. The majority of the individual values varied less than 2× (maximum 3×).

#### R^1^ SAR Exploration

SAR exploration around the indolecarboxamide at R^1^ is shown in Table 7. Introduction of a hydrogen bond acceptor on the five-membered ring of the indole (e.g., the benzimidazole **38**) was detrimental to activity (*Pf* NF54 IC_50_ = 2.1 μM). Compounds where the indole hydrogen bond donor was replaced with a hydrogen bond acceptor (such as benzofuran **39** and imidazopyridine **40**) lost significant activity (*Pf* NF54 IC_50_ = 2.1 and 3.8 μM, respectively), demonstrating that the hydrogen bond donor on the indole is beneficial for antiplasmodial activity. Substituents on the 5-position of the indole (**41** and **42**) slightly improved the NF54 activity and aqueous solubility. The azaindole **43** was the first compound showing substantially improved activity (*Pf* NF54 IC_50_ = 0.13 μM). Despite the increased cytotoxicity, this was the first indication that the NF54 activity could be meaningfully improved.

**Table 7:**
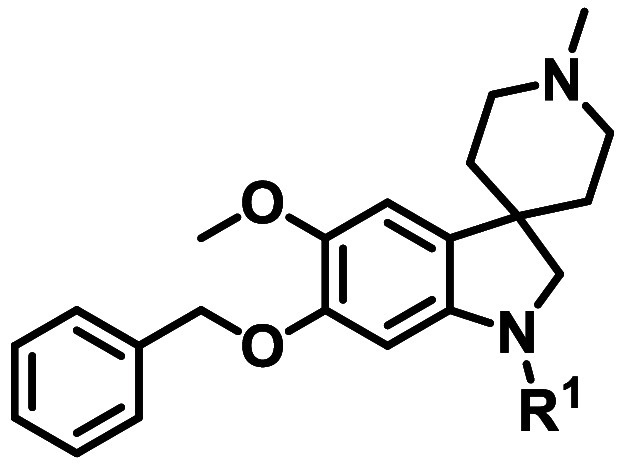

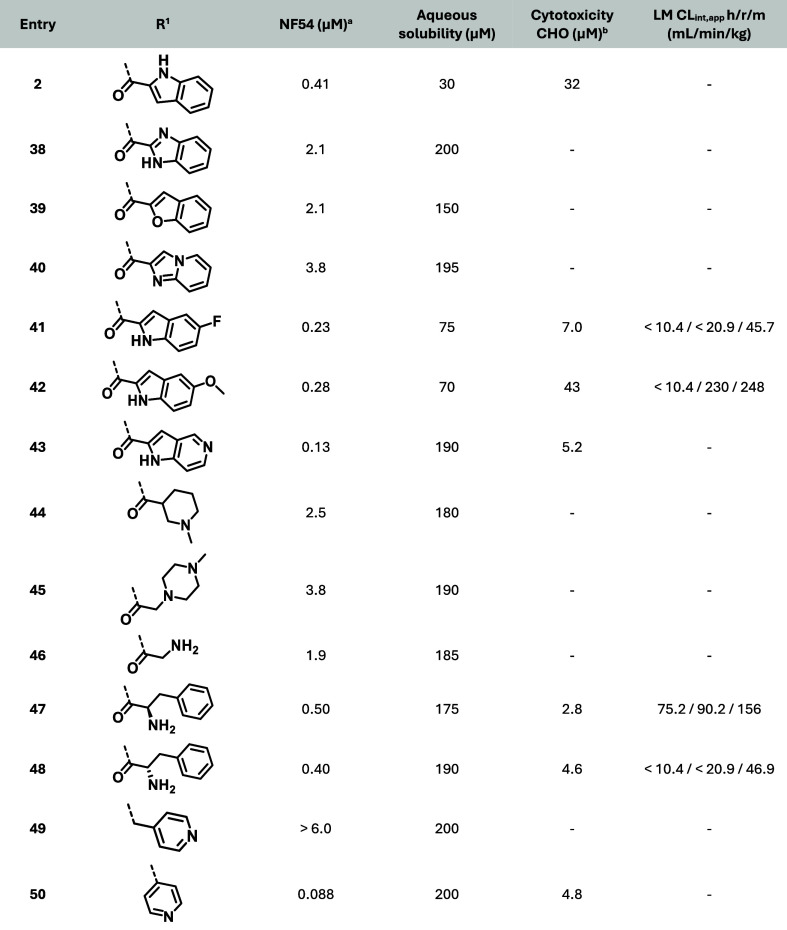
SAR Exploration around R^1^

a Mean from three technical replicates of at least two independent experiments with the multidrug-sensitive (NF54) strain of P. falciparum using the parasite lactate dehydrogenase (pLDH) assay. The majority of the individual values varied less than 2× (maximum 3×).

b Mean of three values performed on one occasion. Individual replicates varied less than 2×.

It was suspected that the basicity of the azaindole (p*K*_a, calc_ = 7.3) was contributing to the increase in activity, and so several other basic groups were investigated. Among those investigated, saturated bases (such as **44** and **45**) showed poor antiplasmodial activity (*Pf* NF54 IC_50_ = 2.5 and 3.5 μM, respectively). The aminoacetyl substituent (as in **46**) showed a 5-fold loss in activity (*Pf* NF54 IC_50_ = 1.9 μM). Interestingly, antiplasmodial activity was recovered with the reintroduction of the phenyl group, as in phenylalanine derivatives (**47** and **48**) that showed antiplasmodial activity and microsomal stability (data available in the SI) comparable to **1** and had significantly improved aqueous solubility. To further explore the contribution of basicity to antiplasmodial activity, the substituent range was further widened to include a variety of other linkers, prompting a wider exploration of basic substituents containing aromatic groups beyond substituted amides. The 4-pyridinemethyl substituent (**49**, p*K*_a, calc_ = 4.8) led to lower activity (*Pf* NF54 IC_50_ > 6 μM) and favorable aqueous solubility. However, the 4-pyridyl substituent (**50**, p*K*_a, calc_ = 7.3) showed notably improved antiplasmodial activity (*Pf* NF54 IC_50_ = 0.080 μM) and, as expected, high aqueous solubility (200 μM). Overall, there was considerable latitude for variation of the R^1^ substituent in terms of activity and aqueous solubility, which offered opportunities for future SAR investigations.

### Parasitological and Life Cycle Profiling

The improved ABS activity and aqueous solubility profile of **43** and **50** prompted further profiling against the liver and sexually transmissible gametocyte stages of the *Plasmodium* parasite to determine their potential for prophylaxis and transmission blocking, respectively. These new frontrunners did not show appreciable activity against the liver stage of the human malaria parasite Plasmodium falciparum (*Pf* NF54 liver schizont IC_50_ = 2.1 and 5.5 μM, respectively). However, this does not negatively impact the development of the series, as it is a treatment against the asexual blood stage cycle that is responsible for attenuating clinical symptoms such as periodical fever. Therefore, asexual blood stage activity is the critical factor. Compound **43** showed late-stage gametocyte activity (*Pf* late-stage gametocyte IC_50_ = 0.12 μM) suggesting the potential for transmission blocking, while compound **50** showed poor activity (*Pf* late-stage gametocyte IC_50_ = 1.4 μM). While this also does not negatively impact further development of the series, transmission-blocking activity would have been beneficial to further limit the spread of the disease.

**Figure 3:**
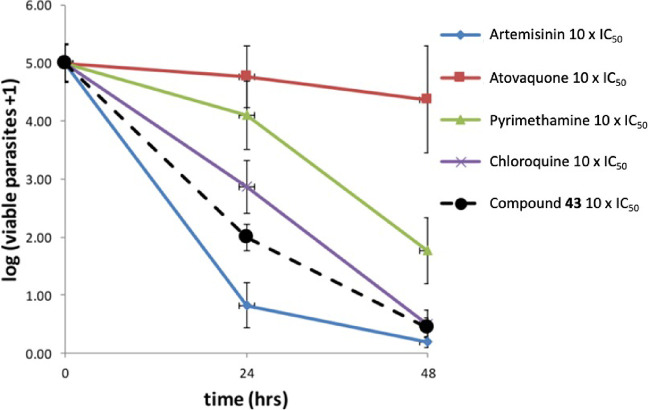
Two-point assay for the estimation of the kill rate for compound **43** in comparison with standard antimalarials. The percentage of surviving parasites after treatment is presented.

Surprisingly, both compounds **43** and **50** were shown to be fast acting in the two-point PRR assay.^14^ This was confirmed when compound **50** showed a killing profile similar to chloroquine (fast acting, logPRR = 4.5, no lag time, PCT_99.9%_ = 32 h, Figure 4) in the full course PRR at 10× IC_50_, contrasting with what was observed in compound **2** (moderate acting, logPRR = 3.8, 24 h lag time, PCT_99.9%_ = 58 h, Figure 2). Compound **43** also showed a similar speed of kill to that of chloroquine (Figure 3). A fast-acting asexual blood stage compound with a very long half-life (see PK data below) has the potential for both treating and preventing malaria, operating as a prophylaxis agent for the latter. Given that the asexual blood stage cycle is responsible for the clinical symptoms of malaria, a fast-killing compound will lead to a quicker relief of symptoms for the patient. The stage specificity assay^16^ revealed better activity for compound **50** against the ring blood stage over the schizont stage, while **2** did not show any preference for any of the asexual blood stage forms (Table S1, Figure S1) after 24 h of incubation.

**Figure 4:**
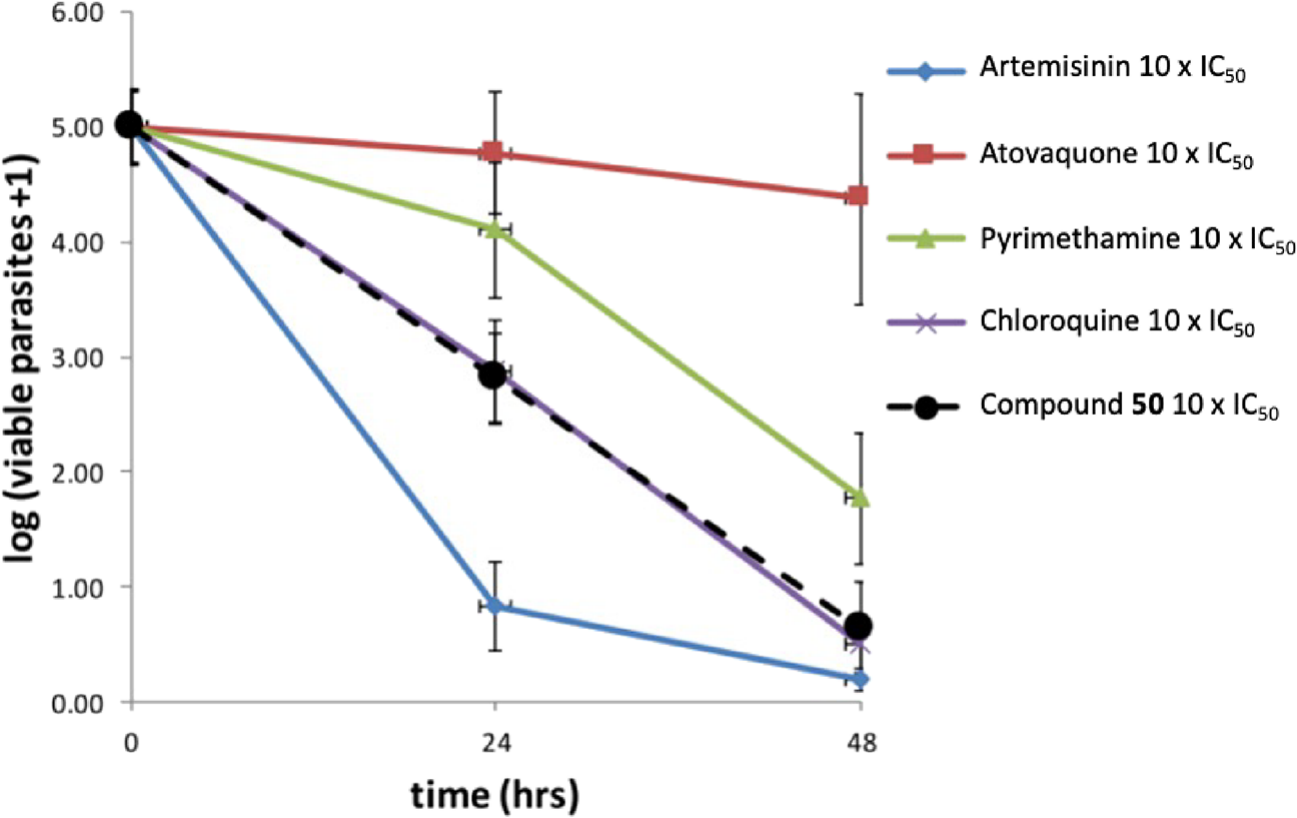
Full parasite reduction ratio (PRR) assay for the determination of killing kinetics after treatment with **50** or standard antimalarials.

**Table 8:**
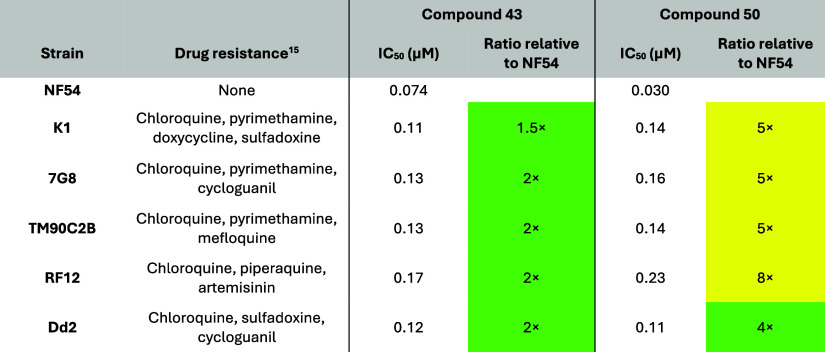
Activity (Mean Values, from At Least Two Independent Biological Replicates) against Field Isolates Using the [^3^H]-Hypoxanthine Incorporation Assay^a^ ^15^

a The majority of the individual values varied less than 2× (maximum 3×). The parasite strains were obtained from BEI Resources.

Compounds **43** and **50** were both profiled against a panel of lab-generated mutants resistant to antimalarials in clinical development and against clinical field isolates with different resistance profiles. Neither compound showed cross-resistance in the lab-generated resistant mutant panel (Table 9), meaning that the modes of resistance to compounds in clinical development do not lead to resistance against compounds **43** and **50**. In the field isolate panel with known multidrug resistance profiles, no cross-resistance was observed with azaindole **43** (Table 8). Slightly elevated ratios with chloroquine-resistant strains (NF54 IC_50_/resistant strain IC_50_ = 4–8×; <5 ideal) were observed with **50** (Table 8) against the same panel. The basic nature of compound **50** along with the elevated cross-resistance margins in chloroquine-resistant cell lines (K1, 7G8, and Dd2) and chloroquine-like speed of kill in the PRR assay suggests that compound **50** could act in a similar manner to chloroquine.

**Table 9:**
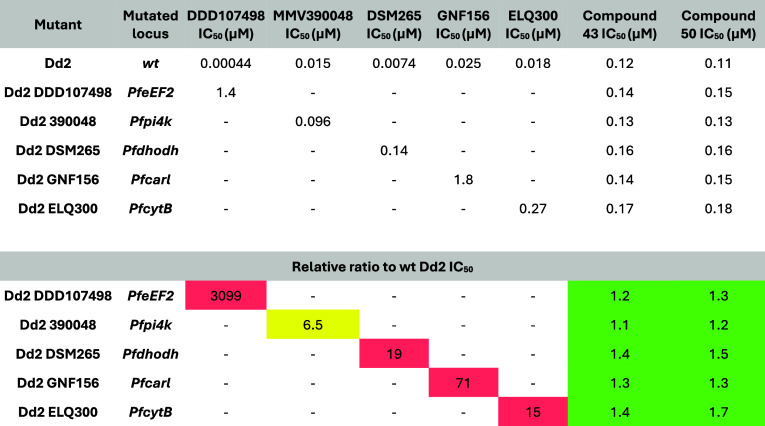
Activity (Mean Values, from At Least Two Independent Biological Replicates) against Lab-Generated Resistant Mutants Using the [^3^H]-Hypoxanthine Incorporation Assay^a^

a The majority of the individual values varied less than 2× (maximum 3×).

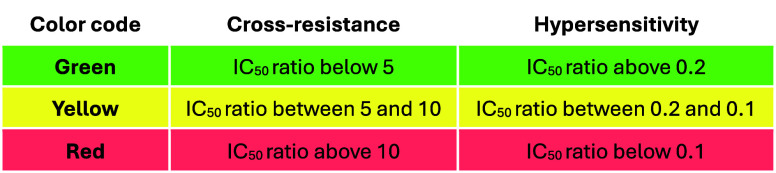

These differences in parasitological profiles (PRR, stage specificity assays, and cross-resistance profiles) suggest that **2** and **50** have potentially different modes-of-action (MOAs). At this point in time, the MOA of these compounds is not known.

### 6 ADMET Profiling

As described in Table 10, **43** and **50** showed improved microsomal metabolic stability over that of **2**. Metabolic liabilities of **2** such as R^1^ oxidation were better addressed by the introduction of a 4-pyridyl functionality (**50**) rather than a 5-azaindole carboxamide (**43**) at R^1^ as shown by the improved microsomal stability of **50** (Table 10). No CYP enzyme inhibition was observed (2C9, 3A4, 2D6, 2C19 IC_50_ > 20 μM) with either frontrunner. Compounds **43** and **50** showed higher cytotoxicity (CHO IC_50_ = 5.1 and 4.8 μM, respectively) relative to that of **2** (CHO IC_50_/*Pf* NF54 IC_50_ SI = 39 and 55, respectively). hERG inhibitory activity was observed for both compounds (hERG IC_50_ = 1.0 and 6.5 μM, respectively). Both cytotoxicity and hERG activity would thus need to be addressed during further optimization work; however, these could be further optimized during the hit-to-lead program.

**Table 10:**
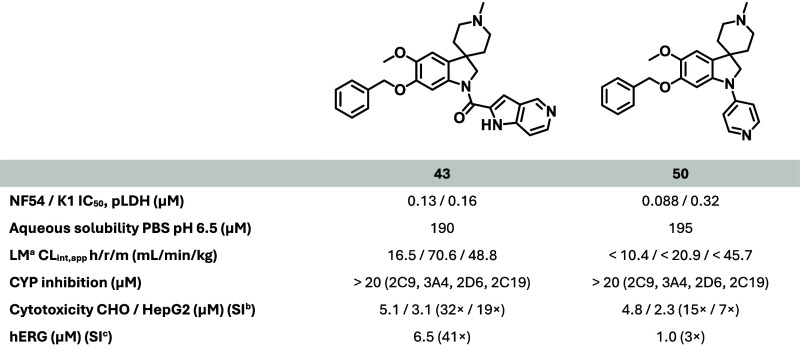
Biological Profiles of Compounds **43** and **50**

a LM refers to liver microsomes.

b SI refers to the selectivity index and is the ratio between the CC_50_ and the IC_50_ of the least susceptible Plasmodial strain.

c SI refers to the selectivity index and is the ratio between the hERG IC_50_ and the IC_50_ of the least susceptible Plasmodial strain.

### 7 *In Vivo* Pharmacokinetics and Efficacy

While both compounds **43** and **50** appear superior to compound **2**, compound **50** showed higher microsomal stability and so was selected for efficacy investigations *in vivo*.

A mouse PK study showed that **50** had high bioavailability (65%), moderate blood clearance (CL_b_ = 29 mL/min/kg), and high steady-state volume of distribution (*V*_ss_ = 25 L/kg), which resulted in a long half-life (>49 h) as shown in Table 11. Due to these favorable PK properties and acceptable cytotoxicity margins relative to NF54 activity, **50** was evaluated for *in vivo* efficacy in an NSG mouse model of P. falciparum infection.

The therapeutic efficacy of **50** was evaluated following a single oral dose of 35, 10, and 4 mg/kg (Figure 5 and Table S7). Overall, *C*_max_ and oral exposures were dose-proportional (Table S7). The variability in oral exposure is likely due to the low aqueous solubility, which was observed in the formulation. While a complete kill was not achieved, an 87% reduction in parasitemia was observed at the highest 35 mg/kg dose compared with the untreated control group, thereby demonstrating an important pharmacological proof-of-concept (PoC) for this novel chemotype as an antiplasmodial agent. Further improvements in efficacy can clearly be envisioned with analogs having higher activity, improved PK properties, and higher aqueous solubility.

**Figure 5:**
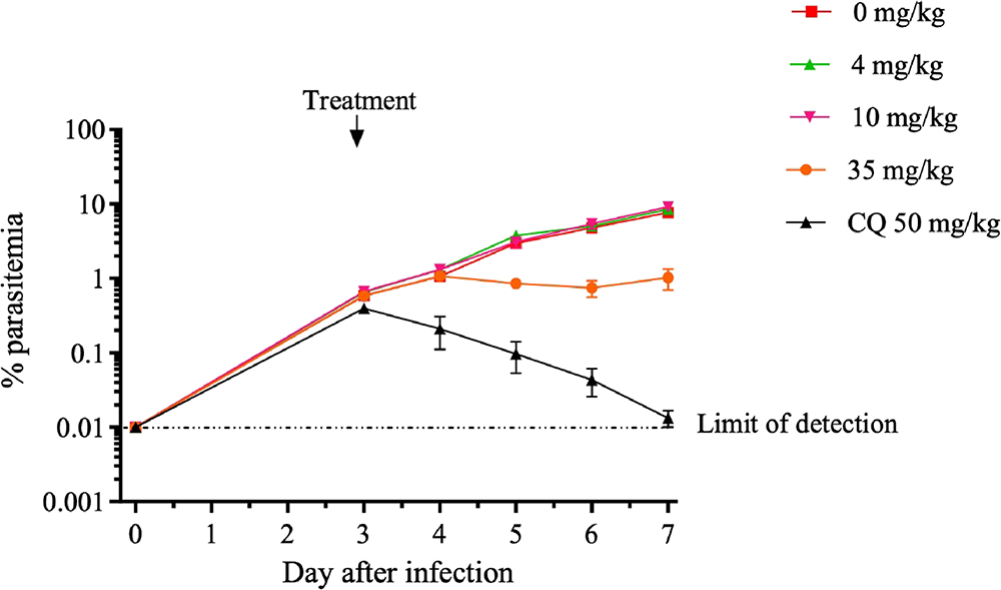
Results of the *in vivo* efficacy study using *P. falciparum*-infected NSG mice in a single-dose regimen of **50**. Percent parasitemia was observed after treatment.

**Table 11:** Mouse Pharmacokinetic Parameters Calculated from Noncompartmental Analysis for Compound **50** ^a^

**route**	**nominal dose**(mg/kg)	*C*_**max**_ **(μM)**	*T*_**max**_ **(h)**	*t*_**1/2**_ **terminal (h)**	**mouse CL**_**b**_(mL/min/kg)	***V***_**ss**_ (L/kg)	**AUC**_**0–24**_ **(min μmol/L)**	***F* (%)**
i.v.	3	0.26		173	28.7	25.0	255	
p.o.	10	0.74	0.5	49			552	64.8

a i.v., intravenous; p.o., oral; CL_b_, blood clearance; *V*_ss_, AUC, area under the concentration–time curve from 0 to 24 h; *F*, oral bioavailability.

## Conclusions

Hit **2** identified from a Charles River Laboratory proprietary library showed potential for development as a novel antimalarial chemotype. An extensive SAR exploration revealed that while some minor changes were tolerated around the molecule, antiplasmodial activity could only be significantly improved by the introduction of the basic 5-azaindole carboxamide or 4-pyridyl functionalities at R^1^. Two compounds, **43** and **50**, emerged as the most promising based on the combination of antiplasmodial activity and PK properties. Although aqueous solubility and microsomal stability were greatly improved compared with **2**, cytotoxicity and hERG margins for compounds **43** and **50** need further improvement. Potential strategies to mitigate hERG activity include decreasing lipophilicity, lowering basicity, or introducing additional groups to induce steric clashes. These structural changes in going from **2** to **50** appear to have introduced a distinct mode-of-action, as evidenced by the increase in the speed of kill (PRR) as well as the missing lag time in the newer compounds, along with differences in stage specificity. Compound **50** showed excellent *in vivo* PK properties, i.e., good bioavailability, high volume of distribution, and long half-life, consistent with the PK properties required for single oral dose cure. Efficacy was achieved in the NSG mouse model with an 87% reduction of parasitemia at a single dose of 35 mg/kg, making **50** an attractive new starting point for the development of a novel antimalarial drug.

## Experimental Section

All commercially available chemicals were purchased from Sigma-Aldrich, Combi-Blocks, or Fluorochem. ^1^H NMR spectra were recorded on a Bruker Spectrometer at 300 MHz. ^13^C NMR spectra were recorded on a Bruker spectrometer at 400 MHz (^1^H 400.2 MHz; ^13^C 100.6 MHz), Bruker-600 (^1^H 600.3 MHz; ^13^C 150.9 MHz), or 600 MHz Varian spectrometer (^1^H MHz; ^13^C MHz). Chemical shifts (δ) are given in parts per million (ppm) referenced to the respective residual solvent peak. Coupling constants, *J*, are recorded in hertz (Hz). Standard acronyms representing multiplicity are used as follows: br s = broad singlet, s = singlet, d = doublet, t = triplet, and m = multiplet. Column chromatography was performed with a Teledyne ISCO CombiFlash RF system using silica or C-18 SiliaSep universal flash cartridges from SiliCycle or a Waters ZQ prep system (column: Waters CSH C18 100 × 30 mm, 5 m; channel A: water + 0.1% TFA, channel B: MeCN + 0.1% TFA; gradient 9.5 min 05–98% B (low pH method)). Purity was determined using an Agilent LC/MC system consisting of an Agilent 1260 Infinity binary pump, Agilent 1260 Infinity standard autosampler, and Agilent 6120 quadrupole (single) mass spectrometer equipped with APCI and ESI multimode ionization source, and all compounds tested for biological activity were confirmed to have ≥95% purity. Any synthetic protocols or data not shown are supplied in the Supporting Information.

### 4-Fluoro-2-methoxy-1-phenylmethoxybenzene (**4**)

4-Fluoro-2-methoxyphenol (70.0 g, 492 mmol) was dissolved in DMF (176 mL) before the addition of K_2_CO_3_ (136 g, 985 mmol, 2 equiv) while stirring. Benzyl bromide (70 mL, 591 mmol, 1.2 equiv) was added, and the reaction was stirred at 23 °C for 18 h. The solid salt precipitate was removed by filtration through a sintered funnel, and the solid was washed with EtOAc. The reaction mixture was then concentrated *in vacuo* and resuspended in EtOAc. NEt_3_ (10 mL) was added to the solution, and the reaction mixture was transferred to a separation funnel where it was washed with H_2_O (3 × 200 mL). The organic layer was dried over anhydrous MgSO_4_, filtered, and concentrated *in vacuo*. The residue was resuspended in EtOAc and washed with 50% brine (4 × 300 mL). The organic layer was dried over anhydrous MgSO_4_, filtered, and concentrated *in vacuo* until a white precipitate formed. *n*-Hexane was added to facilitate precipitation, and the white precipitate was filtered from the solution. The filtrate was concentrated *in vacuo* and resuspended in a small amount of DCM, and the product was triturated with *n*-hexane in an ice bath. The resulting beige powder was collected by filtration and added to the earlier white precipitate, and this solid was found to be the desired 4-fluoro-2-methoxy-1-phenylmethoxybenzene *(* **4** *)*.

Yield: 89 g (78%). ^1^H NMR (300 MHz, C*D*Cl_3_) δ 7.51–7.29 (m, 5H), 6.83 (dd, *J* = 8.9, 5.5 Hz, 1H), 6.68 (dd, *J* = 10.1, 2.9 Hz, 1H), 6.55 (m, 1H), 5.12 (s, 2H), 3.89 (s, 3H).

### 1-Fluoro-5-methoxy-2-nitro-4-phenylmethoxybenzene (**5**)

To ice-cold nitric acid (50 mL, 624 mmol, 14 equiv) stirring rapidly, 4-fluoro-2-methoxy-1-phenylmethoxybenzene (**4**) (10.4 g, 44.8 mmol) dissolved in AcOH (100 mL) was added dropwise. The mixture was stirred at 0 °C for 15 min and then brought to 23 °C for an additional 15 min. The orange solution was diluted with H_2_O, and the resulting pale-yellow solid was filtered and washed with water until the filtrate was pH neutral and then dried to give 1-fluoro-5-methoxy-2-nitro-4-phenylmethoxybenzene (**5**).

Yield: 10.2 g (83%). ^1^H NMR (300 MHz, C*D*Cl_3_) δ 7.67 (d, *J* = 7.2 Hz, 1H), 7.51–7.31 (m, 5H), 6.75 (d, *J* = 12.4 Hz, 1H), 5.18 (s, 2H), 3.98 (s, 3H). LC/MS (ESI^+^) found *m*/*z* = 278.1 [M + H]^+^ (calc for C_14_H_12_FNO_4_ *m*/*z* = 278.1 [M + H]^+^).

### 1-*O*-*tert*-Butyl 4-*O*-Ethyl 4-(5-Methoxy-2-nitro-4-phenylmethoxyphenyl)­piperidine-1,4-dicarboxylate (**6**)

An oven-dried 250 mL two-necked RB flask was fitted with a stirrer bar and dropping funnel and placed under a vacuum. The flask was charged with N_2_ (the recharge process was repeated three times). The LDA solution, 1.0 M in THF/hexanes (31 mL, 30.7 mmol, 1.7 equiv), was added to the flask and dissolved in anhydrous THF (30 mL) under N_2_. The solution was cooled to −78 °C in an acetone/liquid N_2_ bath. Ethyl *N*-Boc-piperidine-4-carboxylate (6.6 mL, 27.1 mmol, 1.5 equiv) was dissolved in anhydrous THF (30 mL) and added dropwise to the rapidly stirring solution at −78 °C; the reaction was left to stir for 1 h at this temperature.

1-Fluoro-5-methoxy-2-nitro-4-phenylmethoxybenzene **5** (5.00 g, 18.0 mmol) was dissolved in anhydrous THF (30 mL), transferred to the dropping funnel, and added to the cold solution dropwise. The reaction was then allowed to gradually warm from −78 to 23 °C over 1.5 h. Sat. NH_4_Cl solution (100 mL) was added to quench the reaction and the reaction mixture. The reaction mixture was transferred to a separation funnel and diluted with EtOAc (50 mL). The organic layer was then washed with brine (100 mL), dried over anhydrous Na_2_SO_4_, filtered, and concentrated *in vacuo*. The resulting orange residue was purified by using a Teledyne ISCO CombiFlash system, eluting a gradient of 0–50% EtOAc in *n*-hexane. The fractions containing the product were combined and concentrated *in vacuo*, and 1-*O*-*tert*-butyl 4-*O*-ethyl 4-(5-methoxy-2-nitro-4-phenylmethoxyphenyl)­piperidine-1,4-dicarboxylate, **6**, was collected as a dark orange oil and used without further purification.

Yield: 7.02 g (57%). ^1^H NMR (300 MHz, C*D*Cl_3_) δ 7.48 (s, 1H), 7.32 (m, 5H), 6.93 (s, 1H), 5.08 (s, 2H), 4.08 (q, *J* = 7.1 Hz, 2H), 3.91 (s, 3H), 3.50 (s, 4H), 2.37–2.17 (m, 2H), 2.05–1.71 (m, 2H), 1.38 (s, 9H), 1.27–1.07 (m, 3H). LC/MS (ESI^+^) found *m*/*z* = 415.1 [M + H-*Boc*]^+^ (calc for C_22_H_26_N_2_O_6_ *m*/*z* = 415.2 [M + H-*Boc*]^+^).

### *tert*-Butyl 5-Methoxy-2-oxo-6-phenylmethoxyspiro­[1*H*-indole-3,4′-piperidine]-1′-carboxylate­(**7**)

Zinc powder (11.0 g) was weighed into a 250 mL RB flask. A 2% aqueous HCl solution (50 mL) was added to the flask and stirred for 5 min at 23 °C. The solution was decanted, and a further portion of 2% aqueous HCl (50 mL) solution was added. After stirring for 5 min, the solution was decanted. H_2_O (100 mL) was added to the flask and decanted (×2). AcOH (20 mL) was then added to the flask, and the stirring solution gradually warmed to 50 °C. 1-*O*-*tert*-Butyl 4-*O*-ethyl 4-(5-methoxy-2-nitro-4-phenylmethoxyphenyl)­piperidine-1,4-dicarboxylate **6** (8.97 g, 16.2 mmol) was dissolved in the remaining acetic acid (70 mL) and added dropwise to the warmed solution. The temperature of the solution was raised to 60 °C, and the reaction was stirred for 2 h. After cooling, the zinc and zinc acetate precipitate was removed by filtration through a bed of Celite, and the filter cake was washed thoroughly with DCM. The filtrate was then transferred to a separation funnel and diluted with H_2_O (200 mL) and DCM (150 mL). The organic layer was extracted and washed with a further portion of H_2_O before drying over anhydrous Na_2_SO_4_, filtration and concentration *in vacuo*. EtOAc (50 mL) was added to the resulting dark purple gum, and the solution was subjected to sonication for 5–10 min until a precipitate appeared. The solution was then left to stand for 30 min before the pale pink precipitate was collected by filtration and determined to be the desired *tert*-butyl 5-methoxy-2-oxo-6-phenylmethoxyspiro­[1*H*-indole-3,4′-piperidine]-1′-carboxylate (**7**).

Yield: 2.84 g (36%). ^1^H NMR (300 MHz, C*D*Cl_3_) δ 7.75 (s, 1H), 7.51–7.31 (m, 5H), 6.89 (s, 1H), 6.55 (s, 1H), 5.16 (s, 2H), 3.89 (s, 3H), 3.87–3.71 (m, 4H), 1.85 (m, 4H), 1.53 (s, 9H). LC/MS (ESI^–^) found *m*/*z* = 437.2 [M – H]^−^ (calc for C_25_H_30_N_2_O_5_ *m*/*z* = 437.2 [M – H]^−^).

### 5-Methoxy-1′-methyl-6-phenylmethoxyspiro­[1,2-dihydroindole-3,4′-piperidine] Dihydrochloride (**8**)

Step 1: *tert*-Butyl 5-methoxy-2-oxo-6-phenylmethoxyspiro­[1*H*-indole-3,4′-piperidine]-1′-carboxylate **7** (3.44 g, 7.84 mmol) was added to a clean, dry two-neck flask fitted with a condenser and stirrer bar, and the reaction vessel was placed under a dry N_2_ atmosphere. The contents were then dissolved in anhydrous THF (44 mL), and LiAlH_4_ (1.49 g, 39.2 mmol, 5 equiv) was added slowly portionwise at 23 °C. The reaction mixture was then heated to 70 °C for 3 h. After cooling to 23 °C, H_2_O was added dropwise until all of the LiAlH_4_ had been successfully quenched. The reaction mixture was then filtered through a bed of Celite, the filter cake was washed with EtOAc, and the filtrate was transferred to a separation funnel. The mixture was further diluted with EtOAc (20 mL) and washed with H_2_O (2 × 50 mL) by a brine wash (50 mL). The organic layer was then dried over anhydrous Na_2_SO_4_, filtered, and concentrated *in vacuo*.

^1^H NMR (300 MHz, C*D*Cl_3_) δ 7.32–7.24 (m, 2H), 7.24–7.09 (m, 3H), 6.58 (s, 1H), 6.17 (s, 1H), 4.93 (s, 2H), 3.66 (s, 3H), 3.23 (s, 2H), 2.69 (dt, *J* = 11.7, 3.4 Hz, 2H), 2.19 (s, 3H), 1.92 (td, *J* = 11.8, 2.2 Hz, 2H), 1.79 (td, *J* = 12.7, 3.8 Hz, 2H), 1.56 (m, 2H). LC/MS (ESI^+^) found *m*/*z* = 339.2 [M + H]^+^ (calc for C_21_H_26_N_2_O_2_ *m*/*z* = 339.2 [M + H]^+^). Purity by LC (280 nm): 100%.

Step 2: The yellow oil was then suspended in DCM (30 mL) under a N_2_ atmosphere, Et_2_O (2 mL) was added, and HCl in dioxane 4 M solution (7.84 mL, 31.4 mmol, 4 equiv) was slowly dropped into the rapidly stirring solution. A pale green precipitate resulted. The precipitate was collected by filtration and washed with further portions of Et_2_O. The pale green solid was dried to afford the desired product, 5-methoxy-1′-methyl-6-phenylmethoxyspiro­[1,2-dihydroindole-3,4′-piperidine] dihydrochloride (**8**), which was collected as a pale brown powder.

Yield: 3.20 g (97%). ^1^H NMR (300 MHz, DMSO-*d*_6_) δ 10.59 (s, 1H), 7.26–7.08 (m, 5H), 6.92 (s, 1H), 6.61 (s, 1H), 4.87 (s, 2H), 3.59 (s, 3H), 3.49 (s, 2H), 3.19 (d, *J* = 12.5 Hz, 2H), 2.91 (q, *J* = 12.1 Hz, 2H), 2.53 (d, *J* = 4.3 Hz, 3H), 2.07 (t, *J* = 13.2 Hz, 2H), 1.72 (d, *J* = 14.1 Hz, 2H). LC/MS (ESI^+^) found *m*/*z* = 339.2 [M + H]^+^ (calc for C_21_H_26_N_2_O_2_ *m*/*z* = 339.2 [M + H]^+^).

#### General Amide Coupling Method A

5-Methoxy-1′-methyl-6-phenylmethoxyspiro­[1,2-dihydroindole-3,4′-piperidine] dihydrochloride **8**, carboxylic acid (1.2 equiv), and DMC (1.1 equiv) were weighed into a microwave tube and dissolved in DCM (0.05 M). DIPEA (5 equiv) was then added, and the reaction tube was sealed and heated at 50 °C for 16 h. After cooling to 23 °C, the reaction was quenched by the addition of H_2_O, stirred rapidly for 5 min, and then poured through a phase separation cartridge. After the collected DCM wash was concentrated *in vacuo*, the residue was purified on a Teledyne ISCO CombiFlash system eluting a gradient of 0–20% MeOH in DCM. Fractions found to contain product were combined, concentrated, and further dried on a freeze-dryer for 18 h.

Representative product synthesized using this method: 1*H*-indol-2-yl-(5-methoxy-1′-methyl-6-phenylmethoxyspiro­[2*H*-indole-3,4′-piperidine]-1-yl)­methanone­(**2**)

5-Methoxy-1′-methyl-6-phenylmethoxyspiro­[1,2-dihydroindole-3,4′-piperidine] dihydrochloride **8**, 1*H*-indole-2-carboxylic acid, DIPEA, and DMC were combined using amide coupling method A. Following purification and drying, 1*H*-indol-2-yl-(5-methoxy-1′-methyl-6-phenylmethoxyspiro­[2*H*-indole-3,4′-piperidine]-1-yl)­methanone (**2**) was afforded as a white solid.

Yield: 89 mg (78%). ^1^H NMR (300 MHz, C*D*Cl_3_) δ 9.54 (s, 1H), 8.15 (s, 1H), 7.77 (d, *J* = 8.0 Hz, 1H), 7.56–7.44 (m, 3H), 7.42–7.30 (m, 4H), 7.20 (m, 1H), 7.02 (dd, *J* = 2.1, 0.9 Hz, 1H), 6.83 (d, *J* = 1.4 Hz, 1H), 5.20 (s, 2H), 4.35 (s, 2H), 3.90 (d, *J* = 1.4 Hz, 3H), 2.97 (d, *J* = 8.5 Hz, 2H), 2.42 (s, 3H), 2.25–2.03 (m, 4H), 1.76 (d, *J* = 11.0 Hz, 2H). LC/MS (ESI^+^) found *m*/*z* = 482.2 [M + H]^+^ (calc for C_30_H_31_N_3_O_3_ *m*/*z* = 482.3 [M + H]^+^). Purity by LC (280 nm): 100%.

#### General Amide Coupling Method B

5-Methoxy-1′-methyl-6-phenylmethoxyspiro­[1,2-dihydroindole-3,4′-piperidine] dihydrochloride **8**, carboxylic acid (1.5 equiv), and COMU (1.5 equiv) were weighed into a microwave tube and suspended in DCM (0.05 M). DIPEA (5 equiv) was added, and the tube was sealed and heated to 50 °C for 16 h. On cooling, H_2_O was added to the microwave tube, and the contents were stirred rapidly for 5 min. The suspension was then poured into a phase separation cartridge and washed with DCM. The collected DCM solution was evaporated, and the resulting residue was suspended in DMSO (1 mL) and purified using preparative chromatography. Fractions containing the product were concentrated, and the residue was taken up into a small amount of MeOH. The solution was then loaded onto a preconditioned SCX-2 cartridge and washed with MeOH, and the free base was eluted with 0.5 M NH_3_ in MeOH. The basic wash was concentrated *in vacuo* and further dried on the freeze-dryer for 18 h to afford the desired product.

Compounds containing a Boc protection group were resuspended in DCM and cooled in an ice bath to 0 °C, and TFA (15 equiv) was added. The deprotection was monitored by LCMS, and on completion, the reaction was concentrated *in vacuo*, taken up in MeOH, and loaded onto a preconditioned SCX-2 cartridge. The cartridge was washed with MeOH and 0.5 M NH_3_ in MeOH and used to elute the desired product as the free base.

Representative example synthesized using method B: 1*H*-benzimidazol-2-yl-(5-methoxy-1′-methyl-6-phenylmethoxyspiro­[2*H*-indole-3,4′-piperidine]-1-yl)­methanone (**38**).

5-Methoxy-1′-methyl-6-phenylmethoxyspiro­[1,2-dihydroindole-3,4′-piperidine] dihydrochloride, 1*H*-benzimidazo-2-carboxylic acid, DIPEA, and COMU were combined using amide coupling method B. Following purification and drying, 1*H*-benzimidazol-2-yl-(5-methoxy-1′-methyl-6-phenylmethoxyspiro­[2*H*-indole-3,4′-piperidine]-1-yl)­methanone (**38**) was afforded as a yellow solid.

Yield: 25 mg (52%). ^1^H NMR (600 MHz, C*D*Cl_3_) δ 10.98 (s, 1H), 8.23 (s, 1H), 7.91 (s, 1H), 7.59–7.53 (m, 1H), 7.45 (d, *J* = 7.5 Hz, 2H), 7.35 (q, *J* = 8.4, 7.6 Hz, 4H), 7.28 (d, *J* = 7.4 Hz, 1H), 6.85 (s, 1H), 5.17 (s, 2H), 4.86 (s, 2H), 3.89 (s, 3H), 3.02 (s, 2H), 2.48 (s, 3H), 2.35 (s, 2H), 2.17 (s, 2H), 1.77 (d, *J* = 13.4 Hz, 2H). LC/MS (ESI^+^) found *m*/*z* = 483.2 [M + H]^+^ (calc for C_29_H_30_N_4_O_3_ *m*/*z* = 483.2 [M + H]^+^). Purity by LC (254 nm): 100%.

#### General Amide Coupling Method C

To a microwave tube containing a suspension of 5-methoxy-1′-methyl-6-phenylmethoxyspiro­[1,2-dihydroindole-3,4′-piperidine] dihydrochloride **8** (1 equiv), EDC·HCl (3 equiv), and carboxylic acid (3 equiv) in DCM (0.1 M), DMAP (4 equiv) was added and stirred at 23 °C for 18 h. Following the complete consumption of the starting material the following morning, the reaction was quenched by the addition of H_2_O and further diluted by the addition of a further portion of DCM. The reaction mixture was passed through a phase separation cartridge. The cartridge was washed with further portions of DCM (×3), and the combined organic washes were concentrated *in vacuo*. The resulting residue was then taken up into DMSO (1 mL) and purified by preparative HPLC. Fractions found to contain the product were concentrated, and the residue was resuspended in MeOH and loaded onto a preconditioned SCX-2 cartridge. The cartridge was washed with MeOH followed by 0.5 M NH_3_ in MeOH to elute the compound. The desired products were isolated after final concentration *in vacuo*.

Representative example synthesized using method C: 1-(5-methoxy-1′-methyl-6-phenylmethoxyspiro­[2*H*-indole-3,4′-piperidine]-1-yl)-2-(4-methylpiperazin-1-yl)­ethenone (**45**).

5-Methoxy-1′-methyl-6-phenylmethoxyspiro­[1,2-dihydroindole-3,4′-piperidine] dihydrochloride, 2-(4-methylpiperazin-1-yl)­acetic acid, EDC·HCl, and DMAP were combined using amide coupling method C. Following purification and drying, 1-(5-methoxy-1′-methyl-6-phenylmethoxyspiro­[2*H*-indole-3,4′-piperidine]-1-yl)-2-(4-methylpiperazin-1-yl)­ethenone (**45**) was afforded as an off-white solid.

Yield: 29 mg (61%). ^1^H NMR (600 MHz, methanol-*d*_4_) δ 7.97 (s, 1H), 7.47–7.43 (m, 2H), 7.35 (t, *J* = 7.6 Hz, 2H), 7.29 (t, *J* = 7.4 Hz, 1H), 6.92 (s, 1H), 5.10 (s, 2H), 4.08 (s, 2H), 3.86 (s, 3H), 3.38 (s, 2H), 3.00 (d, *J* = 12.0 Hz, 2H), 2.63 (br m, 8H), 2.46 (s, 3H), 2.36 (s, 5H), 2.02 (td, *J* = 13.5, 4.1 Hz, 2H), 1.70 (d, *J* = 13.6 Hz, 2H). LC/MS (ESI^+^) found *m*/*z* = 479.3 [M + H]^+^ (calc for C_28_H_38_N_4_O_3_ *m*/*z* = 479.3 [M + H]^+^). Purity by LC (254 nm): 100%.

### 5-Methoxy-1′-methyl-6-phenylmethoxy-1-pyridin-4-ylspiro­[2*H*-indole-3,4′-piperidine] (**50**)

A microwave tube containing 5-methoxy-1′-methyl-6-phenylmethoxyspiro­[1,2-dihydroindole-3,4′-piperidine] (400 mg, 1.18 mmol), RuPhos (22.0 mg, 0.0473 mmol, 0.04 equiv), K_2_CO_3_ (490 mg, 3.55 mmol, 3 equiv), Pd_2_(dba)_3_ (130 mg, 0.142 mmol, 0.12 equiv), and 4-chloropyridine hydrochloride (355 mg, 2.36 mmol, 2 equiv) and a stirrer bar was sealed and purged with N_2_. *n*-BuOH (10 mL, 0.12 M) was injected into the tube, and the solution was degassed. The sealed tube was then heated to 100 °C for 18 h. On cooling, the reaction mixture was diluted with EtOAc and H_2_O added to quench the reaction, and the solution was transferred to a separation funnel. The mixture was further diluted with EtOAc and H_2_O. The organic layer was collected and dried over anhydrous MgSO_4_, filtered, and concentrated. The resulting residue was purified on a Teledyne ISCO CombiFlash system eluting a reverse phase solvent gradient of 0.1% formic acid/MeCN in 0.1% formic acid/H_2_O. Fractions containing the product were concentrated, and the residue was taken up in MeOH. The solution was loaded onto a preconditioned SCX-2 cartridge and washed with MeOH, and the product was eluted using 0.5 M NH_3_ in MeOH. The basic solution was further concentrated to afford the desired 5-methoxy-1′-methyl-6-phenylmethoxy-1-pyridin-4-ylspiro­[2*H*-indole-3,4′-piperidine] (**50**) as an off-white powder.

Yield: 117 mg (24%). ^1^H NMR (300 MHz, DMSO-*d*_6_) δ 8.26 (d, *J* = 5.7 Hz, 2H), 7.50–7.29 (m, 5H), 6.98 (dd, *J* = 7.6, 2.6 Hz, 4H), 5.14 (s, 2H), 3.79 (s, 2H), 3.77 (s, 3H), 2.80–2.69 (m, 2H), 2.23 (s, 3H), 2.06 (t, *J* = 11.9 Hz, 2H), 1.91 (td, *J* = 13.9, 13.1, 3.8 Hz, 2H), 1.51 (d, *J* = 12.6 Hz, 2H). LC/MS (ESI^+^) found *m*/*z* = 416.2 [M + H]^+^ (calc for C_26_H_29_N_3_O_2_ *m*/*z* = 416.2 [M + H]^+^). Purity by LC (280 nm): 100%.

### 1*H*-Indol-2-yl-(5-methoxy-6-phenylmethoxyspiro­[2*H*-indole-3,4′-piperidine]-1-yl)­methanone (**11**)

Step 1: Into a solution of *tert*-butyl 6-benzyloxy-5-methoxy-2-oxo-spiro­[indoline-3,4′-piperidine]-1′-carboxylate **7** (15.0 g, 34.2 mmol) in anhydrous THF (150 mL) at 25 °C under an atmosphere of N_2_ was added a ∼10 M solution of borane dimethyl sulfide complex (13.7 mL, ∼137 mmol, 4.0 equiv). The reaction was heated at 70 °C for 40 h, cooled to 25 °C, and then quenched by the slow addition of H_2_O (50 mL). An aqueous 2 M solution of NaOH (50 mL) was added into the solution, and the mixture was stirred for 2 h and then extracted with EtOAc (100 mL × 3). The combined organic phases were twice washed with brine, dried over anhydrous Na_2_SO_4_, filtered, and then concentrated under reduced pressure to obtain the crude that was purified by column chromatography using 25–50% EtOAc in *n*-hexanes followed by recrystallization from EtOAc and *n*-hexanes to obtain *tert*-butyl 6-(benzyloxy)-5-methoxyspiro­[indoline-3,4′-piperidine]-1′-carboxylate (**9**) as an off-white solid.

Yield: 6.50 g (45%). ^1^H NMR (400 MHz, DMSO-*d*_6_): δ 7.42–7.29 (m, 5H), 6.75 (s, 1H), 6.28 (s, 1H), 5.13 (s, 1H), 4.99 (s, 2H), 3.90–3.87 (m, 2H), 3.67 (s, 3H), 3.29 (s, 2H), 2.85 (br s, 2H), 1.67–1.59 (m, 2H), 1.53–1.43 (m, 2H), 1.42 (s, 9H). ^1^H NMR (400 MHz, DMSO-*d*_6_ containing few drops of D_2_O): δ 7.40–7.29 (m, 5H), 6.69 (s, 1H), 6.31 (s, 1H), 4.95 (s, 2H), 3.85 (d, *J* = 12.68 Hz, 2H), 3.63 (s, 3H), 3.25 (s, 2H), 2.82 (br s, 2H), 1.62–1.56 (m, 2H), 1.52–1.47 (m, 2H), 1.38 (s, 9H). LC/MS (ESI^+^) found *m*/*z* = 425 [M + H]^+^ (calc for C_25_H_32_N_2_O_4_ *m*/*z* = 425 [M + H]^+^).

Step 2: Following general amide coupling method A, indole-2-carboxamide was used to afford *ter*t-butyl 1-(1*H*-indole-2-carbonyl)-5-methoxy-6-phenylmethoxyspiro­[2*H*-indole-3,4′-piperidine]-1′-carboxylate (**10**) as a colorless glass.

Yield: 149 mg (95%). LC/MS (ESI^+^) found *m*/*z* = 568.2 [M + H]^+^ (calc for C_34_H_37_N_3_O_5_ *m*/*z* = 568.3 [M + H]^+^).

Step 3: *tert*-Butyl 1-(1*H*-indole-2-carbonyl)-5-methoxy-6-phenylmethoxyspiro­[2*H*-indole-3,4′-piperidine]-1′-carboxylate **10** (149 mg, 0.26 mmol) was taken up in DCM (5 mL). TFA (0.3 mL, 3.94 mmol, 15 equiv) was added dropwise to the stirring solution, and the reaction was left to mature at 23 °C for 20 h. LCMS the following day indicated complete deprotection, and the reaction was quenched by removal of the volatiles *in vacuo*. The resulting residue was adsorbed onto Celite and purified on a Teledyne ISCO CombiFlash system, eluting a gradient of 0–20% MeOH in DCM on a 12 g silica column. Fractions containing the product were combined, concentrated *in vacuo*, and further dried on the freeze-dryer for 18 h to afford the desired 1*H*-indol-2-yl-(5-methoxy-6-phenylmethoxyspiro­[2*H*-indole-3,4′-piperidine]-1-yl)­methanone (**11**) as a white solid.

Yield: 106 mg (86%); ^1^H NMR (400 MHz, DMSO-*d*_6_) δ 11.70 (s, 1H), 8.95–8.67 (br s, 1H), 8.14–7.98 (m, 1H), 7.69 (d, *J* = 8.0 Hz, 1H), 7.47 (dd, *J* = 13.8, 7.9 Hz, 3H), 7.40 (t, *J* = 7.3 Hz, 2H), 7.34 (d, *J* = 7.6 Hz, 2H), 7.24 (t, *J* = 7.6 Hz, 1H), 7.08 (t, *J* = 7.5 Hz, 1H), 6.87 (s, 1H), 5.09 (s, 2H), 4.46 (s, 2H), 3.81 (s, 3H), 3.21 (t, *J* = 12.9 Hz, 2H), 2.10 (td, *J* = 14.0, 4.5 Hz, 2H), 1.84 (d, *J* = 13.8 Hz, 2H). LC/MS (ESI^+^) found *m*/*z* = 468.2 [M + H]^+^ (calc for C_29_H_29_N_3_O_3_ *m*/*z* = 468.2 [M + H]^+^). Purity by LC (280 nm): 100%.

### 1*H*-Indol-2-yl-(1′-methylspiro­[2*H*-indole-3,4′-piperidine]-1-yl)­methanone (**14**)

Step 1: To a solution of phenylhydrazine (0.144 g, 1.33 mmol, 1.1 equiv) in a 98:2 mixture of toluene/MeCN (9.8:0.2 mL, 0.24 M) was added TFA (0.46 mL, 6.07 mmol, 5 equiv), and the mixture was stirred at 35 °C. To this reaction mixture was added dropwise a solution of 4-formyl-*N*-Cbz-piperidine (0.300 g, 1.21 mmol, 1 equiv) in a mixture of toluene/acetonitrile (49:1 mL, 1.2 M). The resulting reaction mixture was stirred at 35 °C for 16 h. The reaction mixture was cooled in an ice–water bath, and MeOH (0.5 mL) was added followed by the addition of NaBH_4_ (0.064 g, 1.70 mmol, 1.4 equiv) portionwise. The resulting reaction mixture was stirred at 23 °C for 1 h. Thereafter, the reaction was diluted with EtOAc and washed with 5% NH_3_ solution and brine, dried over anhydrous Na_2_SO_4_, and filtered, and the solvent was evaporated *in vacuo* to yield a residue. The obtained residue was purified using a Teledyne ISCO CombiFlash system, eluting a gradient of 0–30% EtOAc in *n*-hexanes. Fractions containing the product were combined and concentrated *in vacuo* to afford a brown oil that was found to be the desired benzyl spiro­[1,2-dihydroindole-3,4′-piperidine]-1′-carboxylate.

Yield: 360 mg (43%). LC/MS (ESI^+^) found *m*/*z* = 323.2 [M + H]^+^ (calc for C_20_H_22_N_2_O_2_ *m*/*z* = 323.2 [M + H]^+^).

Step 2: To a stirring solution of indole-2-carboxylic acid (0.216 g, 1.34 mmol, 1.2 equiv) in DCM (10 mL) was added DMC (0.227 g, 1.34 mmol, 1.2 equiv) and DIPEA (0.58 mL, 3.35 mmol, 3 equiv), and the reaction was stirred for 5 min. To this was added the substituted spiro­[1,2-dihydroindole-3,4′-piperidine]-1′-carboxylate (0.360 g, 1.12 mmol, 1 equiv), and the reaction was stirred at 45 °C for 16 h. The reaction mixture was cooled to 23 °C, diluted with H_2_O, and washed with DCM. The combined organic layers were washed with brine, dried over anhydrous Na_2_SO_4_, and filtered, and the solvent was removed *in vacuo* to yield an oily residue that was slurried in MeOH to afford the desired indole as a beige precipitate. This precipitate was used without further purification in the next step.

Yield: 249 mg (62%). LC/MS (ESI^+^) found *m*/*z* = 466.1 [M + H]^+^ (calc for C_29_H_27_N_3_O_3_ *m*/*z* = 466.2 [M + H]^+^).

Step 3: To a stirring solution of spiropiperidine **13** in EtOH (40 mL, 0.01 M) was added 10% palladium on carbon (5.14 mg, 0.05 mmol, 0.1 equiv), and the system was placed under a hydrogen atmosphere. The reaction was stirred at 23 °C for 18 h. The palladium was filtered off through a Celite pad, and the solvent was removed *in vacuo* to yield a yellow gum, which was used in the next step without further purification.

#### 1*H*-Indol-2-yl­(spiro­[2*H*-indole-3,4′-piperidine]-1-yl)­methanone

Yield: 153 mg (86%). LC/MS (ESI^+^) found *m*/*z* = 332.1 [M + H]^+^ (calc for C_21_H_21_N_3_O *m*/*z* = 332.1 [M + H]^+^).

Step 4: To a stirring solution of the precipitate in DCE (10 mL, 0.03 M) was added paraformaldehyde (18.1 mg, 0.600 mmol, 2 equiv), and the mixture was stirred for 5 min. NaBH­(OAc)_3_ (0.128 g, 0.600 mmol, 2 equiv) was added to the reaction, and the mixture was stirred at 40 °C for 5 min. The reaction was diluted with DCM and washed with H_2_O. The organic layer was dried over anhydrous Na_2_SO_4_ and filtered, and the solvent was removed *in vacuo* to yield a brown oil. This was purified on a Teledyne ISCO CombiFlash system, eluting a reverse phase solvent gradient of methanol in 0.1% formic acid/water on a C18 column. Fractions containing the product were combined and concentrated and further purified using a Waters Prep HPLC. Clean fractions were combined, and the solvent was removed on the lyophilizer to yield a fluffy white solid that was found to be the desired 1*H*-indol-2-yl-(1′-methylspiro­[2*H*-indole-3,4′-piperidine]-1-yl)­methanone*.*

Yield: 8.1 mg (8%). ^1^H NMR (300 MHz, Methanol-*d*_4_) δ 8.12 (d, *J* = 8.3 Hz, 1H), 7.70 (d, *J* = 8.1 Hz, 1H), 7.48 (d, *J* = 8.4 Hz, 1H), 7.35–7.20 (m, 4H), 7.13 (dt, *J* = 15.4, 7.3 Hz, 2H), 4.50 (s, 2H), 3.37 (m, 2H), 2.99 (t, *J* = 12.9 Hz, 2H), 2.78 (s, 3H), 2.17 (t, *J* = 13.0 Hz, 2H), 1.97 (d, *J* = 14.6 Hz, 2H). LC/MS (ESI^+^) found *m*/*z* = 346.2 [M + H]^+^ (calc for C_22_H_23_N_3_O *m*/*z* = 346.2 [M + H]^+^). Purity by LC (280 nm): 100%.

### *tert*-Butyl 2-(5-Methoxy-1′-methyl-6-phenylmethoxyspiro­[2*H*-indole-3,4′-piperidine]-1-carbonyl)­indole-1-carboxylate (**15**)

1*H*-Indol-2-yl-(5-methoxy-1′-methyl-6-phenylmethoxyspiro­[2*H*-indole-3,4′-piperidine]-1-yl)­methanone **1** (1.08 g, 2.25 mmol) was dissolved in THF (20 mL). DMAP (302 mg, 2.47 mmol, 1.1 equiv) was added, and the reaction mixture was stirred at 23 °C for 1 h. Boc_2_O (981 mg, 4.50 mmol, 2 equiv) was then added, and the reaction was left to stir for 1 h. The reaction was quenched by the addition of H_2_O, and the reaction mixture was transferred to a separation funnel and washed with further portions of EtOAc and H_2_O. The organic layer was collected and dried over anhydrous Na_2_SO_4_, filtered, and concentrated *in vacuo*. The resulting residue was then adsorbed onto Celite and purified on a Teledyne ISCO CombiFlash system eluting a gradient of 0–20% MeOH in DCM. After concentration of the combined fractions containing the product, *tert*-butyl 2-(5-methoxy-1′-methyl-6-phenylmethoxyspiro­[2*H*-indole-3,4′-piperidine]-1-carbonyl)­indole-1-carboxylate (**15**) was collected as a brown gum.

Yield: 1.09 g (83%). ^1^H NMR (300 MHz, methanol-*d*_4_) δ 8.16 (dd, *J* = 20.0, 8.3 Hz, 1H), 8.02 (s, 1H), 7.69 (dd, *J* = 30.1, 7.8 Hz, 1H), 7.53–7.24 (m, 6H), 7.24–7.15 (m, 1H), 7.01–6.86 (m, 2H), 5.23 (d, *J* = 42.2 Hz, 2H), 4.14 (s, 1H), 3.88 (d, *J* = 6.0 Hz, 3H), 3.81 (s, 1H), 2.89 (dd, *J* = 39.4, 9.6 Hz, 2H), 2.23 (s, 3H), 1.98 (d, *J* = 8.9 Hz, 4H), 1.68 (d, *J* = 10.2 Hz, 2H), 1.42 (d, *J* = 10.8 Hz, 9H). LC/MS (ESI^+^) found *m*/*z* = 582.5 [M + H]^+^ (calc for C_35_H_39_N_3_O_5_ *m*/*z* = 582.3 [M + H]^+^).

### *tert*-Butyl 2-(6-Hydroxy-5-methoxy-1′-methylspiro­[2*H*-indole-3,4′-piperidine]-1-carbonyl)­indole-1-carboxylate (**16**)

*tert*-Butyl 2-(5-methoxy-1′-methyl-6-phenylmethoxyspiro­[2*H*-indole-3,4′-piperidine]-1-carbonyl)­indole-1-carboxylate **15** (561 mg, 0.960 mmol) and 10% Pd/C (64.1 mg, 0.600 mmol) were weighed into a 50 mL RB flask, and the flask was evacuated. Carefully, anhydrous THF (0.2 M) was added to the evacuated flask and then flushed with H_2_ (from a H_2_ balloon) bubbled through the solution to replace the atmosphere, and the reaction was left to proceed at 23 °C for 18 h. The Pd/C was then removed by filtration through a Celite pad, and the filter cake was washed with EtOAc. The concentration of the filtrate afforded an off-white solid that was determined to be the desired product, *tert*-butyl 2-(6-hydroxy-5-methoxy-1′-methylspiro­[2*H*-indole-3,4′-piperidine]-1-carbonyl)­indole-1-carboxylate (**16**), in sufficient purity to be used without further purification in subsequent reactions.

Yield: 446 mg (94%). ^1^H NMR (300 MHz, DMSO-*d*_6_) δ 9.02 (s, 1H), 8.11 (d, *J* = 8.2 Hz, 1H), 7.74 (s, 1H), 7.69 (d, *J* = 7.8 Hz, 1H), 7.42 (t, *J* = 7.7 Hz, 1H), 7.31 (t, *J* = 7.5 Hz, 1H), 7.03 (s, 1H), 6.89 (s, 1H), 3.82 (s, 2H), 3.77 (s, 3H), 2.68 (d, *J* = 10.7 Hz, 2H), 2.10 (s, 3H), 1.94–1.74 (m, 4H), 1.53 (d, *J* = 12.0 Hz, 2H), 1.44 (s, 9H). LC/MS (ESI^+^) found *m*/*z* = 492.2 [M + H]^+^ (calc for C_28_H_33_N_3_O_5_ *m*/*z* = 492.2 [M + H]^+^).

#### General Procedure for the One-Pot SN_2_ Displacement and Boc Deprotection at R^6^

*tert*-Butyl 2-(6-hydroxy-5-methoxy-1′-methylspiro­[2*H*-indole-3,4′-piperidine]-1-carbonyl)­indole-1-carboxylate **16** and Cs_2_CO_3_ (2.5 equiv) were weighed into a microwave tube and suspended in DMF (0.1 M). Alkyl halide (1.1 equiv) was added, and the tube was sealed and heated to 60 °C for 6 h. The temperature was then raised to 140 °C and heated for 18 h. On cooling to 23 °C, it was concentrated, and the residue was resuspended in DCM and H_2_O. After rapid stirring for 5 min, the suspension was passed through a phase separation cartridge, and the collected organic wash was concentrated. The resulting residue was purified on a Teledyne ISCO CombiFlash system, eluting with a reverse phase solvent gradient of 0.1% formic acid/MeCN in 0.1% formic acid/H_2_O. Fractions found to contain the desired product were concentrated *in vacuo*. The free base was obtained by taking up the concentrated residue in MeOH and loading it onto a preconditioned SCX-2 cartridge. After washing the cartridge with MeOH, the product was eluted using 0.5 M NH_3_ in MeOH. Once this wash was concentrated, the final compound was subjected to a final drying on a freeze-dryer.

Representative example using this procedure: [6-(cyclopropylmethoxy)-5-methoxy-1′-methylspiro­[2*H*-indole-3,4′-piperidine]-1-yl]-(1*H*-indol-2-yl)­methanone (**27**).

Combining *tert*-butyl 2-(6-hydroxy-5-methoxy-1′-methylspiro­[2*H*-indole-3,4′-piperidine]-1-carbonyl)­indole-1-carboxylate, Cs_2_CO_3_, and (bromomethyl)­cyclopropane following the one-pot method described above furnished the desired [6-(cyclopropylmethoxy)-5-methoxy-1′-methylspiro­[2*H*-indole-3,4′-piperidine]-1-yl]-(1*H*-indol-2-yl)­methanone (**27**) as a white powder.

Yield: 21 mg (23%). ^1^H NMR (400 MHz, Methanol-*d*_4_) δ 7.82 (br s, 1H), 7.70 (t, *J* = 6.5 Hz, 1H), 7.52–7.45 (m, 1H), 7.30–7.23 (m, 1H), 7.16–7.07 (m, 2H), 6.95–6.90 (m, 1H), 4.37 (d, *J* = 4.0 Hz, 2H), 3.91–3.84 (m, 3H), 3.81 (s, 2H), 3.02–2.90 (m, 2H), 2.46–2.38 (m, 3H), 2.30 (t, *J* = 12.7 Hz, 2H), 2.02 (td, *J* = 13.5, 4.5 Hz, 2H), 1.74 (d, *J* = 13.7 Hz, 2H), 1.28 (s, 1H), 0.59 (d, *J* = 8.2 Hz, 2H), 0.32 (s, 2H). LC/MS (ESI^+^) found *m*/*z* = 446.2 [M + H]^+^ (calc for C_27_H_31_N_3_O_3_ *m*/*z* = 446.3 [M + H]^+^). Purity by LC (280 nm): 100%.

#### General Procedure for the Two-Step SN^2^ Displacement and Boc Removal at R^6^

*tert*-Butyl 2-(6-hydroxy-5-methoxy-1′-methylspiro­[2*H*-indole-3,4′-piperidine]-1-carbonyl)­indole-1-carboxylate **16** and Cs_2_CO_3_ (2.5 equiv) were weighed into a microwave tube and suspended in DMF (0.1 M). Aryl halide (1.1 equiv) was added, and the tube was sealed and heated to 60 °C for 18 h. After this time, the reaction mixture was concentrated, and the residue was resuspended in DCM and quenched with H_2_O. After rapid stirring for 5 min, the suspension was passed through a phase separation cartridge. The collected organic wash was cooled in an ice bath, and TFA (30 equiv) was added slowly. The reaction mixture was allowed to warm to 23 °C for 18 h. The solution was concentrated and purified using a Teledyne ISCO CombiFlash system, eluting a reverse phase solvent gradient of 0.1% formic acid/MeCN in 0.1% formic acid/H_2_O. Fractions found to contain the desired product were concentrated *in vacuo*. The free base was obtained by taking up the concentrated residue in MeOH and loading it onto a preconditioned SCX-2 cartridge. After washing the cartridge with MeOH, the product was eluted using 0.5 M NH_3_ in MeOH. Once this wash was concentrated, the final compound was subjected to a final drying on a freeze-dryer.

Representative example using this procedure: 1*H*-indol-2-yl-[5-methoxy-1′-methyl-6-[(5-methyl-1,2-oxazol-3-yl)­methoxy]­spiro­[2H-indole-3,4′-piperidine]-1-yl]­methanone (**33**).

Combining *tert*-butyl 2-(6-hydroxy-5-methoxy-1′-methylspiro­[2*H*-indole-3,4′-piperidine]-1-carbonyl)­indole-1-carboxylate **16**, Cs_2_CO_3_, and aryl halide following the two-step method described above furnished the desired 1*H*-indol-2-yl-[5-methoxy-1′-methyl-6-[(5-methyl-1,2-oxazol-3-yl)­methoxy]­spiro­[2*H*-indole-3,4′-piperidine]-1-yl]­methanone (**33**) as a white powder.

Yield: 51 mg (52%). ^1^H NMR (300 MHz, Methanol-*d*_4_) δ 7.93 (s, 1H), 7.69 (dt, *J* = 8.0, 1.0 Hz, 1H), 7.48 (dq, *J* = 8.3, 1.0 Hz, 1H), 7.26 (m, 1H), 7.15–7.04 (m, 2H), 6.96 (s, 1H), 6.25 (s, 1H), 5.09 (s, 2H), 4.36 (s, 2H), 3.86 (s, 3H), 2.92 (d, *J* = 12.0 Hz, 2H), 2.41 (d, *J* = 0.9 Hz, 3H), 2.38 (s, 3H), 2.31–2.18 (m, 2H), 2.01 (td, *J* = 13.3, 4.0 Hz, 2H), 1.78–1.66 (m, 2H). LC/MS (ESI^+^) found *m*/*z* = 487.2 [M + H]^+^ (calc for C_28_H_30_N_4_O_4_ *m*/*z* = 487.2 [M + H]^+^). Purity by LC (280 nm): 100%.

#### General Procedure for Chan–Lam Reactions at R^6^

Step 1: *tert*-Butyl 2-(6-hydroxy-5-methoxy-1′-methylspiro­[2*H*-indole-3,4′-piperidine]-1-carbonyl)­indole-1-carboxylate **16**, pyridine (6 equiv), and boronic acid (2.5 equiv) were weighed into a flask and dissolved in DCM (0.1 M). Cu­(OAc)_2_ (0.2 equiv) was added, and the reaction was left to stir open at 23 °C for 16 h. The reaction mixture was diluted with H_2_O and DCM and stirred rapidly for 5 min before being passed through a phase separation cartridge. The collected DCM wash was concentrated *in vacuo* and purified on a Teledyne ISCO CombiFlash system, eluting a gradient of 0–20% MeOH in DCM. Fractions found to contain the product were combined, concentrated *in vacuo*, and used as is in the next reaction.

Step 2: The residue was taken up in DCM (0.03 M) and cooled in an ice bath to 0 °C. TFA (30 equiv) was added slowly, and the reaction was allowed to warm to 23 °C over 18 h. Once LCMS indicated that the deprotection was complete, the volatiles were removed, and the residue was purified on a Teledyne ISCO CombiFlash system, eluting a reverse phase solvent gradient of 0.1% formic acid/MeCN in 0.1% formic acid/H_2_O. Fractions found to contain the product were combined and concentrated *in vacuo* to afford the desired products as formate salts.

Representative example using this method: 3-[1-(1*H*-indole-2-carbonyl)-5-methoxy-1′-methylspiro­[2*H*-indole-3,4′-piperidine]-6-yl]­oxy-*N*,*N*-dimethylbenzenesulfonamide (**32**).

Using the general Chan–Lam method, *tert*-butyl 2-(6-hydroxy-5-methoxy-1′-methylspiro­[2*H*-indole-3,4′-piperidine]-1-carbonyl)­indole-1-carboxylate **16**, pyridine, *N*,*N*-dimethyl 3-boronobenzenesulfonamide, and Cu­(OAc)_2_ and subsequent deprotection were reacted to form the desired 3-[1-(1*H*-indole-2-carbonyl)-5-methoxy-1′-methylspiro­[2*H*-indole-3,4′-piperidine]-6-yl]­oxy-*N*,*N*-dimethylbenzenesulfonamide (**32**) as a white powder.

Yield: 51 mg (54%). ^1^H NMR (400 MHz, DMSO-*d*_6_) δ 11.67 (d, *J* = 2.2 Hz, 1H), 7.96 (s, 1H), 7.72 (d, *J* = 8.0 Hz, 1H), 7.61 (t, *J* = 8.0 Hz, 1H), 7.47 (d, *J* = 8.3 Hz, 1H), 7.42 (dt, *J* = 7.8, 1.2 Hz, 1H), 7.27 (s, 1H), 7.26–7.19 (m, 3H), 7.13 (t, *J* = 2.1 Hz, 1H), 7.08 (t, *J* = 7.3 Hz, 1H), 4.39 (s, 2H), 3.76 (s, 3H), 2.79 (d, *J* = 8.3 Hz, 2H), 2.60 (s, 6H), 2.25 (s, 3H), 2.15–1.97 (m, 4H), 1.70 (d, *J* = 10.6 Hz, 2H). LC/MS (ESI^+^) found *m*/*z* = 575.5 [M + H]^+^ (calc for C_31_H_34_N_4_O_5_S *m*/*z* = 575.2 [M + H]^+^. Purity by LC (280 nm): 98%.

## Supplementary Material





## Notes

Additional experimental procedures and assay protocols (PDF)
List of molecular strings with experimental results presented in this manuscript (CSV)

## ABBREVIATIONS

ABS: asexual blood stage
AcOH: acetic acid
ADME: absorption, distribution, metabolism, and excretion
BH_3_·SMe_2_: borane dimethylsulfide complex
BnBr: benzyl bromide
Boc: *tert*-butyloxycarbonyl
Boc_2_O: di-*tert*-butyl dicarbonate
CBZ: benzyloxy carbonyl
CHO: Chinese hamster ovary
Cl_int,app_: intrinsic clearance
COMU: (1-cyano-2-ethoxy-2-oxoethylidenminooxy)­dimethylamino-morpholino-carbenium hexafluorophosphate
Cs_2_CO_3_: cesium carbonate
Cu­(OAc)_2_: copper­(II) acetate
DCE: dichloroethane
DCM: dichloromethane
DGFA: dual gamete formation assay
DIPEA: diisopropylethylamine
DMAP: dimethylaminopyridine
DMC: 2-chloro-1,3-dimethylimidazolinium chloride
DMF: *N*,*N*-dimethylformamide
DMPK: drug metabolism and pharmacokinetics
DMSO: dimethyl sulfoxide
EDC: 1-ethyl-3-(3-(dimethylamino)­propyl)­carbodiimide
Et_2_O: diethyl ether
EtOAc: ethyl acetate
EtOH: ethanol
HATU: hexafluorophosphate azabenzotriazole tetramethyl uronium
HCl: hydrochloric acid
hERG: human ether-a-go-go-related gene
HNO_3_: nitric acid
HPLC: high-performance liquid chromatography
HTS: high-throughput screening
K_2_CO_3_: potassium carbonate
LiAlH_4_: lithium aluminum hydride
LC/MS: liquid chromatography–mass spectrometry
LDA: lithium diisopropylamine
MeCN: acetonitrile
MeOH: methanol
MgSO_4_: magnesium sulfate
MMV: Medicines for Malaria Venture
Na_2_SO_4_: sodium sulfate
NaBH_4_: sodium borohydride
NaOH: sodium hydroxide
Na*^t^* OBu: sodium *tert*-butoxide
*n*-BuOH: *n*-butanol
NEt_3_: triethylamine
NH_3_: ammonium
NH_4_Cl: ammonium chloride
NMR: nuclear magnetic spectroscopy
NSG: NOD-scid IL-2Rgamma^null^
P*b*: Plasmodium berghei
Pd_2_(dba)_3_: tris­(dibenzylideneacetone)­dipalladium­(0)
*Pf*: Plasmodium falciparum
pLDH: parasite lactate dehydrogenase
PRR: parasite reduction ratio
RuPhos: 2-dicyclohexylphosphino-2′,6′-diisopropoxy-1,1′-biphenyl
SAR: structure–activity relationship
STAB: sodium triacetoxyborohydride
SWOT: strengths, weaknesses, opportunities, and threats
TFA: trifluoroacetic acid
THF: tetrahydrofuran
TMSI: trimethylsilyl iodide
tPSA: topological polar surface area
WHO: World Health Organization
