# Supplementary material for: Cell-Based Progression of Spiroindoline Phenotypic Hits Leads to the Identification of Compounds with Diverging Parasitological Profiles against the Human Malaria Parasite Plasmodium falciparum
Source: J Med Chem. 2025 May 12;68(10):10156–72. doi: 10.1021/acs.jmedchem.5c00302 (PMC12105011; doi:10.1021/acs.jmedchem.5c00302)
Supplement: Supplementary file 1 [file jm5c00302_si_001.pdf]

SUPPORTING INFORMATION

Cell-Based Progression of Spiroindoline Phenotypic Hits Leads to the Identification of Compounds with Diverging Parasitological Profiles Against the Human Malaria Parasite *Plasmodium falciparum*

Jean Dam[†], Grant A. Boyle[†], André Horatscheck[†], John G. Woodland[†Δ], Claire Le Manach[†], Gurminder Kaur[†], Dale Taylor[†], Liezl Gibhard[†], Mathew Njoroge[†], Nina Lawrence[†], Christel Brunschwig[†], Victor Zdorichenko[‴], Brian Cox[‴], Sergio Wittlin[§Ψ], Thomas W. von Geldern[+], Dennis Smith[+], James Duffy[+], Gregory S. Basarab[†], Kelly Chibale[†#Δ]*

[†]Holistic Drug Discovery and Development (H3D) Centre, University of Cape Town, Rondebosch 7701, South Africa; [#]South African Medical Research Council, Drug Discovery and Development Research Unit, Department of Chemistry; [Δ]Institute of Infectious Disease and Molecular Medicine, University of Cape Town, Rondebosch 7701, South Africa; [‴]*Department of Chemistry, Arundel Building 305. School of Life Sciences, University of* Sussex, Falmer, Brighton Sussex, BN1 9RH, United Kingdom; [§]Swiss Tropical and Public Health Institute, Kreuzstrasse 2, 4106 Allschwil, Switzerland; [Ψ]University of Basel, 4002 Basel, Switzerland; [+]Medicines for Malaria Venture, ICC, Route de Pré-Bois 20, PO Box 1826, 1215 Geneva, Switzerland.

Corresponding author: Kelly Chibale (kelly.chibale@uct.ac.za)

Contents

A. Synthetic procedures S3

B. Screening Cascade S18

C. In vitro asexual blood stage NF54 and K1 P. falciparum screening S18

D. Cross-resistance screening S20

E. Stage Specificity S21

F. Estimation of antimalarial killing profile using two-color flow cytometry analysis (GSK, Tres Cantos) S22

G. Determination of antimalarial killing profile using a parasite reduction ratio (PRR) assay (GSK, Tres Cantos) S23

H. Cytotoxicity screening S25

I. hERG patch clamp assay and data S26

J. Metabolic stability - Microsome CLint,app S26

K. CYP inhibition assay S27

L. Solubility assay S28

M. Metabolite Identification S28

N. PK studies – Methods S30

O. In vivo antimalarial efficacy studies with *P. falciparum* conducted at H3D S31

References S33

A. Synthetic procedures

*1-(1H-Indol-2-ylmethyl)-5-methoxy-1'-methyl-6-phenylmethoxyspiro[2H-indole-3,4'-piperidine] (**24**)*

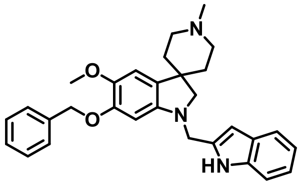

1*H*-Indol-2-yl-(5-methoxy-1'-methyl-6-phenylmethoxyspiro[2*H*-indole-3,4'-piperidine]-1-yl)methanone (**1**) (50 mg, 0.10 mmol) was dissolved in anhydrous THF (10 mL) under a N_2 atmosphere and cooled to 0 °C. LAH (12 mg, 0.31 mmol, 3 eq) was added to the solution and stirred cold for 30 min. The reaction was then warmed to 23 °C over 2 h. LCMS analysis of the crude reaction mixture showed very little conversion had occurred and so a further portion of LAH (12 mg, 0.31 mmol, 3 eq) was added. After a further 2 h of stirring at 23 °C, starting material was still evident by LCMS and so the reaction was left to stir overnight (16 h). The reaction was quenched by the addition of a few drops of water, filtered and the filtrate further dissolved in EtOAc washed with H_2O. The organic layer was dried over Na_2SO_4, filtered and concentrated *in vacuo*. The resulting residue was purified on a Teledyne ISCO CombiFlash system, eluting a gradient of 0-20% 0.5 M NH_3 in MeOH in DCM on a 4 g Silica column. After concentration *in vacuo*, the resulting solid was further dried on a freeze dryer overnight to afford 1-(1*H*-indol-2-ylmethyl)-5-methoxy-1'-methyl-6-phenylmethoxyspiro[2*H*-indole-3,4'-piperidine] **24** as a white solid.

Yield: 18 mg (37%). ^{1}H NMR (300 MHz, C*D*Cl_3) δ 8.08 (s, 1H), 7.50 (d, *J* = 7.7 Hz, 1H), 7.24 (m, 5H), 7.06 (m, 2H), 6.69 (s, 1H), 6.30 (t, *J* = 1.4 Hz, 1H), 6.19 (s, 1H), 4.97 (s, 2H), 4.19 (s, 2H), 3.75 (s, 3H), 3.05 (s, 2H), 2.85 (d, *J* = 10.6 Hz, 2H), 2.31 (s, 3H), 2.18–1.89 (m, 4H), 1.64 (d, *J* = 12.6 Hz, 2H). LC/MS (ESI$^+$) found *m/z* = 468.2 $[M+H]^+$ (calc for $C_{30}H_{34}N_3O_2$ *m/z* = 468.3 $[M+H]^+$). Purity by LC (280 nm): 99%.

*(5-Methoxy-1'-methyl-6-phenylmethoxyspiro[2H-indole-3,4'-piperidine]-1-yl)-(1H-pyrrol-2-yl)methanone (**25**)*

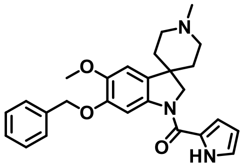

Following General Amide coupling Method B: Yield: 26 mg (57%). ^{1}H NMR (600 MHz, C*D*Cl_3) δ 9.75 (s, 1H), 8.08 (s, 1H), 7.48 (d, *J* = 7.6 Hz, 2H), 7.39–7.34 (m, 2H), 7.32–7.27 (m, 1H), 7.02 (m, 1H), 6.78 (s, 1H), 6.72 (m, 1H), 6.37–6.33 (m, 1H), 5.16 (s, 2H), 4.19 (s, 2H), 3.85 (d, *J* = 0.9 Hz, 3H), 2.95 (d, *J* = 10.4 Hz, 2H), 2.40 (s, 3H), 2.17–2.04 (m, 4H), 1.71 (d, *J* = 12.2 Hz, 2H). LC/MS (ESI$^+$) found *m/z* = 432.2 $[M+H]^+$ (calc for $C_{26}H_{29}N_3O_3$ *m/z* = 432.2 $[M+H]^+$). Purity by LC (254 nm): 96%.

*(5,6-Dimethoxy-1'-methylspiro[2H-indole-3,4'-piperidine]-1-yl)-(1H-indol-2-yl)methanone (**26**)*

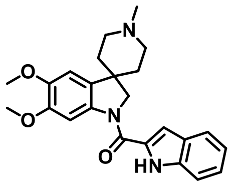

Step 1: To ice cold HNO_3 (1.03 ml, 12.8 mmol, 1 eq) was added dropwise with stirring 1,2-dimethoxy-4-fluorobenzene (2.00 g, 12.8 mmol) dissolved in AcOH (26 ml). The mixture was stirred at 0 °C for 15 minutes, then brought to 23 °C for 15 mins. The orange solution was poured on ice and the resultant pale-yellow solid was filtered and washed with H_2O (3x 20 ml). The solid was taken up in EtOAc and washed with brine. The organic phase was dried over $MgSO_4$, evaporated and dried under high vacuum to give *1-fluoro-4,5-dimethoxy-2-nitrobenzene* as a peach coloured solid.

Yield: 1.50 g (58%). ^{1}H NMR (300 MHz, C*D*Cl$_3$) δ 7.58 (d, *J* = 7.1 Hz, 1H), 6.73 (d, *J* = 12.3 Hz, 1H), 3.96 (s, 3H), 3.93 (s, 3H). LC/MS (ESI$^+$) found *m/z* = 202.1 $[M+H]^+$ (calc for $C_8H_8FNO_4$ *m/z* = 202.1 $[M+H]^+$).

Step 2: A clean, oven dried 2-neck RB flask was fitted with a dropping funnel and a N_2 line. The setup was placed under vacuum and filled with N_2 (x3). LDA solution in THF/Hexanes (1.0 M, 1.11 ml, 7.46 mmol, 1 eq) was added to the flask and diluted with anhydrous THF (15 ml) under N_2 before being cooled to -78 °C. Ethyl *N*-Boc-piperidine-4-carboxylate (1.92 g, 7.46 mmol, 1 eq) dissolved in anhydrous THF (10 ml) was added to the dropping funnel and added to the LDA solution dropwise. The solution was left to stir at -78 °C for 1 h. A solution of anhydrous THF (12 ml) and 1-fluoro-4,5-dimethoxy-2-nitrobenzene (1.50 g, 7.46 mmol) was added to the dropping funnel and introduced to the reaction dropwise. The reaction was slowly warmed to 23 °C over 1 h and the reaction stirred at 23 °C for 1 h. Saturated aqueous NH_4Cl was added to quench the reaction and the reaction mixture transferred to a separation funnel. After dilution with EtOAc, the solution was washed with H_2O followed by brine and the organic layer dried over Na_2SO_4, filtered and concentrated *in vacuo*. The resulting brown gum was purified on a Teledyne ISCO CombiFlash system, eluting a gradient of 0-50% EtOAc in hexane. The product, *1-O-tert-butyl 4-O-ethyl 4-(4,5-dimethoxy-2-nitrophenyl)piperidine-1,4-dicarboxylate* was collected as a bright yellow gum.

Yield: 2.28 g (61%). ^{1}H NMR (300 MHz, C*D*Cl$_3$) δ 7.48 (s, 1H), 6.99 (s, 1H), 4.16 (q, *J* = 7.1 Hz, 2H), 3.99 (s, 3H), 3.93 (s, 3H), 3.58 (s, 4H), 2.46–2.23 (m, 2H), 2.08–1.75 (m, 2H), 1.45 (s, 9H), 1.21 (t, *J* = 7.1 Hz, 3H). LC/MS (ESI$^+$) found *m/z* = 339.2 $[M+H-100]^+$ (calc for $C_{16}H_{22}N_2O_6$ *m/z* = 339.2 $[M+H-100]^+$).

Step 3: Zinc (2.99 g, 45.7 mmol, 10 eq) was weighed into a 250 ml RB flask. 2% aqueous HCl solution (35 ml) was added to the flask and stirred for 5 min at 23 °C. The solution was decanted and a further 35 ml portion of 2% aqueous HCl solution added. After stirring for 5 min the solution was decanted. H_2O (50 ml) was added to the flask and decanted (this process was repeated twice). AcOH (20 ml) was added to the zinc and the solution warmed to 50 °C. 1-*O*-*tert*-Butyl 4-*O*-ethyl 4-(4,5-dimethoxy-2-nitrophenyl)piperidine-1,4-dicarboxylate (2.28 g, 4.57 mmol) was dissolved in AcOH (10 ml) and added to a dropping funnel above the stirring zinc solution. This was further diluted with the remaining AcOH and introduced dropwise to the zinc solution below. The reaction was warmed to 60 °C and allowed to stir for 2 h. On cooling, the unreacted Zn and $Zn(OAc)_2$ by-products were filtered off through a celite plug and washed with DCM. The filtrate was transferred to a separation funnel, diluted with more DCM and washed with H_2O (50 ml x3). The organic phase was dried over $MgSO_4$, filtered and concentrated *in vacuo*. A small

amount of EtOAc was added to the resulting red gum and a light pink precipitate resulted upon sonication. The precipitate was collected by filtration and determined to be the desired product, *tert-butyl 5,6-dimethoxy-2-oxospiro[1H-indole-3,4'-piperidine]-1'-carboxylate* which was used without further purification in the next reaction.

Yield: 740 mg (27%). ^{1}H NMR (300 MHz, C*D*Cl$_3$) δ 8.23 (s, 1H), 6.84 (s, 1H), 6.55 (s, 1H), 3.88 (s, 3H), 3.86 (s, 3H), 3.82 (dd, *J* = 8.4, 4.4 Hz, 4H), 1.92–1.69 (m, 4H), 1.50 (s, 9H). LC/MS (ESI$^-$) found *m/z* = 361.1 [M-H]$^-$ (calc for $C_{19}H_{26}N_2O_5$ *m/z* = 361.2 [M-H]$^-$).

Step 4: In a dry microwave tube, *tert*-butyl 5,6-dimethoxy-2-oxospiro[1*H*-indole-3,4'-piperidine]-1'-carboxylate (200 mg, 0.550 mmol) was dissolved in anhydrous THF (5.5 ml) under an N_2 atmosphere. LAH (105 mg, 2.76 mmol, 5 eq) was added, the reaction tube sealed and heated at 80 °C for 2 h. On cooling, H_2O was slowly added to quench the reaction. The reaction mixture was filtered and the filter cake washed with EtOAc. After transferring the solution to a separation funnel, the reaction mixture was diluted with EtOAc and washed with a further portion of H_2O. The organic layer was dried over $MgSO_4$, filtered and concentrated *in vacuo*. The resulting light brown solid was used in the amide coupling reaction without further purification.

Yield: 94 mg (64%). ^{1}H NMR (300 MHz, C*D*Cl$_3$) δ 6.70 (s, 1H), 6.33 (s, 1H), 3.81 (s, 3H), 3.79 (s, 3H), 3.41 (s, 2H), 2.82 (dt, *J* = 11.8, 3.2 Hz, 2H), 2.32 (s, 3H), 2.12–1.99 (m, 2H), 1.93 (td, *J* = 12.7, 3.7 Hz, 2H), 1.77–1.65 (m, 2H). LC/MS (ESI$^+$) found *m/z* = 263.2 [M+H]$^+$ (calc for $C_{15}H_{22}N_2O_2$ *m/z* = 263.2 [M+H]$^+$).

Step 5: 5,6-Dimethoxy-1'-methylspiro[indoline-3,4'-piperidine] was transformed into **26** using General Amide Coupling Procedure A. Following purification, *(5,6-Dimethoxy-1'-methylspiro[2H-indole-3,4'-piperidine]-1-yl)-(1H-indol-2-yl)methanone (**26**)* was collected as a pale-yellow solid.

Yield: 83 mg (57%). ^{1}H NMR (300 MHz, C*D*Cl$_3$) δ 9.56 (s, 1H), 8.06 (s, 1H), 7.75 (dd, *J* = 8.0, 1.1 Hz, 1H), 7.47 (dd, *J* = 8.3, 1.0 Hz, 1H), 7.33 (m, 1H), 7.18 (m, 1H), 7.02 (dd, *J* = 2.1, 0.9 Hz, 1H), 6.78 (s, 1H), 4.34 (s, 2H), 3.93 (s, 3H), 3.87 (s, 3H), 2.94 (d, *J* = 8.6 Hz, 2H), 2.40 (s, 3H), 2.22–1.99 (m, 4H), 1.72 (s, 2H). LC/MS (ESI$^+$) found *m/z* = 406.2 [M+H]$^+$ (calc for $C_{24}H_{27}N_3O_3$ *m/z* = 406.2 [M+H]$^+$). Purity by LC (280 nm): 100%.

*1H-Indol-2-yl-(5-methoxy-1'-methyl-6-prop-2-ynoxyspiro[2H-indole-3,4'-piperidine]-1-yl)methanone (**28**)*

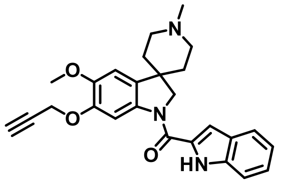

Following the General procedure for one pot SN2 displacement and Boc deprotection at R^6: Yield: 11 mg (12%). ^{1}H NMR (400 MHz, DMSO-*d*$_6$) δ 11.73 (s, 1H), 8.06 (s, 1H), 7.72 (d, *J* = 8.0 Hz, 1H), 7.47 (d, *J* = 8.2 Hz, 1H), 7.28–7.17 (m, 2H), 7.08 (t, *J* = 7.5 Hz, 1H), 7.01 (s, 1H), 4.74 (d, *J* = 2.4 Hz, 2H), 4.34 (s, 2H), 3.79 (s, 3H), 3.57–3.51 (m, 1H), 2.82 (d, *J* = 11.6 Hz, 2H), 2.28 (s, 3H), 2.17 (d, *J* = 15.6 Hz, 2H), 1.98 (td, *J* = 13.2, 4.0 Hz, 2H), 1.64 (d, *J* = 12.8 Hz, 2H). LC/MS (ESI$^+$) found *m/z* = 430.2 [M+H]$^+$ (calc for $C_{26}H_{27}N_3O_3$ m/z = 430.2 [M+H]$^+$). Purity by LC (280 nm): 97%.

*1H-Indol-2-yl-[5-methoxy-1'-methyl-6-(2,2,2-trifluoroethoxy)spiro[2H-indole-3,4'-piperidine]-1-yl]methanone (**29**)*

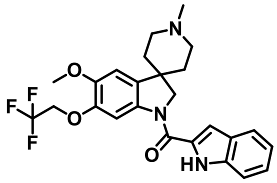

Following the General procedure for one pot SN2 displacement and Boc deprotection at R^6: Yield: 64 mg (67%). ^{1}H NMR (300 MHz, Methanol-d_4) δ 7.92 (s, 1H), 7.70 (d, *J* = 8.0 Hz, 1H), 7.49 (d, *J* = 8.3 Hz, 1H), 7.27 (t, *J* = 7.6 Hz, 1H), 7.15 (s, 1H), 7.10 (t, *J* = 7.5 Hz, 1H), 7.00 (s, 1H), 4.47 (q, *J* = 8.7 Hz, 2H), 4.39 (s, 2H), 3.89 (s, 3H), 2.92 (d, *J* = 12.0 Hz, 2H), 2.37 (s, 3H), 2.30–2.17 (m, 2H), 2.02 (td, *J* = 13.2, 4.0 Hz, 2H), 1.73 (d, *J* = 13.2 Hz, 2H). LC/MS (ESI$^+$) found *m/z* = 474.4 [M+H]$^+$ (calc for $C_{25}H_{26}F_3N_3O_3$ *m/z* = 474.2 [M+H]$^+$). Purity by LC (280 nm): 100%.

*(1H-Indol-2-yl)(5-methoxy-6-phenethylspiro[indoline-3,4'-piperidin]-1-yl)methanone (**30**)*

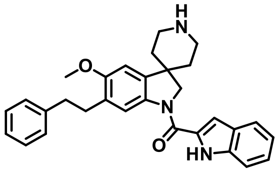

Step 1 - Into a solution of *tert*-butyl 6-(benzyloxy)-5-methoxy-2-oxospiro[indoline-3,4'-piperidine]-1'-carboxylate (**7**) (2.00 g, 4.56 mmol, 1 eq) in THF (25 ml) under an N_2 atmosphere was added 10% palladium on carbon (0.243 g, 106 mmol, 0.5 eq). The suspension was deoxygenated under vacuum, purged with hydrogen several times and then stirred under hydrogen (15 psi) at 25 °C for 1 h. The mixture was filtered through celite and then concentrated under reduced pressure to give *tert-butyl 6-hydroxy-5-methoxy-2-oxospiro[indoline-3,4'-piperidine]-1'-carboxylate (**5**)*, as a red/brown solid, which was used in the next step without further purification.

Yield: 1.12 g (70%); ^{1}H NMR (400 MHz, DMSO-d_6): δ 10.04 (s, 1H), 8.97 (s, 1H), 7.03 (s, 1H), 6.36 (s, 1H), 3.71 (s, 3H), 3.67-3.53 (m, 4H), 1.61 (s, 4H), 1.43 (s, 9H).

Step 2 - 1,1,1-Trifluoro-*N*-phenyl-*N*-((trifluoromethyl)sulfonyl)methanesulfonamide (1.26 g, 3.54 mmol, 1.1 eq) was added portion wise under an N_2 atmosphere into a solution of *tert*-butyl 6-hydroxy-5-methoxy-2-oxospiro[indoline-3,4'-piperidine]-1'-carboxylate (1.12 g, 3.22 mmol, 1.0 eq) and triethylamine (1.79 ml, 12.9 mmol, 4.0 eq) in DCM (25 ml) at 0 °C. The solution was stirred at 23 °C for 12 h, diluted with a saturated solution of sat. $NaHCO_3$ (20 mL) and then extracted with DCM (3 × 20 ml). The combined organic layers were washed with brine (20 ml), dried over anhydrous Na_2SO_4, filtered and then concentrated under reduced pressure to give *tert-butyl 5-methoxy-2-oxo-6-(((trifluoromethyl)sulfonyl)oxy)spiro[indoline-3,4'-piperidine]-1'-carboxylate* as a yellow solid, which was used in the next step without further purification.

Yield: 1.51 g (97%); ^{1}H NMR (400 MHz, DMSO-d_6): δ 10.38 (s, 1H), 7.51 (s, 1H), 6.84 (s, 1H), 3.87 (s, 3H), 3.76-3.73 (m, 2H), 3.62 (br s, 2H), 1.79 (d, *J* = 8.9 Hz, 2H), 1.67 (d, *J* = 13.4 Hz, 2H), 1.43 (s, 9H).

Step 3 - Into a solution of *tert*-butyl 5-methoxy-2-oxo-6-(((trifluoromethyl)sulfonyl)oxy)spiro[indoline-3,4'-piperidine]-1'-carboxylate (1.6 g, 3.33 mmol, 1 eq), (*E*)-styrylboronic acid (1.48 g, 9.99 mmol, 3.0 eq) and K_2CO_3 (1.38 g, 9.99 mmol, 3 eq) in DMF/water (18 ml/0.05 ml) under an atmosphere of N_2 was added [1,1'-bis(diphenylphosphino)ferrocene]dichloropalladium (0.24 g, 0.33 mmol, 0.1 eq). The reaction was heated at 90 °C for 11 h, cooled and then filtered. The filtrate was diluted with EtOAc (30 ml), washed with brine (x3), dried over anhydrous Na_2SO_4, filtered and then concentrated under reduced pressure to obtain crude solid. *tert-Butyl (E)-5-methoxy-2-oxo-6-styrylspiro[indoline-3,4'-piperidine]-1'-carboxylate* was isolated as a white solid after column chromatography on silica, eluting with mixtures of 20% EtOAc in hexane.

Yield: 0.62 g (43%); ^{1}H NMR (400 MHz, DMSO-*d*$_6$): δ 10.31 (s, 1H), 7.56 (d, *J* = 8.9 Hz, 2H), 7.44-7.30 (m, 3H), 7.27-7.24 (m, 1H), 7.18 (s, 1H), 7.13 (s, 1H), 7.08 (s, 1H), 3.85 (s, 3H), 3.71 (s, 4H), 1.98-1.66 (m, 4H), 1.44 (s, 9H).

Step 4 - Into a solution of *tert*-butyl (*E*)-5-methoxy-2-oxo-6-styrylspiro[indoline-3,4'-piperidine]-1'-carboxylate in THF (25 ml) and MeOH (10 ml) under an N_2 atmosphere was added 10% palladium on carbon (0.2 g). The suspension was deoxygenated under vacuum, purged with hydrogen several times and then stirred under hydrogen (15 psi) at 25 °C for 12 h. The mixture was filtered and then concentrated under reduced pressure to give *tert-butyl 5-methoxy-2-oxo-6-phenethylspiro[indoline-3,4'-piperidine]-1'-carboxylate*, as a red/brown solid, which was used in the next step without further purification.

Yield: 0.67 g (98%); ^{1}H NMR (400 MHz, DMSO-*d*$_6$): δ 10.17 (s, 1H), 7.29-7.16 (m, 5H), 7.09 (s, 1H), 6.65 (s, 1H), 3.78 (s, 3H), 3.68 (s, 4H), 2.78 (s, 4H), 1.75-1.62 (m, 4H), 1.43 (s, 9H).

Step 5 - Into a solution of *tert*-butyl 5-methoxy-2-oxo-6-phenethylspiro[indoline-3,4'-piperidine]-1'-carboxylate (0.70 g, 1.60 mmol, 1 eq) in THF (25 ml) at 25 °C under an N_2 atmosphere was added a ~10 M solution of borane dimethyl sulfide complex (0.80 ml, 8.02 mmol, 5 eq) dropwise. The reaction was heated at 70 °C for 18 h, cooled to 25 °C and then quenched with methanol (5 ml). The mixture was concentrated under reduced pressure, diluted with water (20 ml) and then extracted with EtOAc (×2). The combined organic layers were washed with brine, dried over anhydrous Na_2SO_4, filtered and then concentrated under reduced pressure. The resulting residue was purified by recrystallization from EtOAc/pentane and kept in a fridge for 2 days to obtain *tert-butyl 5-methoxy-6-phenethylspiro[indoline-3,4'-piperidine]-1'-carboxylate* as a white solid.

Yield: 0.4 g (59%); ^{1}H NMR (400 MHz, DMSO-*d*$_6$): δ 7.29-7.16 (m, 5H), 6.74 (s, 1H), 6.38 (s, 1H), 5.04 (s, 1H), 3.92-3.89 (m, 2H), 3.70 (s, 3H), 3.29 (s, 2H), 2.74-2.71 (m, 2H), 2.70-2.67 (m, 4H), 1.67-1.63 (m, 2H), 1.56-1.52 (m, 2H), 1.42 (s, 9H).

Step 6 - To a solution of *tert*-butyl 5-methoxy-6-phenethylspiro[indoline-3,4'-piperidine]-1'-carboxylate (0.50 g, 1.18 mmol, 1.0 eq) in DMF (5 ml) was added DIPEA (0.51 ml, 2.96 mmol, 2.5 eq) followed by HATU (0.67 g, 1.78 mmol, 1.5 eq). To the resulting solution 1*H*-indole-2-carboxylic acid (0.55 g, 1.3 mmol, 1.1 eq) was added and the resulting reaction was allowed to stir at 25 °C for 15 h under a N_2 atmosphere, then saturated NH_4Cl solution was added to stop the reaction, extracted with EtOAc and washed with saturated brine solution. The organic portion was dried over Na_2SO_4 and concentrated to provide a crude residue which was purified by preparative HPLC normal phase chromatography (SFC). Column name: - Chiralpak IG (250 x 20mm, 5µ) operating at ambient temperature and flow rate of 21.0 ml/min. Mobile phase: n-Hexane/EtOH/DCM - 60/20/20 at 320 nm wavelength with a run time 17 min.

The combined pure fractions were distilled out and followed by lyophilization to give *tert-butyl 1-(1H-indole-2-carbonyl)-5-methoxy-6-phenethylspiro[indoline-3,4'-piperidine]-1'-carboxylate* as a white solid.

Yield: 0.140 g (70%); ^{1}H NMR (400 MHz, C*D*Cl$_3$): δ 9.42 (s, 1H), 8.20 (s, 1H), 7.21 (d, *J* = 8.0 Hz, 1H), 7.46 (d, *J* = 8.0 Hz, 1H), 7.34-7.24 (m, 5H), 7.18-7.15 (m, 2H), 6.98 (s, 1H), 6.68 (s,1H), 4.39 (s, 2H), 4.20-4.19 (m, 2H), 3.83 (s, 3H), 3.04-2.90 (m, 6H), 1.91-1.78 (m, 2H), 1.74- 1.71(m, 2H), 1.49 (s, 9H). LC/MS (ESI$^+$) found *m/z* = 566 [M+H]$^+$ (calc for $C_{35}H_{39}N_3O_4$ *m/z* = 566 [M+H]$^+$). HPLC Purity: 95%.

Step 7 - Into a solution of *tert*-butyl 1-(1*H*-indole-2-carbonyl)-5-methoxy-6-phenethylspiro[indoline-3,4'-piperidine]-1'-carboxylate (0.130 g, 0.23 mmol, 1.0 eq) in EtOAc (0.5 ml) at 25 °C was added a 4 N solution of HCl in EtOAc (0.025 ml, 0.712 mmol, 3.1 eq). The reaction was stirred for 5 h and then concentrated under reduced pressure followed by lyophilization to give the desired product *(1H-Indol-2-yl)(5-methoxy-6-phenethylspiro[indoline-3,4'-piperidin]-1-yl)methanone hydrochloride* **30** as a white solid.

Yield: 0.08 g (75%); ^{1}H NMR (400 MHz, DMSO-*d$_6$*): δ 11.65 (s, 1H), 8.75 (brs, 2H), 8.12 (s, 1H), 7.69 (d, *J* = 8.0 Hz, 1H), 7.49 (d, *J* = 8.2 Hz, 1H), 7.34-7.17 (m, 7H), 7.08 (t, *J* = 7.52 Hz, 1H), 6.82 (s, 1H), 4.47 (s, 2H), 3.85 (s, 3H), 3.31 (s, 1H), 3.19 (t, *J* = 12.7 Hz, 2H), 2.84 (s, 4H), 2.14 (t, *J* = 10.76 Hz, 2H), 1.86 (d, *J* = 13.84 Hz, 2H); ^{1}H NMR (400 MHz, DMSO-*d$_6$* containing few drops of D_2O): δ 8.64 (s, 1H), 7.68 (d, *J* = 8.04 Hz, 1H), 7.49 (d, *J* = 7.96 Hz, 1H), 7.27-6.16 (m, 7H), 7.08 (t, *J* = 8.0 Hz, 1H), 6.89 (s, 1H), 4.43 (s, 2H), 3.81 (s, 3H), 3.30 (d, *J* = 12.44 Hz, 2H), 3.13 (t, *J* = 12.2 Hz, 2H), 2.81 (s, 4H), 2.04 (t, *J* = 11.92 Hz, 2H), 1.86 (t, *J* = 1.44 Hz, 2H); LC/MS (ESI$^+$) found *m/z* = 466 [M+H]$^+$ (calc for $C_{30}H_{32}N_3O_2$ *m/z* = 466 [M+H]$^+$). Purity by LCMS (280 nm): 96%.

*1H-indol-2-yl-(5-methoxy-1'-methyl-6-phenoxyspiro[2H-indole-3,4'-piperidine]-1-yl)methanone (**31**)*

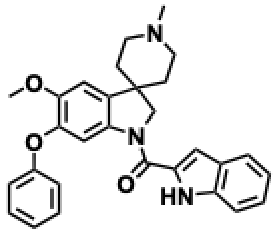

Following the general procedure of Chan-Lam reactions: Yield: 12 mg (15%). ^{1}H NMR (300 MHz, *Methanol-d$_4$*) δ 7.87 (s, 1H), 7.68 (d, *J* = 8.1 Hz, 1H), 7.45 (d, *J* = 8.3 Hz, 1H), 7.32–7.16 (m, 4H), 7.14–6.95 (m, 3H), 6.87 (d, *J* = 8.1 Hz, 2H), 4.50 (s, 2H), 3.82 (s, 3H), 3.36 (m, 2H), 2.91 (t, *J* = 12.9 Hz, 2H), 2.75 (s, 3H), 2.21 (t, *J* = 13.9 Hz, 2H), 1.97 (d, *J* = 14.1 Hz, 2H). LC/MS (ESI$^+$) found *m/z* = 468.2 [M+H]$^+$ (calc for $C_{31}H_{33}N_3O_4$ *m/z* = 468.2 [M+H]$^+$). Purity by LC (280 nm): 100%.

*1H-Indol-2-yl-[5-methoxy-6-[(4-methoxyphenyl)methoxy]-1'-methylspiro[2H-indole-3,4'-piperidine]-1-yl]methanone (**34**)*

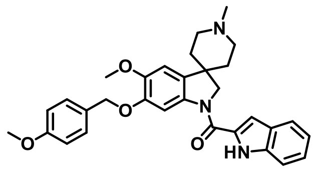

Following the General procedure for one pot SN2 displacement and Boc deprotection at R^6: Yield: 12 mg (12%). ^{1}H NMR (400 MHz, Methanol-d_4) δ 8.56 (s, 1H - formic acid), 7.97 (s, 1H), 7.70 (d, *J* = 8.1 Hz, 1H), 7.49 (d, *J* = 8.3 Hz, 1H), 7.34 (s, 2H), 7.27 (t, *J* = 7.6 Hz, 1H), 7.16 (s, 1H), 7.10 (t, *J* = 7.5 Hz, 1H), 6.98–6.85 (m, 3H), 4.99 (s, 2H), 4.39 (s, 2H), 3.87 (d, *J* = 7.6 Hz, 3H), 3.78 (s, 3H), 3.10 (d, *J* = 12.2 Hz, 2H), 2.55 (d, *J* = 6.9 Hz, 5H), 2.07 (t, *J* = 13.2 Hz, 2H), 1.80 (d, *J* = 13.9 Hz, 2H). LC/MS (ESI$^+$) found *m/z* = 512.3 [M+H]$^+$ (calc for $C_{31}H_{33}N_3O_4$ *m/z* = 512.3 [M+H]$^+$). Purity by LC (280 nm): 100%.

*[6-[(4-Fluorophenyl)methoxy]-5-methoxy-1'-methylspiro[2H-indole-3,4'-piperidine]-1-yl]-(1H-indol-2-yl)methanone (**35**)*

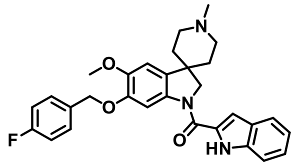

Following the General procedure for one pot SN2 displacement and Boc deprotection at R^6: Yield: 49 mg (47%). ^{1}H NMR (300 MHz, DMSO-d_6) δ 11.66 (d, *J* = 2.1 Hz, 1H), 7.96 (s, 1H), 7.71 (d, *J* = 8.0 Hz, 1H), 7.49 (dd, *J* = 8.4, 5.4 Hz, 3H), 7.28–7.14 (m, 4H), 7.08 (td, *J* = 7.4, 6.9, 1.1 Hz, 1H), 7.01 (s, 1H), 5.05 (s, 2H), 4.31 (s, 2H), 3.80 (s, 3H), 2.76 (d, *J* = 10.9 Hz, 2H), 2.23 (s, 3H), 2.12–1.89 (m, 4H), 1.60 (d, *J* = 12.0 Hz, 2H). LC/MS (ESI$^+$) found *m/z* = 500.2 [M+H]$^+$ (calc for $C_{30}H_{30}FN_3O_3$ *m/z* = 500.2 [M+H]$^+$). Purity by LC (280 nm): 97%.

*[6-[(3-Fluorophenyl)methoxy]-5-methoxy-1'-methylspiro[2H-indole-3,4'-piperidine]-1-yl]-(1H-indol-2-yl)methanone (**36**)*

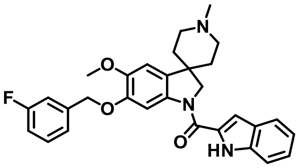

Following the General procedure for one pot SN2 displacement and Boc deprotection at R^6: Yield: 19 mg (19%). ^{1}H NMR (400 MHz, Methanol-d_4) δ 7.93 (s, 1H), 7.70 (d, *J* = 8.0 Hz, 1H), 7.48 (d, *J* = 8.3 Hz, 1H), 7.39–7.31 (m, 1H), 7.31–7.24 (m, 1H), 7.24–7.15 (m, 2H), 7.15–7.06 (m, 2H), 7.01 (td, *J* = 8.6, 2.7 Hz, 1H), 6.96 (s, 1H), 5.07 (s, 2H), 4.36 (s, 2H), 3.88 (s, 3H), 2.93 (d, *J* = 12.0 Hz, 2H), 2.38 (s, 3H), 2.25 (t, *J* = 12.3 Hz, 2H), 2.01 (td, *J* = 13.4, 4.1 Hz, 2H), 1.72 (d, *J* = 13.5 Hz, 2H). LC/MS (ESI$^+$) found *m/z* = 500.2 [M+H]$^+$ (calc for $C_{30}H_{30}FN_3O_3$ *m/z* = 500.2 [M+H]$^+$). Purity by LC (280 nm): 100%.

*[6-[(2-Fluorophenyl)methoxy]-5-methoxy-1'-methylspiro[2H-indole-3,4'-piperidine]-1-yl]-(1H-indol-2-yl)methanone (**37**)*

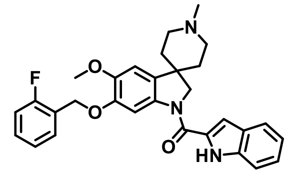

Following the General procedure for one pot SN2 displacement and Boc deprotection at R^6: Yield: 24 mg (24%). ^1H NMR (400 MHz, DMSO-d_6) δ 11.69 (d, *J* = 2.2 Hz, 1H), 8.01 (s, 1H), 7.71 (d, *J* = 8.0 Hz, 1H), 7.54 (td, *J* = 7.5, 1.8 Hz, 1H), 7.48 (d, *J* = 8.3 Hz, 1H), 7.42 (m, 1H), 7.29–7.20 (m, 3H), 7.18 (d, *J* = 2.2 Hz, 1H), 7.08 (m, 1H), 7.02 (s, 1H), 5.12 (s, 2H), 4.32 (s, 2H), 3.80 (s, 3H), 2.80 (d, *J* = 11.2 Hz, 2H), 2.26 (s, 3H), 2.10 (d, *J* = 13.6 Hz, 2H), 1.97 (td, *J* = 13.1, 4.0 Hz, 2H), 1.62 (d, *J* = 12.6 Hz, 2H). LC/MS (ESI$^+$) found *m/z* = 500.3 $[M+H]^+$ (calc for $C_{30}H_{30}FN_3O_3$ *m/z* = 500.2 $[M+H]^+$). Purity by LC (280 nm): 100%.

*1-Benzofuran-2-yl-(5-methoxy-1'-methyl-6-phenylmethoxyspiro[2H-indole-3,4'-piperidine]-1-yl)methanone (**39**)*

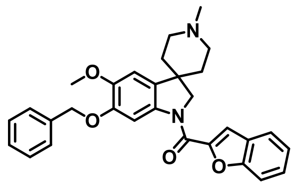

Following General Amide Coupling Method B: Yield: 39 mg (79%). ^1H NMR (600 MHz, C*D*Cl$_3$) δ 8.14 (s, 1H), 7.72 (dd, *J* = 7.7, 1.2 Hz, 1H), 7.60 (d, *J* = 8.3 Hz, 1H), 7.54–7.43 (m, 4H), 7.40–7.32 (m, 3H), 7.29 (d, *J* = 7.1 Hz, 1H), 6.80 (s, 1H), 5.18 (s, 2H), 4.36 (s, 2H), 3.87 (d, *J* = 1.0 Hz, 3H), 2.97 (d, *J* = 10.2 Hz, 2H), 2.41 (s, 3H), 2.12 (d, *J* = 16.0 Hz, 4H), 1.75 (d, *J* = 12.2 Hz, 2H). LC/MS (ESI$^+$) found *m/z* = 483.2 $[M+H]^+$ (calc for $C_{30}H_{30}N_2O_4$ *m/z* = 483.2 $[M+H]^+$). Purity by LC (254 nm): 98%.

*Imidazo[1,2-a]pyridin-2-yl-(5-methoxy-1'-methyl-6-phenylmethoxyspiro[2H-indole-3,4'-piperidine]-1-yl)methanone (**40**)*

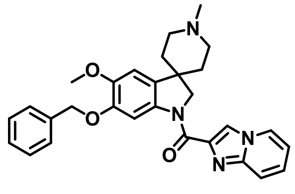

Following General Amide Coupling Method C: Yield: 24 mg (50%). ^1H NMR (600 MHz, Methanol-d_4) δ 8.51 (d, *J* = 6.8 Hz, 1H), 8.37 (s, 1H), 8.07 (s, 1H), 7.66 (d, *J* = 9.2 Hz, 1H), 7.49 (s, 2H), 7.42 (t, *J* = 8.0 Hz, 1H), 7.37 (s, 2H), 7.31 (s, 1H), 7.02 (t, *J* = 6.7 Hz, 1H), 6.97 (s, 1H), 5.15 (s, 2H), 4.47 (s, 2H), 3.89 (s, 3H), 2.91 (d, *J* = 11.9 Hz, 2H), 2.35 (s, 3H), 2.19 (t, *J* = 12.3 Hz, 2H), 2.01 (td, *J* = 13.3, 4.1 Hz, 2H), 1.72 (d, *J* = 13.5 Hz, 2H). LC/MS (ESI$^+$) found *m/z* = 483.4 $[M+H]^+$ (calc for $C_{29}H_{30}N_4O_3^+$ *m/z* = 483.2 $[M+H]^+$). Purity by LC (254 nm): 100%.

*(5-Fluoro-1H-indol-2-yl)-(5-methoxy-1'-methyl-6-phenylmethoxyspiro[2H-indole-3,4'-piperidine]-1-yl)methanone (**41**)*

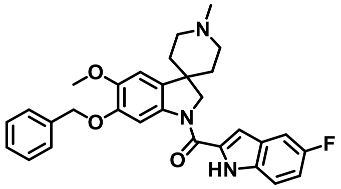

Following General Amide Coupling B: Yield: 35 mg (69 %). ^{1}H NMR (600 MHz, C*D*Cl$_3$) δ 9.66 (s, 1H), 8.13 (s, 1H), 7.46 (d, *J* = 6.5 Hz, 2H), 7.41–7.32 (m, 4H), 7.28 (t, *J* = 7.4 Hz, 1H), 7.07 (td, *J* = 9.0, 2.5 Hz, 1H), 6.94 (d, *J* = 2.1 Hz, 1H), 6.81 (s, 1H), 5.16 (s, 2H), 4.30 (s, 2H), 3.87 (s, 3H), 3.03–2.93 (m, 2H), 2.42 (s, 3H), 2.14 (t, *J* = 16.3 Hz, 5H). LC/MS (ESI$^+$) found *m/z* = 500.2 [M+H]$^+$ (calc for $C_{30}H_{30}FN_3O_3$ *m/z* = 500.2 [M+H]$^+$). Purity by LC (254 nm): 98%.

*(5-Methoxy-1H-indol-2-yl)-(5-methoxy-1'-methyl-6-phenylmethoxyspiro[2H-indole-3,4'-piperidine]-1-yl)methanone (**42**)*

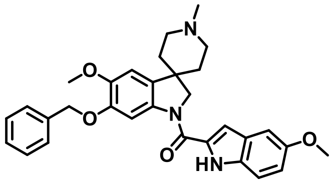

Following General Amide Coupling Method A: Yield: 43 mg (37%). ^{1}H NMR (300 MHz, DMSO-*d$_6$*) δ 11.52 (d, *J* = 2.3 Hz, 1H), 7.97 (s, 1H), 7.49–7.28 (m, 6H), 7.17 (d, *J* = 2.4 Hz, 1H), 7.07 (d, *J* = 2.2 Hz, 1H), 7.00 (s, 1H), 6.89 (dd, *J* = 8.9, 2.4 Hz, 1H), 5.07 (s, 2H), 4.29 (s, 2H), 3.81 (s, 3H), 3.78 (s, 3H), 2.76 (d, *J* = 10.3 Hz, 2H), 2.23 (s, 3H), 2.10–1.88 (m, 4H), 1.60 (d, *J* = 11.5 Hz, 2H). LC/MS (ESI$^+$) found *m/z* = 512.3 [M+H]$^+$ (calc for $C_{31}H_{33}N_3O_4$ *m/z* = 512.3 [M+H]$^+$). Purity by LC (280 nm): 100%.

*(5-Methoxy-1'-methyl-6-phenylmethoxyspiro[2H-indole-3,4'-piperidine]-1-yl)-(1H-pyrrolo[3,2-c]pyridin-2-yl)methanone (**43**)*

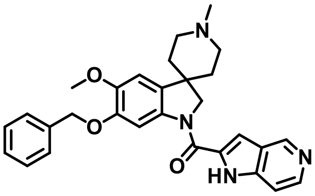

Following General Amide Coupling Method B: Yield: 11 mg (23%). ^{1}H NMR (600 MHz, C*D*Cl$_3$) δ 9.84 (s, 1H), 9.09 (s, 1H), 8.41 (d, *J* = 5.8 Hz, 1H), 8.13 (s, 1H), 7.47 (d, *J* = 7.5 Hz, 2H), 7.36 (t, *J* = 7.3 Hz, 3H), 7.29 (t, *J* = 7.3 Hz, 1H), 7.07 (s, 1H), 6.81 (s, 1H), 5.18 (s, 2H), 4.32 (s, 2H), 3.88 (s, 3H), 2.97 (d, *J* = 10.6 Hz, 2H), 2.41

(s, 3H), 2.12 (m, 4H), 1.74 (d, *J* = 12.4 Hz, 2H). LC/MS (ESI$^+$) found *m/z* = 483.1 [M+H]$^+$ (calc for $C_{29}H_{30}N_4O_3$ *m/z* = 483.2 [M+H]$^+$). Purity by LC (280 nm): 100%.

*(5-Methoxy-1'-methyl-6-phenylmethoxyspiro[2H-indole-3,4'-piperidine]-1-yl)-(1-methylpiperidin-3-yl)methanone (**44**)*

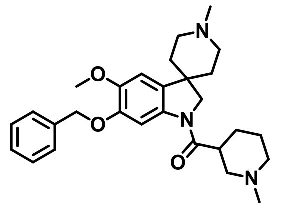

Following General Amide Coupling Method C: Yield: 4 mg (8%). ^{1}H NMR (600 MHz, C*D*Cl$_3$) δ 8.09 (s, 1H), 7.46 (d, *J* = 7.6 Hz, 2H), 7.35 (t, *J* = 7.5 Hz, 2H), 7.29 (t, *J* = 7.4 Hz, 1H), 6.73 (s, 1H), 5.12 (s, 2H), 3.98 (d, *J* = 10.3 Hz, 1H), 3.91 (d, *J* = 10.3 Hz, 1H), 3.82 (s, 3H), 2.99 (dd, *J* = 41.6, 12.1 Hz, 2H), 2.90 (d, *J* = 9.6 Hz, 2H), 2.38 (s, 3H), 2.36 (s, 3H), 2.28 (t, *J* = 11.1 Hz, 1H), 2.12–1.96 (m, 5H), 1.93–1.82 (m, 3H), 1.75 (d, *J* = 13.0 Hz, 1H), 1.64 (dd, *J* = 14.8, 11.1 Hz, 3H). LC/MS (ESI$^+$) found *m/z* = 464.3 [M+H]$^+$ (calc for $C_{28}H_{37}N_3O_3^+$ *m/z* = 464.3 [M+H]$^+$). Purity by LC (254 nm): 100%.

*2-Amino-1-(5-methoxy-1'-methyl-6-phenylmethoxyspiro[2H-indole-3,4'-piperidine]-1-yl)ethenone (**46**)*

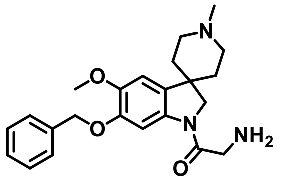

Step 1 - General Amide Coupling Method B with Boc-Gly-OH to afford the boc protected product.

Step 2 – The resulting residue was suspended in DCM (1 ml) and cooled in an ice bath to 0 °C. TFA (0.12 ml, 1.5 mmol, 15 eq) was added dropwise and the reaction left to mature cool for 1 h and at 23 °C for a further 2 h. The reaction mixture was loaded directly onto a preconditioned 2 g SCX-2 cartridge. The cartridge was thoroughly washed with MeOH before the product was eluted using 0.5 M NH_3 in MeOH. The basic wash was concentrated *in vacuo* and further dried on a Genevac overnight to afford the desired product as a yellow solid.

Yield: 29 mg (73%). ^{1}H NMR (600 MHz, DMSO-*d$_6$*) δ 7.92 (s, 1H), 7.44 (d, *J* = 7.6 Hz, 2H), 7.39 (t, *J* = 7.5 Hz, 2H), 7.32 (t, *J* = 7.3 Hz, 1H), 6.93 (s, 1H), 5.03 (s, 2H), 3.82 (s, 2H), 3.76 (s, 3H), 3.45 (s, 2H), 2.77–2.70 (m, 2H), 2.20 (s, 3H), 1.99 (t, *J* = 12.0 Hz, 2H), 1.89 (td, *J* = 12.9, 4.0 Hz, 2H), 1.50 (d, *J* = 12.7 Hz, 2H). LC/MS (ESI$^+$) found *m/z* = 396.2 [M+H]$^+$ (calc for $C_{23}H_{29}N_3O_3$ *m/z* = 396.2 [M+H]$^+$). Purity by LC (254 nm): 100%.

*(2R)-2-Amino-1-(5-methoxy-1'-methyl-6-phenylmethoxyspiro[2H-indole-3,4'-piperidine]-1-yl)-3-phenylpropan-1-one (**47**)*

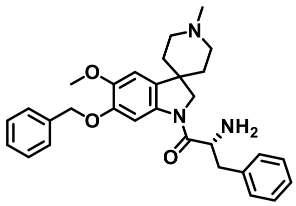

Step 1 - General Amide Coupling Method B with Boc-D-Phe-OH to afford the boc protected product.

Step 2 – The resulting residue was suspended in DCM (1 ml) and cooled in an ice bath to 0 °C. TFA (0.23 ml, 3.0 mmol, 15 eq) was added dropwise and the reaction left to mature cool for 1 h and at 23 °C for a further 2 h. The reaction mixture was loaded directly onto a preconditioned 2 g SCX-2 cartridge. The cartridge was thoroughly washed with MeOH before the product was eluted using 0.5 M NH_3 in MeOH. The basic wash was concentrated *in vacuo* and further dried on a Genevac overnight to afford the desired product as a yellow solid.

Yield: 47 mg (97%). ^{1}H NMR (600 MHz, DMSO-d_6) δ 7.98 (s, 1H), 7.45 (d, *J* = 7.4 Hz, 2H), 7.40 (t, *J* = 7.5 Hz, 2H), 7.33 (t, *J* = 7.3 Hz, 1H), 7.21 (d, *J* = 4.4 Hz, 4H), 7.15 (m, 1H), 6.85 (s, 1H), 5.08–5.00 (m, 2H), 3.92 (t, *J* = 7.3 Hz, 1H), 3.85 (d, *J* = 10.4 Hz, 1H), 3.74 (s, 3H), 3.26 (d, *J* = 10.4 Hz, 1H), 2.89–2.81 (m, 2H), 2.70 (d, *J* = 10.1 Hz, 1H), 2.59 (d, *J* = 8.8 Hz, 1H), 2.18 (s, 3H), 1.95–1.82 (m, 2H), 1.73–1.64 (m, 2H), 1.43 (d, *J* = 12.0 Hz, 1H), 0.93 (d, *J* = 10.0 Hz, 1H). LC/MS (ESI$^+$) found *m/z* = 486.4 $[M+H]^+$ (calc for $C_{30}H_{35}N_3O_3$ *m/z* = 486.3 $[M+H]^+$). Purity by LC (254 nm): 100%.

*(2S)-2-Amino-1-(5-methoxy-1'-methyl-6-phenylmethoxyspiro[2H-indole-3,4'-piperidine]-1-yl)-3-phenylpropan-1-one (**48**)*

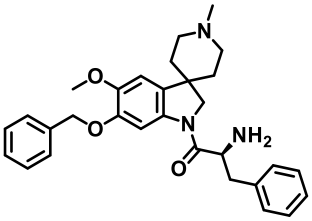

Step 1 - General Amide Coupling Method B with Boc-L-Phe-OH to afford the boc protected product.

Step 2 – The resulting residue was suspended in DCM (1 ml) and cooled in an ice bath to 0 °C. TFA (0.12 ml, 1.50 mmol, 15 eq) was added dropwise and the reaction left to mature cool for 1 h and at 23 °C for a further 16 h. The reaction mixture was loaded directly onto a preconditioned 2 g SCX-2 cartridge. The cartridge was thoroughly washed with MeOH before the product was eluted using 0.5 M NH_3 in MeOH. The basic wash was concentrated *in vacuo* and further dried on a Genevac overnight to afford the desired product as a colorless gum.

Yield: 39 mg (41%). ^{1}H NMR (600 MHz, DMSO-d_6) δ 7.99 (s, 1H), 7.45 (d, *J* = 7.5 Hz, 2H), 7.40 (t, *J* = 7.5 Hz, 2H), 7.33 (t, *J* = 7.3 Hz, 1H), 7.21 (d, *J* = 4.3 Hz, 4H), 7.15 (m, 1H), 6.85 (s, 1H), 5.07–4.99 (m, 2H), 3.86 (d, *J* = 10.4 Hz, 1H), 3.83 (dd, *J* = 8.0, 6.5 Hz, 1H), 3.74 (s, 3H), 3.30 (d, *J* = 10.4 Hz, 1H), 2.84 (dd, *J* = 13.0, 8.1 Hz,

1H), 2.78 (dd, *J* = 13.0, 6.3 Hz, 1H), 2.68 (d, *J* = 7.6 Hz, 1H), 2.60–2.56 (m, 1H), 2.17 (s, 3H), 1.92–1.82 (m, 2H), 1.72–1.63 (m, 2H), 1.48–1.41 (m, 1H), 1.00–0.93 (m, 1H). LC/MS (ESI$^+$) found *m/z* = 486.2 $[M+H]^+$ (calc for $C_{30}H_{35}N_3O_3$ *m/z* = 486.3 $[M+H]^+$). Purity by LCMS (254 nm): 100%.

1D and 2D-NMR characterization and HPLC chromatogram of compound 50:

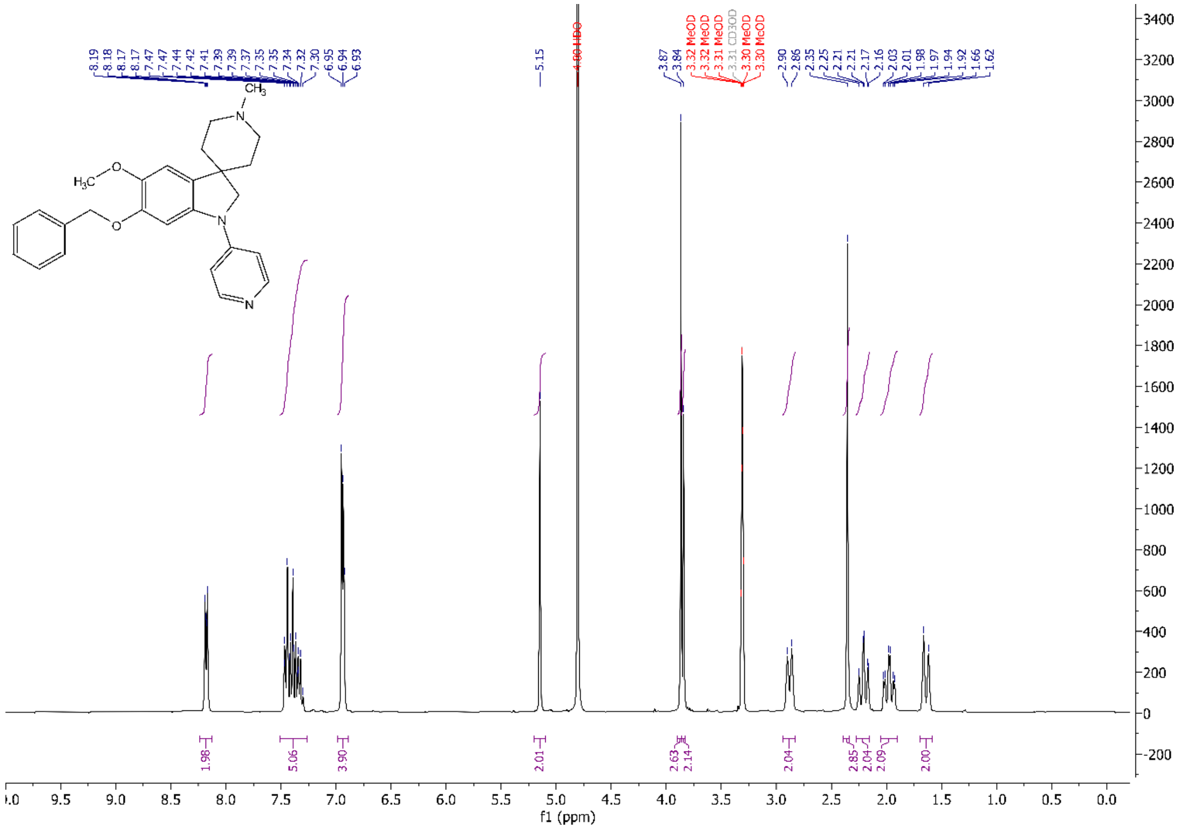

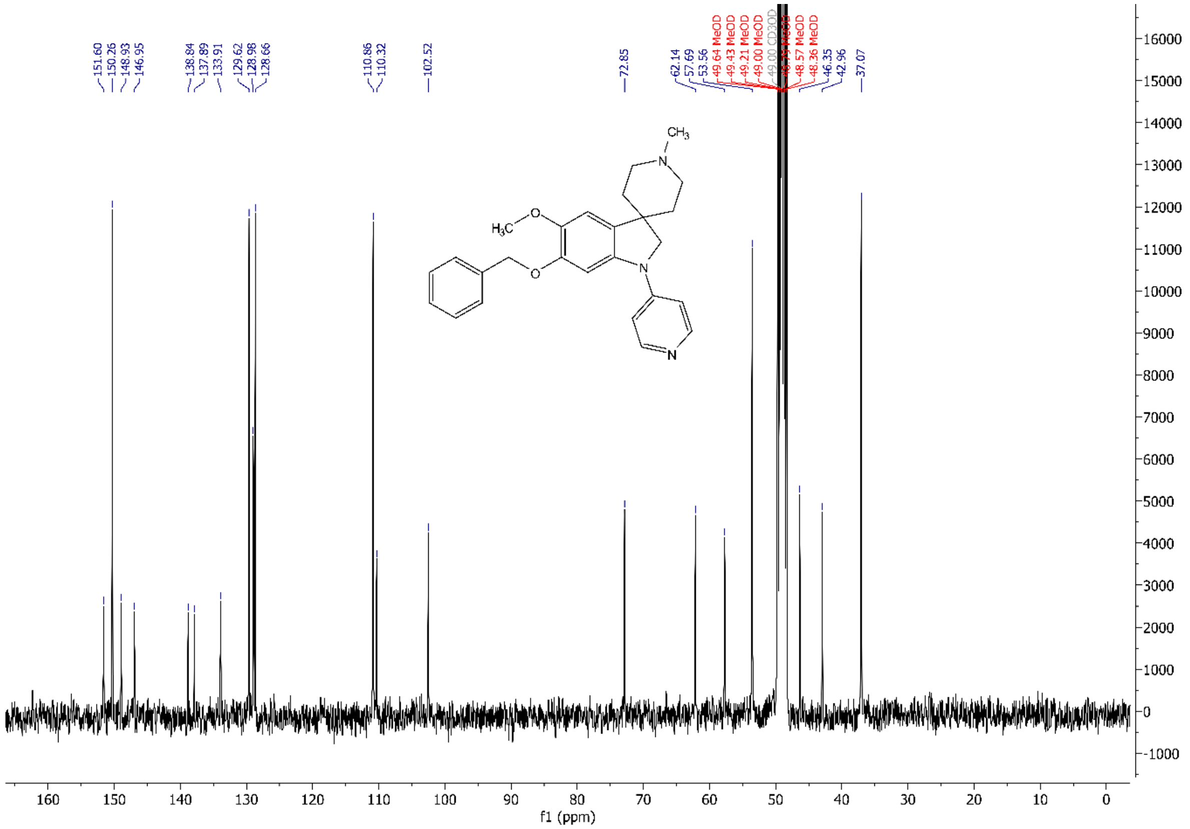
151.60
150.26
148.93
146.95
138.84
137.89
133.91
129.62
128.98
128.66
110.86
110.32
102.52
72.85
62.14
57.69
53.56
49.64 MeOD
49.43 MeOD
49.21 MeOD
49.00 MeOD
49.00 CD3OD
48.57 MeOD
48.36 MeOD
46.35
42.96
37.07
CH3
N
H3C
O
O
N
N
f1 (ppm)

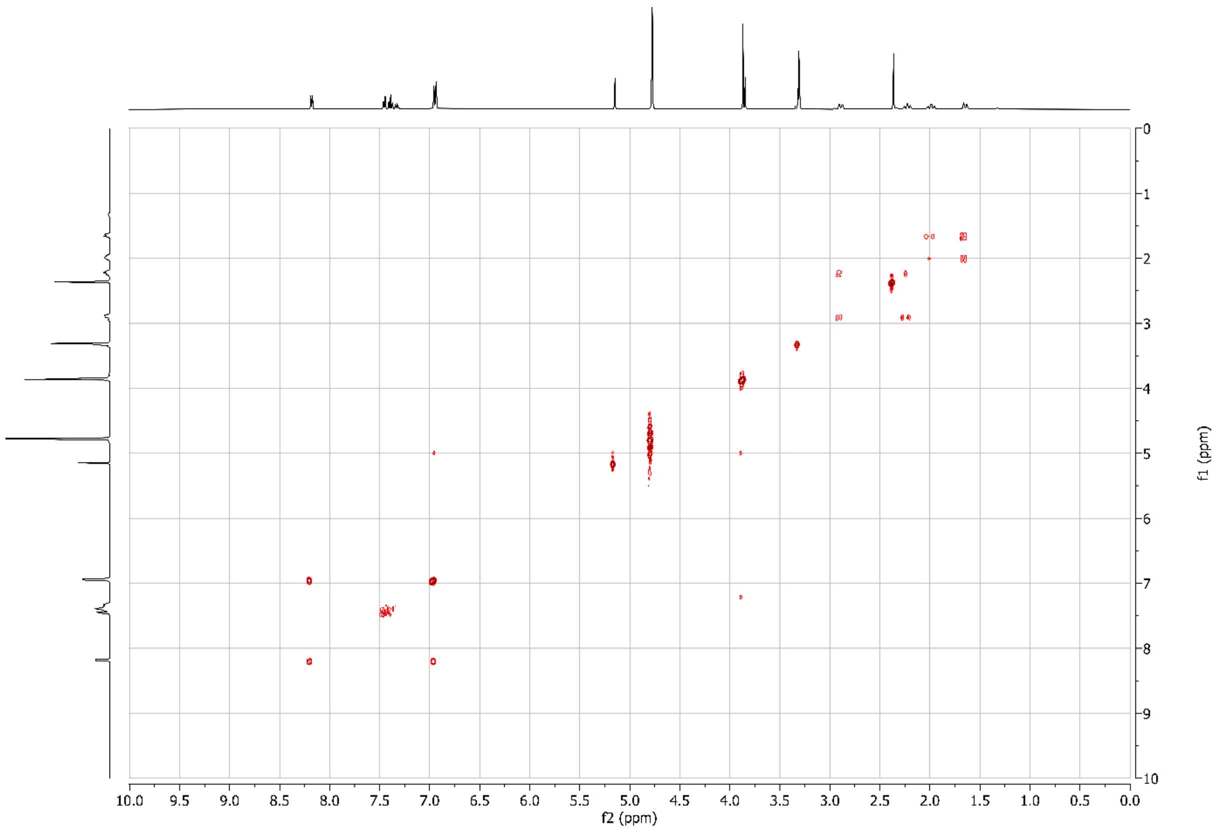
f2 (ppm)
f1 (ppm)

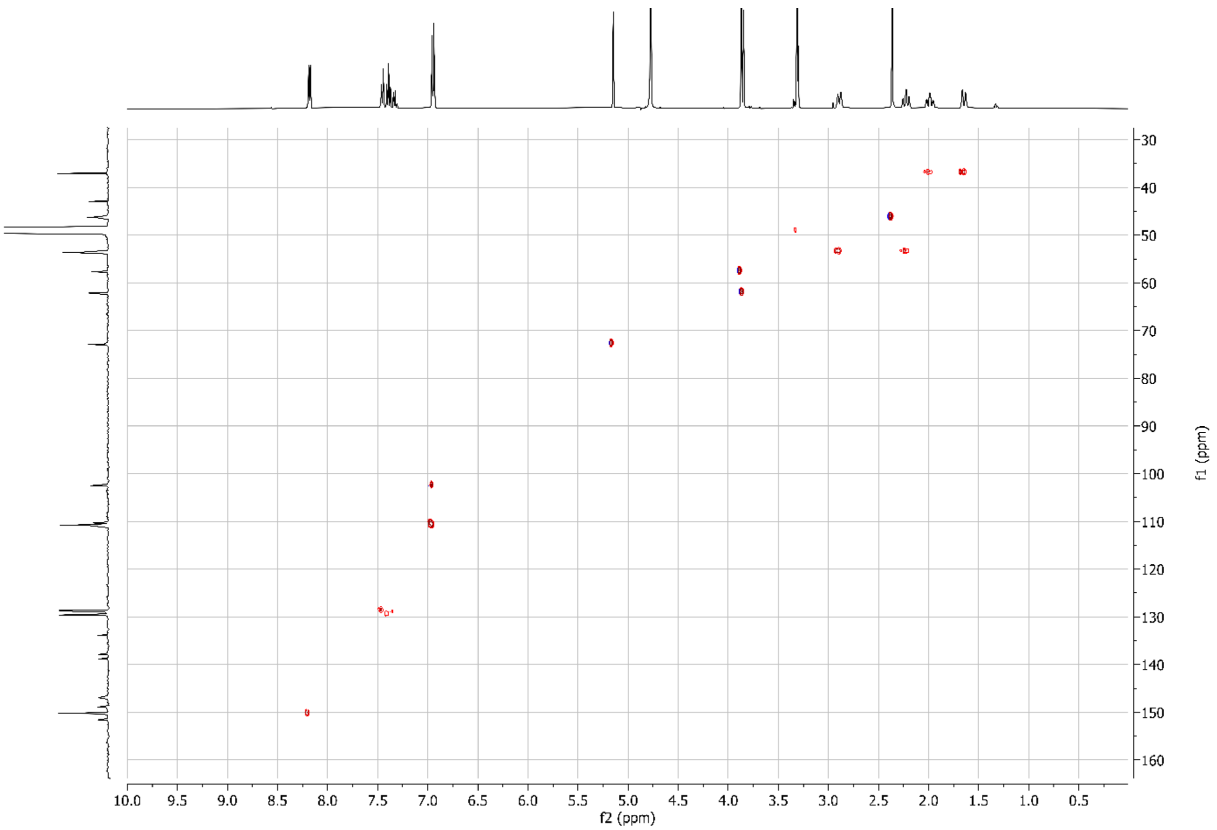

f2 (ppm)
f1 (ppm)

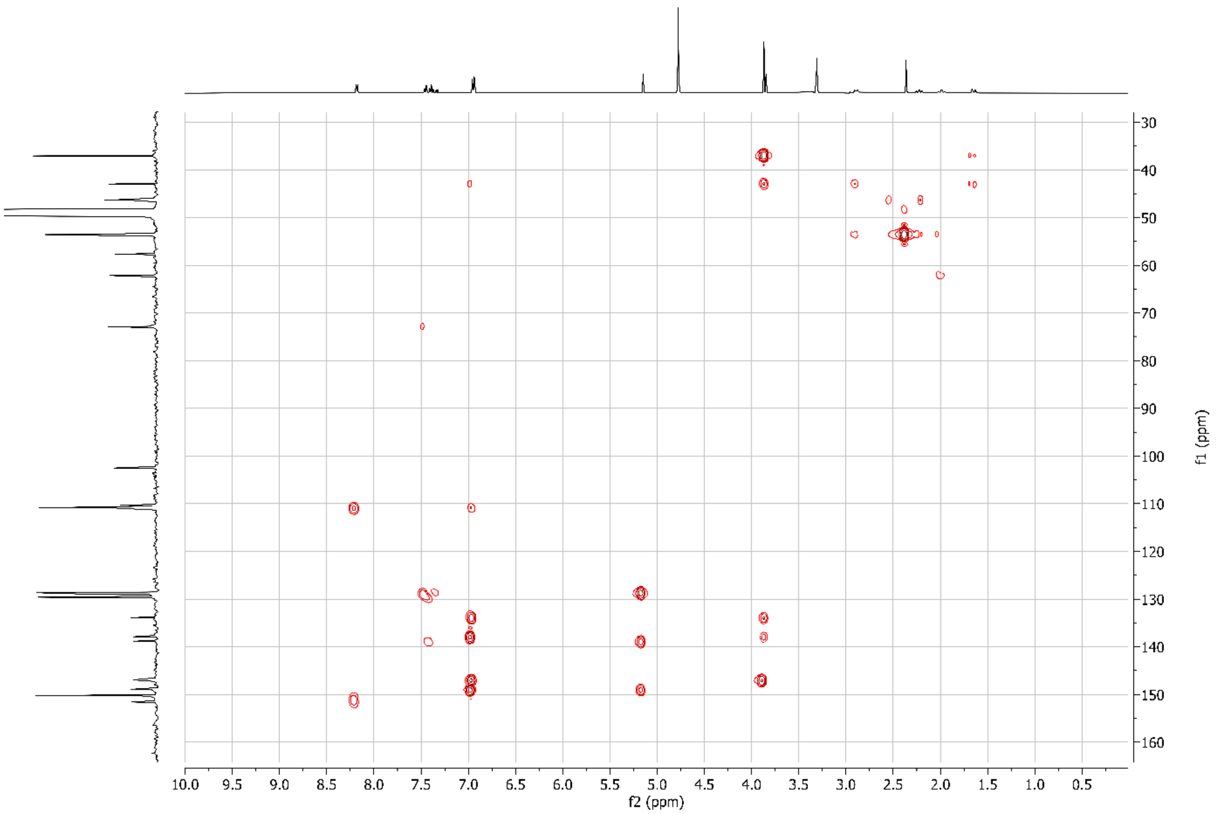

f2 (ppm)
f1 (ppm)

B. Screening Cascade

Asexual blood stage (ABS) activity and cytotoxicity

Hit compound **2** and all compounds synthesized during the hit-to-lead campaign were evaluated for *in vitro* ABS activity against the NF54 drug-sensitive strain of *P. falciparum*. Frontrunner compounds were tested against the multidrug-resistant strain *Pf* K1 to assess the potential for cross-resistance, considered when (*Pf* K1 IC_{50})/(*Pf* NF54 IC_{50}) ≥5.[1] Chloroquine and artesunate were use as reference drugs in all experiments (Section D).

Compounds with *Pf* NF54 IC_{50} < 0.50 µM (or selected for SAR reasons) were also tested for cytotoxicity against Chinese hamster ovary (CHO) cells. Selectivity Indices (SI) are reported as a ratio of cytotoxicity in relation to antiplasmodial activity (CHO IC_{50}/*Pf* NF54 IC_{50}). More information is available in Section H.

ADME Profiling

Aqueous solubility was measured in PBS at pH 6.5 for all compounds (Section L).

To assess microsomal stability, compounds with solubility >10 µM or compounds of interest were incubated with human (h), rat (r), and mouse (m) liver microsomal preparations. The intrinsic clearance $CL_{int,app}$ was derived from the percentage of compound remaining after 30 min (Section J).

Assays on request

Further assays, e.g. life cycle assays and PRR assay, were performed on request for profiling.

C. *In vitro* asexual blood stage NF54 and K1 *P. falciparum* screening

Compounds were screened against multidrug resistant (K1) and sensitive (NF54) strains of *P. falciparum in vitro* using the parasite lactate dehydrogenase assay (pLDH).[2]

Methodology

A full dose-response is performed to determine the concentration inhibiting 50% (IC_{50}) of growth using parasite lactate dehydrogenase as a marker for parasite survival. Parasites in the ring stage were prepared to 2% parasitemia and 1% hematocrit and were incubated for 72 h with a range of concentrations of each test compound.

After 72 h, the plate is re-suspended and 15 µl of the suspension is transferred to a separate microtitre plate which contains 100 µl of Malstat reagent. Then 25 µl of NBT is added to the plate. Air bubbles are removed with a hair dryer. The plate is placed in a dark cupboard to develop for approximately 5 minutes. Once a color reaction is observed from yellow to purple the plate is read using a microplate reader which measures the absorbance at 600 nm. A non-linear regression analysis is used to determine the IC_{50} value using either GraphPad Prism or the Dotmatics Software Suite.

A selection of compounds was tested at Swiss TPH in the [^{3}H]-hypoxanthine incorporation assay, as previously reported.[3]

Table S1. NF54 IC_{50} values with standard deviations for compounds measured in the LDH assay.

Entry	NF54 IC_{50} (µM) ± SD
2	0.24 ± 0.29
8	>6.0 ± n/a
11	0.26 ± 0.04
14	5.9 ± 0.03
24	4.4 ± 0.9
25	3.2 ± n/a
26	2.5 ± n/a
27	1.7 ± 0.1
28	1.7 ± 0.2
29	0.95 ± 0.06
30	0.29 ± 0.03
31	1.0 ± 0.2
32	0.34 ± 0.04
33	2.2 ± 0.2
34	0.93 ± 0.03
35	0.44 ± 0.30
36	0.37 ± 0.01
37	0.53 ± 0.01
38	2.1 ± 0.6

39	2.1 ± 0.7
40	3.8 ± 0.7
41	0.23 ± 0.04
42	0.28 ± 0.10
43	0.13 ± 0.02
44	2.5 ± 1.1
45	3.8 ± 0.5
46	1.9 ± 1.1
47	0.50 ± 0.17
48	0.40 ± 0.17
49	>6.0 ± n/a
50	0.088 ± 0.014

D. Cross-resistance screening

Compounds were screened against field multidrug resistant (K1, 7G8, TM90C2B, RF12, Dd2) and sensitive (NF54) strains and a laboratory-generated resistant Dd2 mutant panel of *P. falciparum* in vitro using the modified [^{3}H]-hypoxanthine incorporation assay.[3]

Methodology

Plasmodium falciparum was cultivated in a variation of the medium previously described,[4] consisting of RPMI 1640 supplemented with 0.5 % ALBUMAX® II, 25 mM Hepes, 25 mM $NaHCO_3$ (pH 7.3), 0.36 mM hypoxanthine, and 100 µg/ml neomycin. Human erythrocytes served as host cells. Cultures were maintained at 37 °C in an atmosphere of 3% O_2, 4% CO_2, and 93% N_2 in humidified modular chambers. Compounds were dissolved by sonication in DMSO (10 mg/ml) and diluted in hypoxanthine-free culture medium. Infected erythrocytes (100 microliter per well with 2.5% hematocrit and 0.3% parasitemia) were added to each drug titrated in 100 microliter duplicates over a 64-fold range. After 48 h incubation, 0.5 microCi of [^{3}H]hypoxanthine in 50 microliter medium was added and plates were incubated for an additional 24 h. Parasites were harvested onto glass-fiber filters and radioactivity was counted using a Betaplate liquid scintillation counter (Wallac, Zurich). The results were recorded as counts per minute (cpm) per well

at each drug concentration and expressed as a percentage of the untreated controls. Fifty percent inhibitory concentrations (IC_{50}) were estimated by linear interpolation.[5]

E. Stage Specificity

In vitro antiplasmodial blood stage activity was measured using the [^{3}H]-hypoxanthine incorporation assay, which measures dose-dependent drug inhibition of P. falciparum growth.[3] Results were expressed as the concentration resulting in 50% inhibition (IC_{50}). In vitro time-, stage-, and concentration-dependent effects were assessed using pyrimethamine as a stage-specific and slow acting control.[6]

Table S2. Activity (mean values, from 2 independent biological replicates) against ring and schizonts of the NF54 parasite strain using the [^{3}H]-Hypoxanthine incorporation assay.

	**Compound 2**	**Compound 50**
NF54 ring IC_{50} (µM)	0.38	0.058
NF54 schizont IC_{50} (µM)	0.40	0.19

Figure S1 – Stage specificity. In the tested concentration range (100x-1,5x the IC_{50}), the compound **50** showed stronger action against young ring forms after 24 h compound incubation.

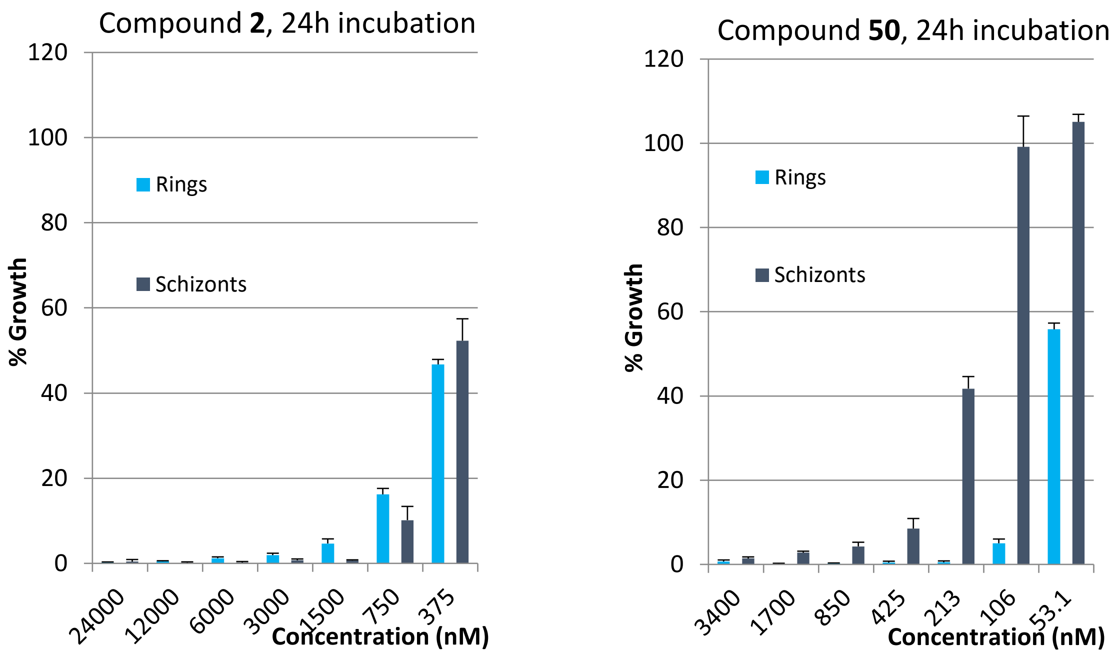

F. Estimation of antimalarial killing profile using two-color flow cytometry analysis (GSK, Tres Cantos)

Methodology

The assay uses a limiting dilution technique to quantify the number of parasites that remain viable after drug treatment.

P. falciparum strain 3D7 is treated with the selected drug at concentration corresponding to 10 x IC_{50}.

Parasites are treated for 48 h. Drug is renewed daily over the entire treatment period. Samples of parasites are taken from the treated culture every 24 h (0, for the control of initial number of parasites, 24 and 48 h time points), drug is washed out and drug-free parasites are cultured in 96 well plates by adding fresh erythrocytes and new culture media.

To quantify the number of viable parasites after treatment, 3-fold serial dilution is used with the above-mentioned samples after removing the drug. Parasites are cultured in microtiter plates to allow all wells with viable parasites, to render detectable parasitemia. Four independent serial dilutions are done with each sample to correct experimental variations. After 18 days of culturing, samples are taken to examine growth. Additional sampling is done after 22 days to confirm growth/ no growth.

The number of viable parasites is determined by counting the number of wells with growth.

The experimental number of viable parasites after each treatment is back-calculated by using the formula X^{n-1} where n is the number of wells able to render growth and X the dilution factor (when n=0 number of viable parasites is estimated as zero). The number of initial parasites (no treatment) is calculated in a similar manner and used to calculate a normalization factor to correct the deviation of the experimental determination from the theoretical number of initial parasites (10^5, 100 µl from a 10^6 parasites/ml inoculum). Normalization factor is used to correct all the experimental data for a starting number of parasites equal to 10^5 and enables comparison between different experiments.

Pyrimethamine (a standard antimalarial drug) is included in each assay to validate the results produced and allow comparative classification of the killing behavior of the drugs tested.

Results

The normalized log reduction in parasitemia after 24 and 48 h treatment with compound **2** (MMV909980), **43** (MMV1794381) and **50** (MMV1794822) have been determined and referred to control samples (non-treated parasites).

Normalized log reduction in parasitemia was also calculated for pyrimethamine as a control and worked as expected. Compounds **2** (MMV909980), **43** (MMV1794381) and **50** (MMV1794822) were then compared to the normalized log reduction of four standard antimalarials (artemisinin, atovaquone, pyrimethamine and chloroquine) to determine

the rate of kill for the compounds. Compound **2** displayed a slow killing profile (similar to atovaquone) while compounds **43** and **50** displayed a fast-killing profile (similar to chloroquine).

Table S3. Normalized log reduction in parasitemia in 3D7 strain of *P. Falciparum* following treatment of compounds **2**, **43** and **50** at 0, 24 and 48 h

	Time treatment (h)					
	**0**		**24**		**48**	
	**Log(viable parasites +1) Normalized**	**SD**	**Log(viable parasites +1) Normalized**	**SD**	**Log(viable parasites +1) Normalized**	**SD**
**2**	5.00	0.37	4.51	0.47	3.78	0.61
**43**	5.00	0.33	2.00	0.23	0.44	0.17
**50**	5.00	0.33	2.81	0.38	0.64	0.40
Pyrimethamine	5.00	0.37	4.51	0.39	2.20	0.28

G. Determination of antimalarial killing profile using a parasite reduction ratio (PRR) assay (GSK, Tres Cantos)

Methodology

The assay uses limiting dilution technique to quantify number of parasites that remain viable after drug treatment. *P. falciparum* strain 3D7 is treated with the selected drug at concentration corresponding to 10xIC_{50}.

Parasites are treated for 120 hours. Drug is renewed daily over the entire treatment period. Samples of parasites are taken from the treated culture every 24 hours (0, for the control of initial number of parasites, 24, 48-, 72-, 96- and 120-hour time points), drug is washed out and drug-free parasites are cultured in 96 well plates by adding fresh erythrocytes and new culture media.

To quantify number of viable parasites after treatment, 3-fold serial dilution is used with the above-mentioned samples after removing the drug. Parasites are cultured in microtiter plates to allow all wells with viable parasites, to render detectable parasitemia. Four independent serial dilutions are done with each sample to correct experimental variations. After 18 days of culturing, samples are taken to examine growth. Additional sampling is done after 22 days to confirm growth/ no growth.

The number of viable parasites is determined by counting the number of wells with growth.

The number of viable parasites is back-calculated by using the formula Xn-1 where n is the number of wells able to render growth and X the dilution factor (when n=0 number of viable parasites is estimated as zero).

The experimental number of viable parasites after each treatment is back-calculated by using the formula X^{n-1} where n is the number of wells able to render growth and X the dilution factor (when n=0 number of viable parasites is estimated as zero). The number of initial parasites (no treatment) is calculated in a similar manner and used to calculate a normalization factor to correct the deviation of the experimental determination from the theoretical number of initial parasites (10^5, 100 µl from a 10^6 parasites/ml inoculum). Normalization factor is used to correct all the experimental data for a starting number of parasites equal to 10^5 and enables comparison between different experiments.

Additional parameters are calculated such as lag phase, time needed to observe the maximal killing effect of the drug being tested, PRR (parasite reduction ratio), such as the number of parasites the drug can kill in a parasite life cycle and $PCT_{99.9\%}$ (parasite clearance time), such as time the drug tested requires to kill 99.9% of the initial population.

Pyrimethamine is used as control and results used to validate the experiment.

Results

Table S4. Parasite viability for full PRR assay of compound **2**.

**Compound 2 (10x IC_{50})**					
**Time**	**Log (viable parasites +1)** ***experimental data***	**Std dev** ***experimental data***	**Normalization factor**	**Log (viable parasites +1)** ***Normalized data***	**Std dev** ***Normalized data***
0	5.09	0.00	0.98	5.00	0.00
24	4.65	0.46		4.57	0.45
48	2.98	0.24		2.93	0.23
72	0.80	0.23		0.79	0.23
96	0.12	0.00		0.00	0.00
120	0.12	0.00		0.00	0.00

Table S5. Parasite viability for full PRR assay of compound **50**.

Compound 50 (10x IC_{50})					
**Time**	**Log (viable parasites +1)** *experimental data*	**Std dev** *experimental data*	**Normalization factor**	**Log (viable parasites +1)** *Normalized data*	**Std dev** *Normalized data*
0	5.09	0.00	0.98	5.00	0.00
24	2.63	0.61		2.58	0.60
48	0.55	0.33		0.54	0.33
72	0.12	0.00		0.12	0.00
96	0.29	0.23		0.28	0.22
120	0.12	0.00		0.00	0.00

Table S6. Summary of PRR parameters for compounds **2** and **50**.

Compound	Dose	Lag phase (h)	Slope	R	Log PRR	PCT99.9% (h)
**Compound 2 10xIC_{50}**	10x IC50	24	-0.08	1	3.8	58
**Compound 50 10xIC_{50}**	10x IC50	0	-0.09	1	4.5	32
Control						
Pyrimethamine	10x IC50	24	-0.07	1	3.6	61

* Pyrimethamine used as internal control worked as expected and validated the results of this experiment.

H. Cytotoxicity screening

Compounds were tested for *in vitro* cytotoxicity against two mammalian cell-lines, Chinese Hamster Ovarian (CHO) using the 3-(4,5-dimethylthiazol-2-yl)-2,5-diphenyltetrazoliumbromide (MTT)-assay.

Methodology

Compounds were tested in triplicate on a single occasion against these cells using the MTT assay as described. [7,8] The test samples were prepared to a 10 mmol/L stock solution in 100% DMSO. Samples were tested as a suspension if

not completely dissolved. Further dilutions to the desired starting concentration were freshly prepared in growth media at the start of the experiment.

Cells were plated to a density of 10^5 cells/well in 96-well plates and allowed to attach for 24 h. After that compounds were added at various concentrations from 50 mM down to 16 nM and the cells incubated for a further 48 h. Emetine was used as the control compound, since it shows non-specific cytotoxicity to mammalian cells. After 44 h MTT was added, and plates allowed to develop for a further 4 h at 37 °C. Plates were then centrifuged at 200 RPM for 5 minutes to pellet the reduced dye crystals, and the growth medium carefully removed by aspiration. Crystals were solubilised using 50 ml of DMSO, and the plates were gently shaken to ensure complete dissolution then read at 540 nm on a spectrophotometer. Survival was plotted against concentration and the IC_{50} values were obtained using a non-linear dose-response curve fitting analysis via either Graphpad Prism or the Dotmatics software platform.

I. hERG patch clamp assay and data

Compounds were tested for inhibition of the human ether a go-go related gene (hERG) K^+ channel using B'Sys automated whole cell patch clamp electrophysiology. 8-Point concentration-response curves were generated using 3-fold serial dilutions from the maximum final assay concentration. IC_{50}'s were determined from the curves.

J. Metabolic stability - Microsome $CL_{int,app}$

Methodology - Liver microsomes

The metabolic stability assay was performed in duplicate in a 96-well microtitre plate. The test compounds (1 µM) were incubated (37 °C) in mouse, rat and human liver microsomes (final protein concentration of 0.4 mg/mL; XenoTech, Lenexa, KS) suspended in 0.1 M phosphate buffer (pH 7.4) for 30 minutes, in the presence of the cofactor NADPH (1 mM). The reactions were quenched by the addition of ice-cold acetonitrile containing internal standard (carbamazepine, 0.0236 µg/mL). The samples were centrifuged, and the supernatant was analyzed by LC-MS/MS (Agilent Rapid Resolution HPLC, AB SCIEX 4500 MS). The relative loss of parent compound over time was monitored and the first order rate constant for compound depletion was used to calculate the degradation half-life, the *in vitro* intrinsic clearance value $CL_{int,in\ vitro}$ and subsequently a predicted *in vivo* intrinsic apparent $CL_{int,app}$ (without taking incubational binding into account) value, and the hepatic extraction ratio E_H using the following formulas: [9]

$t_{1/2}$ (min) = 0.693/k

$CL_{int,in\ vitro}$ = k/microsomal protein content (0.4 mg protein/mL)

$CL_{int,app}$ = $CL_{int,\ in\ vitro}$ x [mg microsomal protein/g liver mass] x [liver mass (g)/body weight (kg)]

$E_H = CL_{int,app}/(CL_{int,app}+Q)$

Table S7. Values used for calculations of $CL_{int,app}$.

Species	Liver Weight (g/kg Body Weight)	Liver Blood Flow (ml/min/kg)
Mouse	88	90.0
Rat	40	55.2
Dog	32	30.9

Results

Table S8: Microsomal stability data for compounds with NF54 IC_{50} < 0.5 µM and solubility > 10 µM

Entry	Human $CL_{int,app}$ (mL/min/kg)	Rat $CL_{int,app}$ (mL/min/kg)	Mouse $CL_{int,app}$ (mL/min/kg)
**11**	< 10.4	< 20.9	< 54.7
**31**	< 10.4	38.4	45.7
**32**	155	345	710
**35**	< 10.4	34.3	72.9
**36**	< 10.4	28.0	70.8
**41**	< 10.4	< 20.9	45.7
**42**	< 10.4	230	248
**47**	75.2	90.2	156
**48**	< 10.4	< 20.9	46.9

Results are given as a mean number of duplicates.

K. CYP inhibition assay

Methodology

Cytochrome P450 (CYP450) inhibition was evaluated in human liver microsomes using CYP-specific substrates and inhibitors.[10] The incubations contained test compounds (0 – 25 µM), 0.16 mg/ml HLM (CYP2C9, CYP2D6, CYP3A4) or 0.5 mg/ml HLM (CYP2C19) and the relevant CYP450 substrates at sub-Km concentrations, all in phosphate buffer pH 7.4 prewarmed at 37 °C. The reactions were initiated by addition of 1mM NADPH as cofactor and incubated at 37 °C for 15 min (CYP2C9, CYP2D6, CYP3A4) or 60 min (CYP2C19). Incubations were terminated by addition of acetonitrile containing internal standard (carbamazepine, 0.0236 µg/mL) and centrifuged (5000 x g, 15 min). The

supernatant was transferred to a 96-well plate and analysed by LC-MS/MS (Agilent Rapid Resolution HPLC coupled to AB SCIEX 4500 MS). The relative reduction in the formation of the CYP-specific metabolite in each reaction over time was monitored and used to determine the IC50. The CYP reaction pathways monitored were, diclofenac 4'-hydroxylation (CYP2C9), Mephenytoin 4'-hydroxylation (CYP2C19), Dextromethorphan O-demethylation (CYP2D6) and Midazolam 1'-hydroxylation (CYP3A4). Known inhibitors of the CYP450 enzymes (CYP2C9 – Sulphaphenazole, CYP2C19 – Ticlopidine, CYP2D6 – Quinidine, CYP3A4 – Ketoconazole) were used as positive controls.

L. Aqueous solubility assay

Methodology[11]

The solubility assay was performed using a miniaturized shake flask method. 10 mM stock solutions of each of the test compounds were used to prepare calibration standards (10-220 µM) in DMSO, and to spike (1:50) duplicate aqueous samples of phosphate buffered saline (pH 6.5), 0.01 M HCL (pH 2) and FaSSIF (simulating fasting state biorelevant media, pH 6.5). DMSO was dried down over 2 h in a genevac (MiVac GeneVac, 90 min, 37 ˚°C). After shaking for 20 h at 25 °C, the solutions were filtered and analyzed by means of HPLC-DAD (Agilent 1200 Rapid Resolution HPLC with a diode array detector). Best fit calibration curves were constructed using the calibration standards, which were used to determine the aqueous samples' solubility.[12]

M. Metabolite Identification

The test compound (0.1 µM) was incubated at 37 °C in a solution containing 0.4 mg/ml microsomes (MLM; male mouse CD1, lot No.131.210 Xenotech, RLM; male rat IGS, lot No.1510115 Xenotech; HLM; mixed gender, lot No.181003 Xenotech) and NADPH (1 mM) in potassium phosphate buffer (100 mM, pH 7.4) for 30 minutes while shaking. The samples were then extracted by ice-cold acetonitrile precipitation, centrifuged and the supernatant was analysed by LC-MS/MS.

Propanolol and midazolam were incubated concomitantly as positive controls; MMV390048 as a negative control.

Metabolites formed in microsomal incubations of the compound were identified by comparison of the T30 chromatograms with the controls chromatograms and by comparison of the product ion spectra of the $[M+H]^+$ ions of the metabolites with that of the parent compound using Analyst 1.6.2 and using synthetic standard when available.

Table S9: LC-MS/MS analytical conditions

Instrument	AB SCIEX 4500 QTRAP equipped with a Turbo V™ ion source coupled to a Agilent 1260 HPLC
Detection	Positive electrospray ionization (ESI) under EMS-IDA-EPI scans, Precursor Ion scans, Neutral Loss scans, MRM-IDA-EPI
Column	Poroshell 120 EC-C18, 50 x 4.6 mm 2.7 µm particles (Agilent)
LC conditions	Gradient 0.5 mL/min, 10 min run, injection volume 10 µL, column temperature 40 °C, sample tray temperature 8 °C
Mobile phase	A: 0.1% formic acid; B: 0.1% formic acid in acetonitrile
Software	Analyst 1.6.2 software for instrument control and data acquisition<br>LightSight 2.2 software for metabolite identification data processing

The main biotransformations in liver microsomes were demethylation and oxidation (Table S5). According to their fragmentation patterns, P+16 derived from oxidation of the indole substituent (R^2). The fragmentation pattern of the demethylation metabolites P-14 (I) and P-14 (II) were not conclusive of the position of the demethylation.

Table S10: Metabolites of Compound 2 identified in liver microsomes

Metabolite Description*	Metabolite Code	RT (min)	Detected in	$[M + H]^+$ (m/z)	Diagnostic ions (*m/z*)	Tentative Identity
Parent	P	6.60	HLM, RLM, MLM	482	391<br>339<br>144/116<br>91	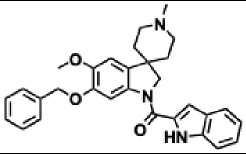
Demethylation	P-14 (I)	6.40	HLM, RLM, MLM	468	377<br>325<br>144/116<br>-	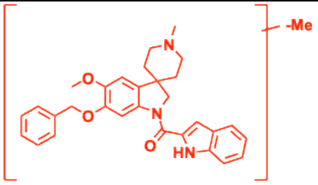
Demethylation	P-14 (II)	6.55	HLM, RLM, MLM	468	377<br>325<br>144/116<br>91	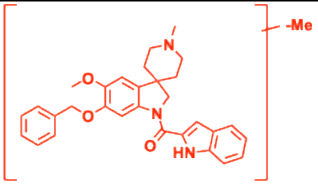
Dehydrogenation	P-2	7.75	HLM, RLM, MLM	480	389<br>337<br>144/116<br>91	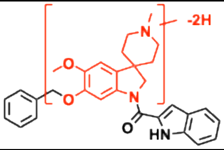
Oxidation	P+16	6.10	HLM, RLM, MLM	498	407<br>339<br>160/132<br>91	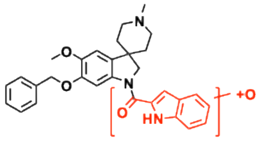

*Metabolites which had % area > 1% as determined by Lightsight % were reported

N. PK studies – Methods

Prior to animal studies being carried out, the research protocol was evaluated and approved by the Animal Ethics Committee at the University of Cape Town (AEC017_026).

Animal experiments and formulations

The compounds were dissolved in DMA/PPG/PEG: 10/60/30 for iv dose at 3 mg/kg and in 0.5% (w/v) hydroxypropylmethylcellulose (HPMC) with 0.2% Tween80 water solution for oral dose at 10 mg/kg administered to male BALB/c mice (n=3 for each group).

PK sampling

Blood samples were collected at predetermined sampling times (0.17 h, 0.5 h, 1 h, 3 h, 5 h, 7 h, 9 h, 24 h for intravenous dosing; 0.5 h, 1 h, 3 h, 5 h, 7 h, 9 h, 24 h for oral dosing) via tail bleeding in heparinized tubes and were stored at -80 °C until extraction.

Sample preparation

Frozen whole blood samples were thawed and 30 µL were extracted by protein precipitation using 200 µL of acetonitrile containing 0.1% of formic acid and 10 ng/ml of internal standard (MMV394902); vortexed and centrifuged. Calibration standards and quality controls were extracted following the same procedure. Supernatants were injected onto the column for LC-MS/MS analysis.

LC-MS/MS analytical conditions

Table S11: LC-MS/MS analytical conditions

Instrument	AB SCIEX 4500 QTRAP or 5500 QTRAP equipped with a Turbo V™ ion source coupled to a Agilent 1260 HPLC
Detection	Positive electrospray ionization under MRM scans
Column	Atlantis T3, 50 x 2.1 mm 5.0 µm particles (MMV1795000)<br>Poroshell 120 EC-C18, 50 x 4.6 mm 2.7µm particles (Agilent) (MMV1793725, MMV1796166, MMV1797227)
LC conditions	Gradient 0.4-0.6 mL/min, 7-9 min run, injection volume 5-10 µL, column temperature 40 °C, sample tray temperature 8 °C
Mobile phase	A: 0.1% formic acid; B: 0.1% formic acid in acetonitrile
Software	Analyst 1.6.2 software for instrument control and data acquisition

PK analysis

A non-compartmental analysis was performed for the determination of the pharmacokinetic parameters using PK Solutions v2.0 (Summit Research Services).

O. *In vivo* antimalarial efficacy studies with *P. falciparum* conducted at H3D

Prior to animal studies being carried out, the research protocol was evaluated and approved by the Animal Ethics Committee at the University of Cape Town (AEC017_025).

The therapeutic efficacy of compound **50** following a single oral dose was evaluated in the NSG mouse model for malaria as previously described.[13] Briefly, NOD-*scid IL-2Rynull* mice engrafted with human erythrocytes (approximately 60%) were infected with 2 x 10^7 *P. falciparum*- infected erythrocytes from a donor mouse. Infections were done via intravenous injection (day 0). Treatment commenced on day 3 and ended on day 7 following infection. In all cases, parasitemia was assessed in samples from peripheral blood obtained on days 3, 4, 5, 6 and 7 after infection. Fresh samples of peripheral blood from *P. falciparum*-infected mice were stained with TER-119-PE (marker for murine erythrocytes) and SYTO-16 (nucleic acid dye) and then analyzed via flow cytometry.

The whole blood concentrations of the **50** were determined using a quantitative LC-MS/MS method. Sample preparation was achieved with a protein precipitation extraction method, using 10 µL whole blood and 100 µL ACN containing the internal standard (MMV394902). The experimental data were evaluated in terms of drug concentration versus time. Non-compartmental analysis was used to calculate the PK parameters of **50** using PK Solutions v2.0 (Summit Research Services).

Table S12. Pharmacokinetic parameters of compound **50** in the NSG mouse model of *P. falciparum* infection.

Values are presented as the mean ±SD[LG1]

**Parameter**	**35 mg/kg**	**10 mg/kg**	**4 mg/kg**
C_{max} (µM)	0.35 (0.15)	0.08 (0.00)	0.06 (0.00)
AUC_{0-96} (µM.h)	19.03 (9.67)	5.28 (1.25)	1.92 (0.00)

T_{max} (h)	48 (34)	26 (32)	3 (0)

References

(1) Chugh, M.; Scheurer, C.; Sax, S.; Bilsland, E.; van Schalkwyk, D.A.; Wicht, K.J.; Hofmann, N.; Sharma, A.; Bashyam, S.; Singh, S.; Oliver, S.G.; Egan, T.J.; Malhotra, P.; Sutherland, C.J.; Beck, H.P.; Wittlin, S.; Spangenberg, T.; Ding, X.C. Identification and deconvolution of cross-resistance signals from antimalarial compounds using multidrug-resistant Plasmodium falciparum strains. *Antimicrob Agents Chemother.* **2015**, *59* (2), 1110-1118. DOI: 10.1128/AAC.03265-14. Epub 2014 Dec 8. PMID: 25487796; PMCID: PMC4335906.

(2) Makler, M. T.; Ries, J. M.; Williams, J. A.; Bancroft, J. E.; Piper, R. C.; Gibbins, B. L.; Hinrichs, D. J. Parasite lactate dehydrogenase as an assay for Plasmodium falciparum drug sensitivity. *Am J Trop Med Hyg* **1993**, *48* (6), 739-741. DOI: 10.4269/ajtmh.1993.48.739.

(3) Snyder, C.; Chollet, J.; Santo-Tomas, J.; Scheurer, C.; Wittlin, S. In vitro and in vivo interaction of synthetic peroxide RBx11160 (OZ277) with piperaquine in Plasmodium models. *Exp Parasitol* **2007**, *115* (3), 296-300. DOI: 10.1016/j.exppara.2006.09.016. Duffey, M.; Blasco, J. N.; Wells, T. N. C.; Fidock, D. A.; Leroy, D.; Assessing risks of Plasmodium falciparum resistance to select next-generation antimalarials, *Trends in Parasitology*, **2021**, *37* (8), 709-721, DOI: 10.1016/j.pt.2021.04.006.

(4) Dorn, A.; Stoffel, R.; Matile, H.; Bubendorf, A.; Ridley, R. G. Malarial haemozoin/beta-haematin supports haem polymerization in the absence of protein. *Nature* **1995**, *374* (6519), 269-271. DOI: 10.1038/374269a0.

(5) Huber, W.; Koella, J. C. A comparison of three methods of estimating EC50 in studies of drug resistance of malaria parasites. *Acta Trop* **1993**, *55* (4), 257-261. DOI: 10.1016/0001-706x(93)90083-n.

(6) Le Manach, C.; Scheurer, C.; Sax, S.; Schleiferböck, S.; Gonzalez Cabrera, D.; Younis, Y.; Paquet, T.; Street, L. J.; Smith, P.; Ding, X. C.; Waterson, D.; Witty, M. J.; Leroy, D.; Chibale, K.; Wittlin, S.; Fast in vitro methods to determine the speed of action and the stage specificity of anti-malarials in Plasmodium falciparum. *Malar. J.* **2013**, *12*, 424–430.

(7) Mosmann, T. Rapid colorimetric assay for cellular growth and survival: application to proliferation and cytotoxicity assays. *J Immunol Methods* **1983**, *65* (1-2), 55-63. DOI: 10.1016/0022-1759(83)90303-4.

(8) Rubinstein, L. V.; Shoemaker, R. H.; Paull, K. D.; Simon, R. M.; Tosini, S.; Skehan, P.; Scudiero, D. A.; Monks, A.; Boyd, M. R. Comparison of in vitro anticancer-drug-screening data generated with a tetrazolium assay versus a protein assay against a diverse panel of human tumor cell lines. *J Natl Cancer Inst* **1990**, *82* (13), 1113-1118. DOI: 10.1093/jnci/82.13.1113.

(9) Obach, R. S. Prediction of human clearance of twenty-nine drugs from hepatic microsomal intrinsic clearance data: An examination of in vitro half-life approach and nonspecific binding to microsomes. *Drug Metab Dispos* **1999**, *27* (11), 1350-1359.

(10) Walsky, R.L.; Obach, R.S. Validated assays for human cytochrome P450 activities *Drug Metabolism and Disposition*, *32* (6), 647-660

(11) Hill, A. P.; Young, R. J. Getting physical in drug discovery: a contemporary perspective on solubility and hydrophobicity. *Drug Discov Today* **2010**, *15* (15-16), 648-655. DOI: 10.1016/j.drudis.2010.05.016.

(12) Alelyunas, Y. W.; Liu, R.; Pelosi-Kilby, L.; Shen, C. Application of a Dried-DMSO Rapid Throughput 24-h Equilibrium Solubility in Advancing Discovery Candidates. *Eur. J. Pharm. Sci.* **2009**, 37 (2), 172–182. https://doi.org/10.1016/j.ejps.2009.02.007.

(13) Angulo-Barturen, I.; Jiménez-Díaz, M. B.; Mulet, T.; Rullas, J.; Herreros, E.; Ferrer, S.; Jiménez, E.; Mendoza, A.; Regadera, J.; Rosenthal, P. J.; et al. A Murine Model of falciparum-Malaria by In Vivo

Selection of Competent Strains in Non-Myelodepleted Mice Engrafted with Human Erythrocytes. *PLOS ONE* **2008**, *3* (5), e2252. DOI: 10.1371/journal.pone.0002252.
